# Peaked-to-flat transition in quasispecies structure evolution

**DOI:** 10.1093/ve/veag024

**Published:** 2026-04-14

**Authors:** Josep Gregori, Sergi Colomer-Castell, Carolina Campos, Marta Ibañez-Lligoña, Damir García-Cehic, Maria Francesca Cortese, David Tabernero, Mar Riveiro-Barciela, Maria Buti, Ariadna Rando-Segura, Roser Ferrer, Tomàs Pumarola, Cristina Andrés, Alejandra González-Sánchez, Andrés Antón, Francisco Rodriguez-Frias, Josep Quer

**Affiliations:** Liver Diseases-Viral Hepatitis, Liver Unit, Vall d’Hebron Institute of Research (VHIR), Instituto de Investigación Sanitaria Hospital Universitari Vall d’Hebron (IIS IR-HUVH), Vall d’Hebron Barcelona Hospital Campus, Passeig Vall d’Hebron 119–129, 08035 Barcelona, Spain; Liver Diseases-Viral Hepatitis, Liver Unit, Vall d’Hebron Institute of Research (VHIR), Instituto de Investigación Sanitaria Hospital Universitari Vall d’Hebron (IIS IR-HUVH), Vall d’Hebron Barcelona Hospital Campus, Passeig Vall d’Hebron 119–129, 08035 Barcelona, Spain; Centro de Investigación Biomédica en Red de Enfermedades Hepáticas y Digestivas (CIBEREHD), Instituto de Salud Carlos III, Av. Monforte de Lemos, 3–5, 28029 Madrid, Spain; Biochemistry and Molecular Biology Department, Universitat Autònoma de Barcelona (UAB), Campus de la UAB, Plaça Cívica, 08193 Bellaterra, Spain; Liver Diseases-Viral Hepatitis, Liver Unit, Vall d’Hebron Institute of Research (VHIR), Instituto de Investigación Sanitaria Hospital Universitari Vall d’Hebron (IIS IR-HUVH), Vall d’Hebron Barcelona Hospital Campus, Passeig Vall d’Hebron 119–129, 08035 Barcelona, Spain; Centro de Investigación Biomédica en Red de Enfermedades Hepáticas y Digestivas (CIBEREHD), Instituto de Salud Carlos III, Av. Monforte de Lemos, 3–5, 28029 Madrid, Spain; Biochemistry and Molecular Biology Department, Universitat Autònoma de Barcelona (UAB), Campus de la UAB, Plaça Cívica, 08193 Bellaterra, Spain; Liver Diseases-Viral Hepatitis, Liver Unit, Vall d’Hebron Institute of Research (VHIR), Instituto de Investigación Sanitaria Hospital Universitari Vall d’Hebron (IIS IR-HUVH), Vall d’Hebron Barcelona Hospital Campus, Passeig Vall d’Hebron 119–129, 08035 Barcelona, Spain; Centro de Investigación Biomédica en Red de Enfermedades Hepáticas y Digestivas (CIBEREHD), Instituto de Salud Carlos III, Av. Monforte de Lemos, 3–5, 28029 Madrid, Spain; Medicine Department, Universitat Autònoma de Barcelona (UAB), Campus de la UAB, Plaça Cívica, 08193 Bellaterra, Spain; Centro de Investigación Biomédica en Red de Enfermedades Hepáticas y Digestivas (CIBEREHD), Instituto de Salud Carlos III, Av. Monforte de Lemos, 3–5, 28029 Madrid, Spain; Department of Radiation Oncology, Vontz Center - 2434 Lab,University of Cincinnati College of Medicine, 3125 Eden Ave, Cincinnati, OH 45267, United States; Centro de Investigación Biomédica en Red de Enfermedades Hepáticas y Digestivas (CIBEREHD), Instituto de Salud Carlos III, Av. Monforte de Lemos, 3–5, 28029 Madrid, Spain; Liver Unit, Microbiology Department, Vall d’Hebron Institute of Research (VHIR), Instituto de Investigación Sanitaria Hospital Universitari Vall d’Hebron (IIS IR-HUVH), Passeig Vall d’Hebron 119–129, 08035 Barcelona, Spain; Liver Diseases-Viral Hepatitis, Liver Unit, Vall d’Hebron Institute of Research (VHIR), Instituto de Investigación Sanitaria Hospital Universitari Vall d’Hebron (IIS IR-HUVH), Vall d’Hebron Barcelona Hospital Campus, Passeig Vall d’Hebron 119–129, 08035 Barcelona, Spain; Centro de Investigación Biomédica en Red de Enfermedades Hepáticas y Digestivas (CIBEREHD), Instituto de Salud Carlos III, Av. Monforte de Lemos, 3–5, 28029 Madrid, Spain; Liver Unit, Microbiology Department, Vall d’Hebron Institute of Research (VHIR), Instituto de Investigación Sanitaria Hospital Universitari Vall d’Hebron (IIS IR-HUVH), Passeig Vall d’Hebron 119–129, 08035 Barcelona, Spain; Liver Diseases-Viral Hepatitis, Liver Unit, Vall d’Hebron Institute of Research (VHIR), Instituto de Investigación Sanitaria Hospital Universitari Vall d’Hebron (IIS IR-HUVH), Vall d’Hebron Barcelona Hospital Campus, Passeig Vall d’Hebron 119–129, 08035 Barcelona, Spain; Centro de Investigación Biomédica en Red de Enfermedades Hepáticas y Digestivas (CIBEREHD), Instituto de Salud Carlos III, Av. Monforte de Lemos, 3–5, 28029 Madrid, Spain; Medicine Department, Universitat Autònoma de Barcelona (UAB), Campus de la UAB, Plaça Cívica, 08193 Bellaterra, Spain; Liver Diseases-Viral Hepatitis, Liver Unit, Vall d’Hebron Institute of Research (VHIR), Instituto de Investigación Sanitaria Hospital Universitari Vall d’Hebron (IIS IR-HUVH), Vall d’Hebron Barcelona Hospital Campus, Passeig Vall d’Hebron 119–129, 08035 Barcelona, Spain; Centro de Investigación Biomédica en Red de Enfermedades Hepáticas y Digestivas (CIBEREHD), Instituto de Salud Carlos III, Av. Monforte de Lemos, 3–5, 28029 Madrid, Spain; Medicine Department, Universitat Autònoma de Barcelona (UAB), Campus de la UAB, Plaça Cívica, 08193 Bellaterra, Spain; Centro de Investigación Biomédica en Red de Enfermedades Hepáticas y Digestivas (CIBEREHD), Instituto de Salud Carlos III, Av. Monforte de Lemos, 3–5, 28029 Madrid, Spain; Liver Unit, Microbiology Department, Vall d’Hebron Institute of Research (VHIR), Instituto de Investigación Sanitaria Hospital Universitari Vall d’Hebron (IIS IR-HUVH), Passeig Vall d’Hebron 119–129, 08035 Barcelona, Spain; Respiratory Viruses Unit, Virology Section, Microbiology Department, Vall d’Hebron Institute of Research (VHIR), Instituto de Investigación Sanitaria Hospital Universitari Vall d’Hebron (IIS IR-HUVH), Passeig Vall d’Hebron 119–129, 08035 Barcelona, Spain; Biochemistry Department, Vall d’Hebron Institute of Research (VHIR), Instituto de Investigación Sanitaria Hospital Universitari Vall d’Hebron (IIS IR-HUVH), Passeig Vall d’Hebron 119–129, 08035 Barcelona, Spain; Clinical Biochemistry, Drug Delivery and Therapy (CB-DDT) Research Group, Vall d’Hebron Institute of Research (VHIR), Instituto de Investigación Sanitaria Hospital Universitari Vall d’Hebron (IIS IR-HUVH), Passeig Vall d’Hebron 119–129, 08035 Barcelona, Spain; Biochemistry and Molecular Biology Department, Universitat Autònoma de Barcelona (UAB), Campus de la UAB, Plaça Cívica, 08193 Bellaterra, Spain; Respiratory Viruses Unit, Virology Section, Microbiology Department, Vall d’Hebron Institute of Research (VHIR), Instituto de Investigación Sanitaria Hospital Universitari Vall d’Hebron (IIS IR-HUVH), Passeig Vall d’Hebron 119–129, 08035 Barcelona, Spain; Centro de Investigación Biomédica en Red de Enfermedades Infecciosas (CIBERINFEC), Instituto de Salud Carlos III, Av. Monforte de Lemos, 3–5, 28029 Madrid, Spain; Biochemistry and Molecular Biology Department, Universitat Autònoma de Barcelona (UAB), Campus de la UAB, Plaça Cívica, 08193 Bellaterra, Spain; Respiratory Viruses Unit, Virology Section, Microbiology Department, Vall d’Hebron Institute of Research (VHIR), Instituto de Investigación Sanitaria Hospital Universitari Vall d’Hebron (IIS IR-HUVH), Passeig Vall d’Hebron 119–129, 08035 Barcelona, Spain; Biochemistry and Molecular Biology Department, Universitat Autònoma de Barcelona (UAB), Campus de la UAB, Plaça Cívica, 08193 Bellaterra, Spain; Respiratory Viruses Unit, Virology Section, Microbiology Department, Vall d’Hebron Institute of Research (VHIR), Instituto de Investigación Sanitaria Hospital Universitari Vall d’Hebron (IIS IR-HUVH), Passeig Vall d’Hebron 119–129, 08035 Barcelona, Spain; Biochemistry and Molecular Biology Department, Universitat Autònoma de Barcelona (UAB), Campus de la UAB, Plaça Cívica, 08193 Bellaterra, Spain; Respiratory Viruses Unit, Virology Section, Microbiology Department, Vall d’Hebron Institute of Research (VHIR), Instituto de Investigación Sanitaria Hospital Universitari Vall d’Hebron (IIS IR-HUVH), Passeig Vall d’Hebron 119–129, 08035 Barcelona, Spain; Centro de Investigación Biomédica en Red de Enfermedades Infecciosas (CIBERINFEC), Instituto de Salud Carlos III, Av. Monforte de Lemos, 3–5, 28029 Madrid, Spain; Centro de Investigación Biomédica en Red de Enfermedades Hepáticas y Digestivas (CIBEREHD), Instituto de Salud Carlos III, Av. Monforte de Lemos, 3–5, 28029 Madrid, Spain; Basic Science Department, International University of Catalonia, Sant Cugat del Vallès, 08195 Barcelona, Spain; Liver Diseases-Viral Hepatitis, Liver Unit, Vall d’Hebron Institute of Research (VHIR), Instituto de Investigación Sanitaria Hospital Universitari Vall d’Hebron (IIS IR-HUVH), Vall d’Hebron Barcelona Hospital Campus, Passeig Vall d’Hebron 119–129, 08035 Barcelona, Spain; Centro de Investigación Biomédica en Red de Enfermedades Hepáticas y Digestivas (CIBEREHD), Instituto de Salud Carlos III, Av. Monforte de Lemos, 3–5, 28029 Madrid, Spain; Biochemistry and Molecular Biology Department, Universitat Autònoma de Barcelona (UAB), Campus de la UAB, Plaça Cívica, 08193 Bellaterra, Spain; Medicine Department, Universitat Autònoma de Barcelona (UAB), Campus de la UAB, Plaça Cívica, 08193 Bellaterra, Spain

**Keywords:** viral quasispecies, quasispecies structure, diversity, evenness, maturity score, haplotype synonymy, antiviral resistance

## Abstract

Previous studies based on clinical data from hepatitis C virus and hepatitis E virus infections revealed a deterministic evolution of quasispecies structure, irrespective of haplotype identities, towards a flat-like landscape, characterized by the absence of dominance and high evenness, combined with high haplotype synonymy. Here, two idealized limiting quasispecies states, A and Z, are defined, and it is shown that the A-to-Z evolution describes a parabolic trajectory between these states. The initial phase is dominated by increasing genetic diversity, whereas the subsequent phase is driven primarily by increasing evenness in the haplotype distribution. This evolutionary progression confers a broad domain within the genetic space, resulting in increased fitness and resilience, accompanied by a diminished response to antiviral therapies and multiple escape routes. Finally, a normalized quasispecies maturity score is proposed to position a given quasispecies along this structural evolutionary trajectory. This conceptual framework helps to account for the challenges in treating advanced chronic infections in which therapeutic failure frequently occurs in the absence of resistance-associated mutations.

## Introduction and background

A quasispecies is the complex and dynamic population of closely related but genetically diverse viral variants that arises during RNA virus infection. This population structure arises due to the high mutation rates of RNA viruses, which can introduce multiple errors during genome replication. The quasispecies consists of a mutant spectrum or cloud of viral genomes that are continuously generated and subject to selection within the host (Domingo et al. 2012, Domingo and Perales 2019, Domingo et al. 2021). A quasispecies observation consists in sequencing to high depth a quasispecies sample taken from a biosample infected with an RNA virus, and it is represented by a set of genomes (haplotypes) and observed frequencies, in the form of next-generation sequencing (NGS) read counts (Gregori et al. 2025).

Recent studies on samples from patients chronically infected with hepatitis E virus (HEV) who failed antiviral treatment with ribavirin, a drug with mutagenic effects, have revealed unprecedented levels of genetic diversity. This exceptionally high diversity, once thought to precede viral extinction, has instead been shown to remain highly functional, supporting persistent high viremia due to extensive haplotype synonymy among a limited number of functional phenotypes (Gregori et al. 2022, 2024a, Colomer-Castell et al. 2023). Both properties are characteristic of flat-like quasispecies. A flat-like quasispecies is an RNA virus population composed of a very high number of haplotypes with no clearly dominant genome, such that many variants coexist at relatively similar frequencies and occupy a broad region of the functional genetic space of the virus. Conceptually, it corresponds to a population located on a low but broad (‘flat’) region of the fitness landscape, with high average fitness and strong mutational robustness, so that additional mutations have little effect on overall fitness and the population can move through sequence space while maintaining viability (Wilke et al. 2001, Wilke 2005, Sanjuán et al. 2007, Sardanyés et al. 2008, Lauring and Andino 2010, Lauring et al. 2013, Gregori et al. 2024a, 2024b).

Similarly, a significant fraction of chronically hepatitis C virus (HCV)–infected patients who fail direct-acting antiviral (DAA) therapy show no resistance mutations at appreciable frequencies, particularly among those with advanced liver damage, reflecting long-term infection (Chen et al. 2020, Soria et al. 2020, Aldunate et al. 2021, Sarrazin 2021, Mushtaq et al. 2022, Dietz et al. 2023, Inzaule et al. 2024, Premkumar et al. 2025). In a previous study (Gregori et al. 2024b), we demonstrated that higher fibrosis stages are associated with greater genetic diversity and quasispecies structures characterized by low dominance and high haplotype distribution evenness. These treatment failures cannot be fully explained by the emergence of classical resistance mutations. Moreover, the resistance-associated variant (RAV)–centric view of resistance as a phenomenon driven by single point mutations appears overly simplistic (Soria et al. 2020). Instead, multiple substitutions, not directly annotated as resistance mutations, may exert a combined effect that alters the targeted protein’s configuration and geometry in the vicinity of the binding site, thereby reducing drug affinity and contributing to treatment failure.

*In vitro* studies with HCV have reported that highly diversified quasispecies, exhibiting increased fitness, show reduced response to different antiviral treatments, failing at doses proven effective against less evolved quasispecies of the same genotype (Perales et al. 2013, Sheldon et al. 2014, Gallego et al. 2016, 2018, Domingo et al. 2020).

We focused on the genetic structure of the quasispecies, independent of specific haplotype identities, as a key factor in reduced treatment response and failure (Gregori et al. 2022, 2024a, 2024b, 2025). The haplotype distribution within a quasispecies reflects the cumulative outcomes of its infection history. The quasispecies acts as a reservoir, preserving and promoting variants generated by replication errors that remain functional and possess substantial replication capacity or fitness, while poorly adapted variants are outcompeted due to their weak replication ability. Short-lived infections exhibit low genetic diversity, with strong dominance by one or few haplotypes, whereas long-lasting chronic infections display high genetic diversity, absence of dominance, and high evenness, supported by high haplotype synonymy among a few fit phenotypes that preserves functionality. This structural evolutionary process of progressive genetic diversification and enrichment with alternatively fit haplotypes is termed quasispecies maturation (Gregori et al. 2024b). A mature quasispecies occupies a broad domain in genetic space, offering multiple escape routes against external challenges and exhibiting high fitness with pronounced resilience to antiviral treatments.

In this article, we define two idealized limiting quasispecies structural states: an initial state characterized by high dominance and a state representing the highest possible genetic diversity. Any given quasispecies will lie between these two structural extremes, with more mature quasispecies positioned closer to the latter state. A concise set of structural indicators is used and discussed to describe the key features of the haplotype distribution, demonstrating its robustness and applicability across diverse viral populations.

The data presented in the Results section, which support this described behaviour, derive from a cohort of 69 chronically HCV-infected patients with available fibrosis data who failed DAA treatment. The dataset includes 263 amplicons covering the NS3, NS5A, and NS5B genomic regions, capturing a wide range of intra-host genetic diversity. In addition, samples from various RNA respiratory viruses, typically associated with short-lived acute infections, and from HEV-infected patients who failed ribavirin treatment and displayed extremely high genetic diversity, serve to bracket the observed spectrum of quasispecies structures, from low-diversity, acutely replicating populations to highly diverse, mature chronic infections. Moreover, these diverse viruses suggest that the same structural evolution can be expected from any RNA virus that exists as a quasispecies. The observed behaviour in these clinical samples is reproduced in an *in silico* study using simulated rank–abundance haplotype distributions that span the full spectrum of diversities. This *in silico* study is presented in the Supplementary material, together with a statistical analysis of the HCV cohort and practical recommendations for implementing the proposed framework.

## Material and methods

### Patients and samples

In a previous study, we demonstrated the association of liver damage to quasispecies maturity (Gregori et al. 2024b). To extend this finding, we deeply sequenced samples from a cohort of 69 chronically HCV-infected patients with fibrosis data available (4 F1, 6 F2, 18 F3, 41 F4) across diverse subtypes (22 1a, 19 1b, 1 1l, 20 3a, 1 4a, and 6 4d), using amplicons covering the genomic regions of interest for resistance-associated substitution (RAS) detection (1 of NS3, 1 of NS5A, and 2 of NS5B; Table 1). This yielded 263 amplicons.

**Table 1:** Amplicons sequenced for each virus in the study

Virus	*N*	Gene	FW.pos	RV.pos	AmplLen	GenBank
HCV	67	NS3	3513	3956	444	AF054249
HCV	68	NS5A	6327	6713	387	AF054249
HCV	64	NS5B-1	7971	8366	396	AF054249
HCV	64	NS5B-2	8166	8561	396	AF054249
CoV-HKU1	1	S	23495	23900	354	MH940245.1
CoV-OC43	1	S	24973	25359	336	PP187318.1
RSV-A	1	S	5924	6306	335	PP151405.1
RSV-B	1	S	6415	6807	339	PP135061.1
SARS-CoV-2	1	S	23347	23713	315	MN908947.3
HEV	5	ORF2	6373	6784	363	LC770331.1

*N*, number of samples; FW.pos, position after the primer on the gene at 5′; RV.pos, position before the primer on the gene at 3′; AmplLen, amplicon length with primers trimmed. See Acronyms and symbols at the end of the manuscript.

Additionally, we selected five samples from five patients with acute infections caused by different respiratory viruses (CoV-HKU1, CoV-OC43, RSV-A, RSV-B, and SARS-CoV-2) and five samples from two patients with chronic HEV infections who experienced repeated treatment failures with ribavirin (Table 1). For respiratory viruses, we sequenced one amplicon of the spike (S) gene; for HEV, one amplicon of open read frame 2 (ORF2) (Table 1). The respiratory virus samples represent typical acute infection quasispecies with highly dominant haplotypes (Gregori et al. 2024a). The HEV samples, with exceptionally high diversity, prompted the use of several metrics we denote as quasispecies fitness fractions (QFFs, described in the methods to follow) (Gregori et al. 2022, Colomer-Castell et al. 2023) as a numeric and visual tool to explore the relationship between genetic diversity and phenotypic functionality, while also illustrating the limitations of mutagenic monotherapy (Colomer-Castell et al. 2023) and exemplifying flat-like in-host quasispecies (Gregori et al. 2024a).

These non-HCV samples serve as bracketing quasispecies, encompassing respiratory virus infections with diversity substantially lower than that of HCV samples and highly mutagenized yet functional HEV quasispecies defining the upper extreme of observed diversity.

### Sample preparation

Viral RNAs were extracted from 140 $\mathrm{\mu} \mathrm{l}$ of sample (serum or nasopharyngeal swab) by manual extraction using the QIAmp Viral RNA Mini Kit (Qiagen, Hilden, Germany) following the manufacturers’protocol, but no RNA carrier was added during sample lysing. RNA was collected in 30 $\mathrm{\mu} \mathrm{l}$ of elution buffer. For each region, reverse transcription at 50°C for 30 min and a first cDNA amplification by polymerase chain reaction (PCR) of 35 cycles at 55°C were performed using the OneStep RT-PCR Transcriptor Kit (Roche Applied Science, Basel, Switzerland). PCR products were analysed by electrophoresis on $1.5\%$ agarose gel (Agarose MP; Roche Indianapolis, IN, USA), and the expected size-specific DNA bands were extracted from the gel and purified using the QIAquick Gel Extraction Kit (Qiagen, Valencia, CA, USA). Amplified and purified DNA was quantified by fluorescence using the Quant-iT Qubit dsDNA BR Assay Kit (ThermoFisher Scientific, Waltham, MA, USA). All amplicons were normalized to the same concentration and purified using KAPA Pure Beads magnetic beads (KAPA Biosystems; Roche Applied Science, Pleasanton, CA, USA). A second quantification by fluorescence was performed using the Quant-iT Qubit dsDNA HS Assay Kit (ThermoFisher Scientific), followed by a second normalization of all DNA pools to 1.5 ng/$\mathrm{\mu} \mathrm{l}$. Library preparation for MiSeq used the KAPA HyperPrep Kit (Roche Applied Science) standardized protocol with the SeqCap Adapters A/B (Nimblegen; Roche Applied Science) to mark each sample with a specific index. A second cleanup with KAPA Pure Beads (KAPA Biosystems; Roche Applied Science) was performed to remove small DNA fragments that could contaminate the samples. Quality testing was performed with the Agilent DNA 1000 kit and bioanalyzer (Santa Clara, CA, USA) or 4200 TapeStation System (Agilent). Lastly, normalization of all samples at 4 nM before mixing 10 $\mathrm{\mu} \mathrm{l}$ of each to the final library tube were performed. The final library was quantified by qPCR using the KAPA Library Quantification Kit (KAPA Biosystems; Roche Applied Science) with a LightCycler480 (Roche) to obtain exactly the concentration of indexed DNA. Last dilution and mix with an internal DNA Control (PhiX V3; Illumina, San Diego, CA) were performed before loading the library to the MiSeq Reagent Kit 600 V3 cartridge (Illumina) and sequenced using the MiSeq platform (Illumina). The flow cell was loaded with 600 $\mathrm{\mu} \mathrm{l}$ of the library (containing PhiX) at 20 pM of denatured DNA final concentration, yielding $7.2\times{10}^9$ single-strand molecules.

### Deep sequencing

To achieve a comprehensive picture of quasispecies composition, our previous results have underlined the need to perform deep-sequencing studies with high coverage (over $1.0\times{10}^4$ reads per amplicon) and avoid unnecessary abundance-based filter (Gregori et al. 2018, 2022, 2024b, Colomer-Castell et al. 2023). This is particularly important with mutagenic treatments, where their effect in the short term is mainly observed at the lowest level of haplotype frequencies (Gregori et al. 2018, 2022). Accordingly, deep sequencing was carried out using Illumina MiSeq instruments, yielding coverages ranging from 6K to 141K reads per strand, amplicon, and sample in the HCV dataset (Q1: 31K; Q2: 43K; Q3: 58K). These coverage levels are typical in clinical practice for reporting potential resistance-associated mutations. Respiratory virus and HEV samples were sequenced at substantially higher depths, achieving coverages of 263–368K for respiratory viruses and 265–304K for HEV, both sequenced at comparable depth levels.

### Data treatment of Fastq files to amplicon haplotypes

The aim of the sequencing data treatment was to discard error-bearing reads while preserving full-length read integrity, so that haplotypes that completely cover the amplicon with their respective frequencies were incorporated. The steps in this process are as follows:

- Obtain Fastq files with Illumina (R) 2 × 300 bp paired-end reads;
- Recover full amplicon reads with FLASH (Magoc and Salzberg 2011) (minimum 20 bp overlap, maximum $10\%$ mismatches). The paired-end reads, when overlapped, result in reads covering the complete amplicon.
- Remove full reads with 5% or more bases below a Phred score of Q30, representing a 99.9% accuracy.
- Demultiplex and trim primers (max three differences accepted), identify strand.
- Collapse reads (molecules) to haplotypes (amplicon-genomes) and their frequencies as read counts.

The paired-end reads are overlapped to recover the full amplicon length, followed by a Q30 quality filter, a strict quality control step. A set of haplotypes and frequencies is obtained for each strand and amplicon. These haplotypes and their frequencies are the basis for all further calculations. Only forward strand haplotypes and frequencies have been used in this study. Alternatively, the mean values of each indicator for the two strands may be taken.

Long-read sequencing technologies have made significant strides but still face challenges in quality and application scope. While circular consensus sequencing methods (Lou et al. 2013, Wenger et al. 2019), such as PacBio’s HiFi reads, improve accuracy through multiple passes of the same DNA molecule, raw error rates remain higher than short-read technologies, typically ranging from 5% to 15% depending on the platform and chemistry. Phred scores (a measure of base-calling accuracy) above Q30 (99.9% accuracy) are currently unattainable for single-pass long reads, as even error-corrected consensus reads from Oxford Nanopore Technologies reach ~95%–99% accuracy (Q13–Q20). Sequencing depth remains a critical constraint: long-read studies often achieve 100–1000× coverage, limiting reliable variant detection to frequencies below 1% in viral quasispecies studies. Quality and depth factors collectively constrain the widespread adoption of long-read technologies for high-resolution viral population studies requiring both depth and precision. Full-reads filters by Phred scores above Q30 are still unthinkable with these technologies (Garcia-Pedemonte et al. 2023).

### Software and statistics

All computations were performed with the R language and environment for statistical computing (version 4-3-3) (R Core Team 2024), running under Windows 11 x64, with in-house developed R scripts, using *Biostrings* (v 2.70.3) (Pages et al. 2012) and *tidyverse* (v 2.0.0) (Wickham et al. 2019) package collection and its dependencies. Plots were generated using the *ggplot2* (v 4.0.1) (Hadley Wickham 2016) and *gridExtra* (v 2.3) (Auguie and Antonov 2017) packages. The submitted paper was entirely generated with *RStudio* (2025.09.2 + 418), the Integrated Development Environment for R (Allaire 2025).

### Rank–abundance cumulative distribution

The rank–abundance distribution (RAD) is a tool commonly used to study different types of complex populations (Whittaker 1965, McGill et al. 2007, Saeedghalati et al. 2017). Within the quasispecies context, the RAD comprises the vector of haplotype frequencies sorted in descending order of abundance. The rank–abundance cumulative distribution (RACD) consists in the vector of sorted and cumulative frequencies plotted against the relative haplotype ranks.

**Figure 1:**
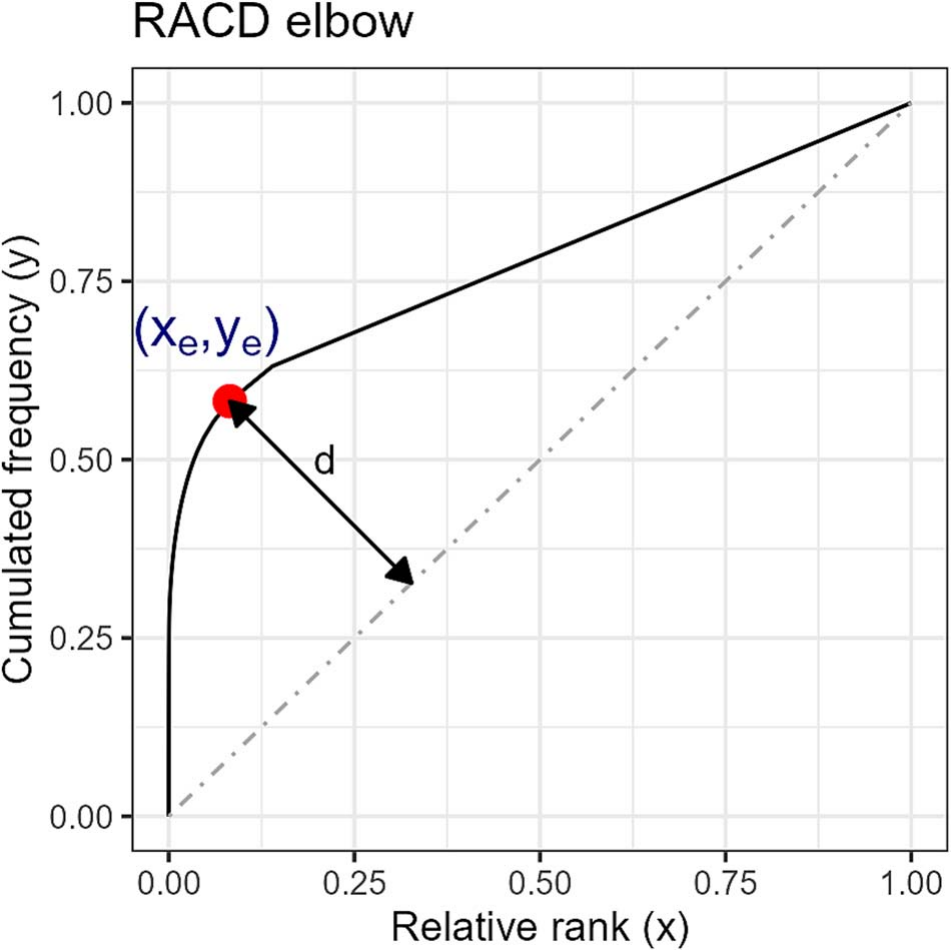
Rank–abundance cumulative haplotype distribution. Triangle threshold method to locate the curve elbow (dot).

Let ${x}_k$ be the relative rank of haplotype $k$ in a quasispecies with $H$ haplotypes, so that ${x}_k=k/H$, and let ${y}_k={\sum}_{i=1}^k{p}_i$ be the cumulative frequency up to the haplotype of rank $k$. The elbow on the RACD curve is defined as the point $\left({x}_e,{y}_e\right)$ that lies farthest from the straight line joining the first point of the RACD, (0,0), to the last point, (1,1), i.e. the point with the greatest perpendicular distance to this line (Fig. 1)


$$ e=\underset{k}{\mathrm{argmax}}\frac{\left|{y}_k-{x}_k\right|}{\sqrt{2}}=\underset{k}{\mathrm{argmax}}\left|{y}_k-{x}_k\right| $$

Alternatively, the elbow index $e$ may be computed as the first point on the curve where $\Delta y$ becomes less than or equal to $\Delta x$, with $\Delta x=1/H$ a fixed constant.


$$ e=\min \left\{k|\left({y}_{k+1}-{y}_k\right)\le \frac{1}{H}\right\} $$

Given the index $e$, the elbow corresponds to $\left({x}_e,{y}_e\right)$.

**Figure 2:**
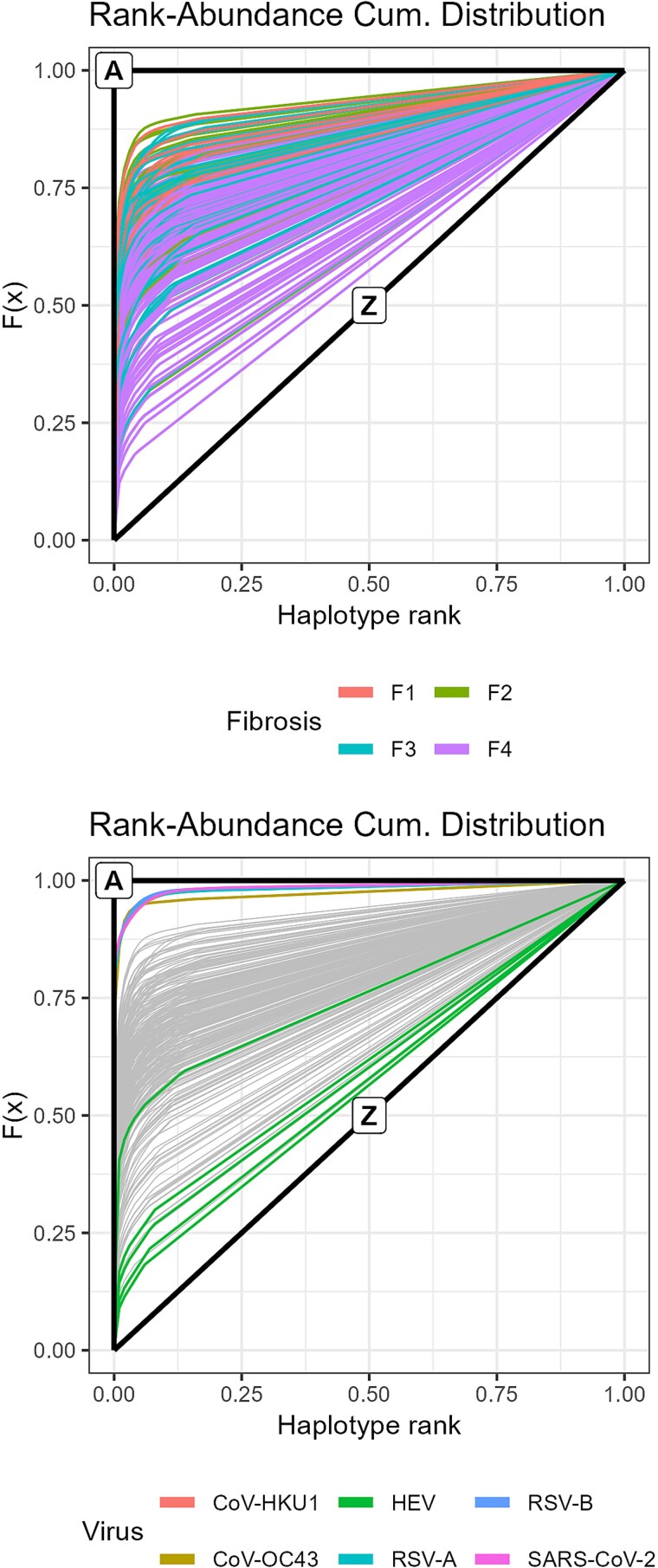
Rank–abundance cumulative distributions. Top: HCV quasispecies coloured by fibrosis stage. Bottom: bracketing quasispecies represented over greyed HCV data including idealized limiting states A and Z.

This point divides the curve in two sections:

- Steep part: The initial rapid increase corresponds to a few highly abundant haplotypes.
- Gradual part: Many rare species may add little to the total abundance beyond this point.

where the elbow is the inflection point, a noticeable ‘bend’ in the curve, and indicates where the contribution of new haplotypes to total abundance becomes less significant. The elbow reveals the presence of a fraction of ‘dominant’ haplotypes that may make up the bulk of the quasispecies, *versus* a ‘tail’ of many rare haplotypes (Fig. 2). A pronounced elbow suggests a quasispecies with a few common haplotypes and many rare ones (high dominance, low evenness). A gentler elbow (or a nearly straight line) indicates a more even distribution among all haplotypes (higher evenness). The location of the RACD elbow can be used to derive a general dominance score, represented by the cumulative frequency at the elbow (${y}_e$). Similarly, the normalized area over the RACD curve, AoC, can be used as maturity score, with


$$ \mathrm{AoC}=\frac{1}{0.5}\left[1-\frac{1}{H}{\sum}_{1=1}^H{y}_i\right] $$

where $\mathrm{AoC}(A)=0$ and $\mathrm{AoC}(Z)=1$.

### Relative logarithmic evenness profile

The relative logarithmic evenness (RLE) of order $q$, ${\mathrm{RLE}}_q$, is defined as the logarithm of the Hill number of order $q$, $D\left(P,q\right)$, normalized to the logarithm of the number of haplotypes in the quasispecies (Hill 1973, Jost 2010).


$$ \mathrm{RLE}q=\mathrm{RLE}\left(P,q\right)=\log \left(D\left(P,q\right)\right)/\log \left(D\left(P,0\right)\right) $$

$$ D\left(P,q\right)={\left({\sum}_{i=1}^H{p}_i^q\right)}^{1/\left(1-q\right)} $$

where $H$ is the number of haplotypes and $P=\left({p}_1,{p}_2,\dots, {p}_H\right)$ is the vector of haplotype frequencies.

The RLE profile is the representation of RLE at increasing values of $q$ starting from $q=0$. This is a decreasing curve with $1=\mathrm{RL}{\mathrm{E}}_0\ge \mathrm{RL}{\mathrm{E}}_1\ge \mathrm{RL}{\mathrm{E}}_3\ge \cdots \ge \mathrm{RL}{\mathrm{E}}_{\infty}\ge 0$. ${I}_3$ is defined as the normalized area under the RLE profile between $q=0$ and $q=3$, and provides a global and comprehensive measure of evenness since beyond *q* = 3 the changes are asymptotic and minimal:


\begin{align*} {I}_3&=\frac{1}{3\cdotp \log (H)}{\int}_{q=0}^{q=3}\log \left(D\left(P,q\right)\right) dq\\&=\frac{1}{3\cdotp \log (H)}{\int}_{q=0}^{q=3}\log \left[{\left({\sum}_{i=1}^H{p}_i^q\right)}^{1/\left(1-q\right)}\right] dq \end{align*}

This expression has no general analytic solution; however, it may be estimated numerically using the trapezoidal rule:


$$ {I}_3\approx \frac{1}{6}\cdotp \left(1+2\cdotp \mathrm{RL}{\mathrm{E}}_1+2\cdotp \mathrm{RL}{\mathrm{E}}_2+\mathrm{RL}{\mathrm{E}}_3\right) $$

Hill numbers *qD* are well defined for real *q* ∈ [0,∞), enabling continuous approximation of the evenness profile. *I*_3_ is obtained via trapezoidal integration of RLE(*q*) from *q* = 0 to *q* = 3. With finely spaced Hill numbers (Δ*q* = 0.1), the trapezoidal rule provides > 99.9% accurate approximation of the true integral, as verified numerically.


$$ {I}_3\approx \frac{1}{3}\cdotp \frac{\Delta q}{2}\cdotp \left(1+2{Y}_1+2{Y}_2+\dots +2{Y}_{N-1}+{Y}_N\right) $$

where the ${Y}_i$ are the RLE values computed at $q=i\cdotp \Delta q$, and $N\cdotp \Delta q=3$. It may be verified that ${I}_3(A)=0$ and ${I}_3(Z)=1$.

### Quasispecies structure indicators

In recent studies, a set of quasispecies structure indicators have been proposed to

- Characterize highly diverse functional quasispecies with flat-like profiles (Gregori et al. 2024a).
- Associate liver damage stage to quasispecies maturity stage (Gregori et al. 2024b).
- Associate mutagenic treatments *in vitro* to accelerated quasispecies maturation (Gregori et al. 2025).

From these indicators, a reduced set consisting in *Master*, *Rare1*, *Singl*, *RLE1*, *RLE2*, and *RLEinf* (Table2) are selected as alternative metrics to the mentioned *Ye* and *I3*. They complement one another in characterizing the distribution of haplotypes (Table 2) and can be directly computed from rank–abundance distributions (Whittaker 1965, McGill et al. 2007, Saeedghalati et al. 2017). They are classified in two groups: QFF and QsE.

- QFF: Quasispecies fitness fractions (Gregori et al. 2022). The fractions of reads corresponding to the master haplotype, the rare fraction (haplotypes below 1%), and to singletons, i.e. haplotypes represented by a single read.
- QsE: Quasispecies evenness, representing evenness over the full haplotype distribution. Selected RLE values are at *q* = 1, 2 and infinity (Gregori et al. 2024a).

**Table 2:** Quasispecies structure indicators and defined values per limiting quasispecies state

Feature	Type	Description	State A	State Z
Master	QFF	Dominant haplotype frequency	1	0
Rare1	QFF	Fraction of reads for haplotypes ≤1%	0	1
Singl	QFF	Fraction of reads for singletons	0	1
RLE1	QsE	Relative logarithmic evenness at *q* = 1	0	1
RLE2	QsE	Relative logarithmic evenness at *q* = 2	0	1
RLEinf	QsE	Relative logarithmic evenness at *q* = infinity	0	1

QFF, quasispecies fitness fractions; QsE, quasispecies evenness indicators.

Importantly, these indicators have well-defined values in the two idealized limiting states A and Z.

Table 2 lists a brief description of each of these indicators and defined values in the two idealized limiting quasispecies states, A with a master haplotype approaching an abundance of 100% and few variants at infinitesimal frequencies and Z with a very high number of haplotypes sharing similar infinitesimal abundances.

The zero for Master, in state Z, should be interpreted as an infinitesimal frequency ε (a positive real number, with ε < 1/*n* for any positive integer *n*), rather than strictly zero. Biochemically, this corresponds to one to a few (or several) molecules in the context of high viral titre. Similarly, the zeros for Rare1 and Singl in state A represent minor variants (other than the master haplotype) at infinitesimal frequencies. However, we represent ε formally as 0.

There is great flexibility in defining and selecting structural indicators, as shown in the Supplementary material, provided they have well-defined values in the two idealized limiting states.

### Correlation and principal component analysis

The preceding set of indicators is highly correlated, despite capturing different and complementary aspects of quasispecies structure. This correlation can be addressed using principal components analysis (PCA) (Jolliffe 2002, Jolliffe and Cadima 2016), which provides an accurate projection of a set of quasispecies, each characterized by its vector of indicator values, into a reduced two-dimensional space. The data matrix containing the structural indicators for all HCV quasispecies is first centred by subtracting the mean and scaled by the standard deviation of each indicator. The covariance matrix of the resulting standardized values is then decomposed into mutually orthonormal eigenvectors by diagonalization, whose corresponding eigenvalues represent the fraction of total variance explained by each principal component (Ayres 1962, Jolliffe 2002). Letting $\left\{{v}_k\right\}$ denote the eigenvectors, the coordinates in the PC1/PC2 plane of a quasispecies with a vector of centred and scaled indicator values ${x}_{\mathrm{cs}}$ are given by $\left({v}_1^t\cdotp{x}_{\mathrm{cs}},{v}_2^t\cdotp{x}_{\mathrm{cs}}\right)$ which correspond to a rotation of the original quasispecies indicator axes into the principal component space (Ayres 1962, Demidovich and Maron 1981).

A new quasispecies can be projected onto the same two-dimensional subspace by centring and scaling its vector of indicator values $y$ using the previously determined means and standard deviations for each indicator, yielding ${y}_{\mathrm{cs}}$. The projection is then obtained by applying the rotations defined by the eigenvectors, resulting in coordinates $\left({v}_1^t\cdotp{y}_{\mathrm{cs}},{v}_2^t\cdotp{y}_{\mathrm{cs}}\right)$.

## Results

Two representations of the genetic quasispecies structure are used: the RACD and the RLE profile. The former is characterized by a pronounced bend, or ‘elbow’, that separates dominant from rare haplotypes; the latter uses the area under the RLE curve as an indicator of distribution evenness. Dominance (low entropy) and evenness (high entropy) represent the opposing characteristics of the two limiting quasispecies states. From these representations, specific metrics are defined to position a quasispecies along its structural evolution trajectory, from low entropy and high dominance (state A), through increasing diversity, to high evenness (state Z). Additionally, a concise set of quasispecies structure indicators is used to characterize its maturation stage. These indicators exhibit interesting properties that enable the definition of a maturity score, with the different proposed scores compared below.

### Rank–abundance cumulative distribution and dominance

The level of haplotype dominance is reflected by the position of the elbow on the RACD curve. Figure 2 shows, on top, the haplotype distributions of the HCV quasispecies, coloured by fibrosis stage, including the two idealized limiting states A and Z. Higher fibrosis stages correspond to lower dominance. The bottom panel displays the distributions of the respiratory viruses and the HEV samples over the greyed HCV distributions. While acute infections exhibit high dominance, most HEV samples show a markedly flattened profile.

### Relative logarithmic evenness profiles and evenness

**Figure 3:**
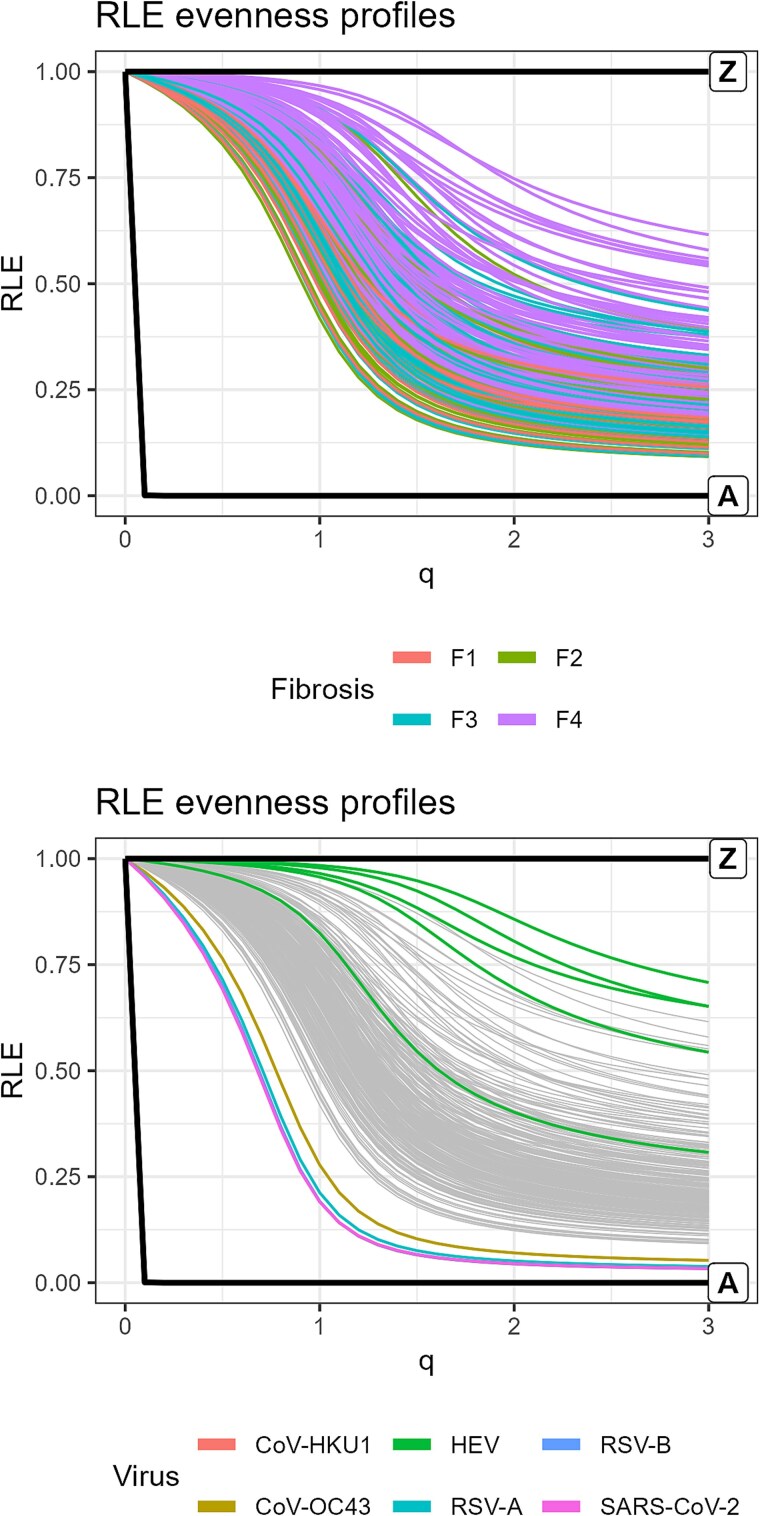
Relative logarithmic evenness profiles. Top: HCV quasispecies coloured by fibrosis stage. Bottom: bracketing quasispecies represented over greyed HCV data including idealized limiting states A and Z.

The height of the RLE profile indicates the evenness of haplotype distributions. Figure 3 displays, on top, the RLE profiles of the HCV quasispecies, including limiting states A and Z. Higher fibrosis stages are associated to higher profiles. The bottom panel shows the RLE profiles of respiratory viruses and HEV samples overlaid on the greyed HCV profiles. Acute infections exhibit very low evenness profiles, while most HEV samples show high profiles, reflecting advanced quasispecies maturity.

### Peaked-to-flat distribution: the natural evolutionary path

**Figure 4:**
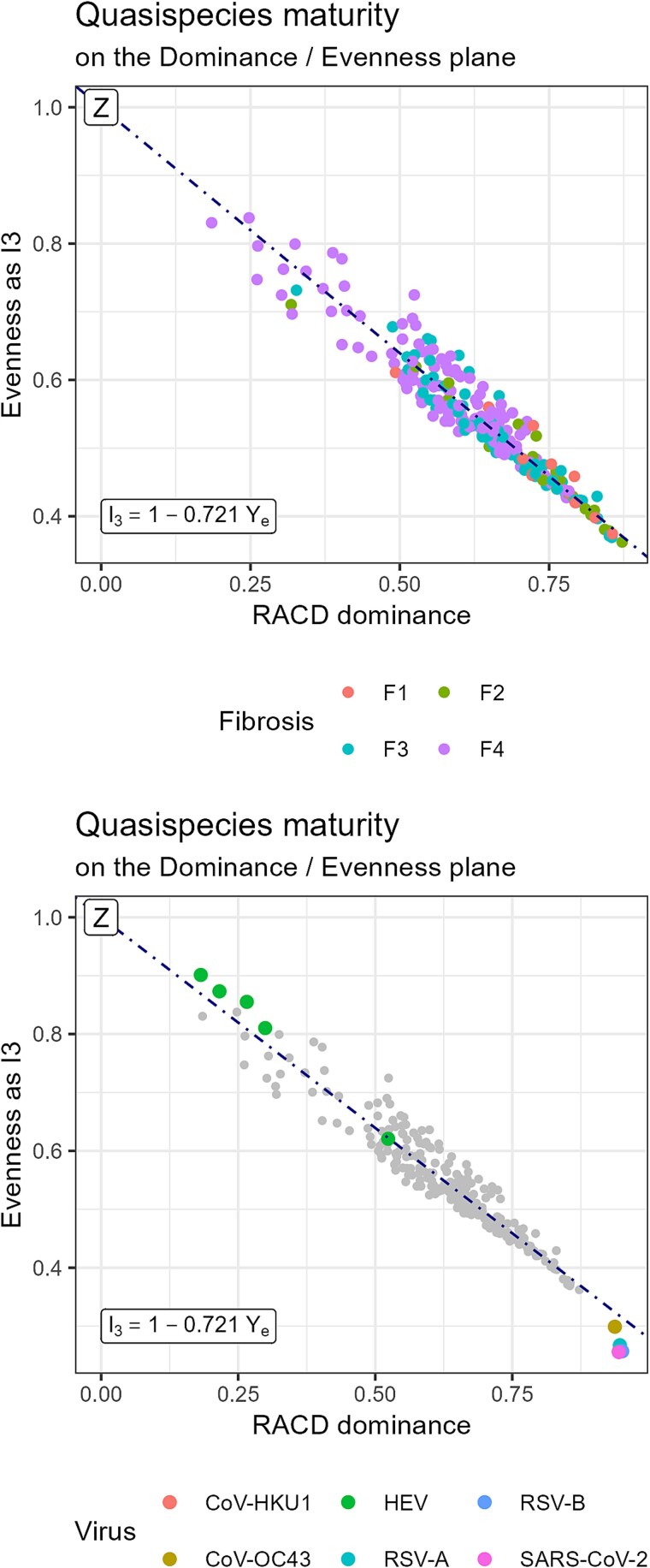
Dominance–evenness evolution axis. Top: HCV quasispecies coloured by fibrosis stage. Bottom: bracketing quasispecies represented over greyed HCV data including limiting state Z.

The evolution of high genetic dominance towards increased evenness and reduced dominance is shown in Fig. 4, which plots dominance scores—given by the cumulative frequency at the RACD curve elbow, ${Y}_{\mathrm{e}}$, against evenness scores, defined as the normalized area under the RLE profile between $q=0$ and $q=3$, ${I}_3$. Higher fibrosis stages exhibit reduced dominance and increased evenness. The lower panel of Fig. 4 displays the bracketing quasispecies overlaid on the greyed HCV quasispecies, clustering near the regression line fitted to the HCV data.

### Quasispecies structure indicators, correlation, and PCA

The selected quasispecies structure indicators relate directly to either dominance or evenness. *Master* serves as an alternative dominance indicator, while *Rare1* and *Singl* quantify the rare haplotype reads fractions; the ${{RLE}}_q$ indicators, in turn, represent evenness scores. Although these metrics capture distinct aspects of quasispecies structure, they exhibit strong correlations, as dominance and evenness provide complementary perspectives on the same underlying phenomenon. PCA (Jolliffe and Cadima 2016) is a linear transformation method that identifies the directions (principal components) along which the data vary the most.

**Figure 5:**
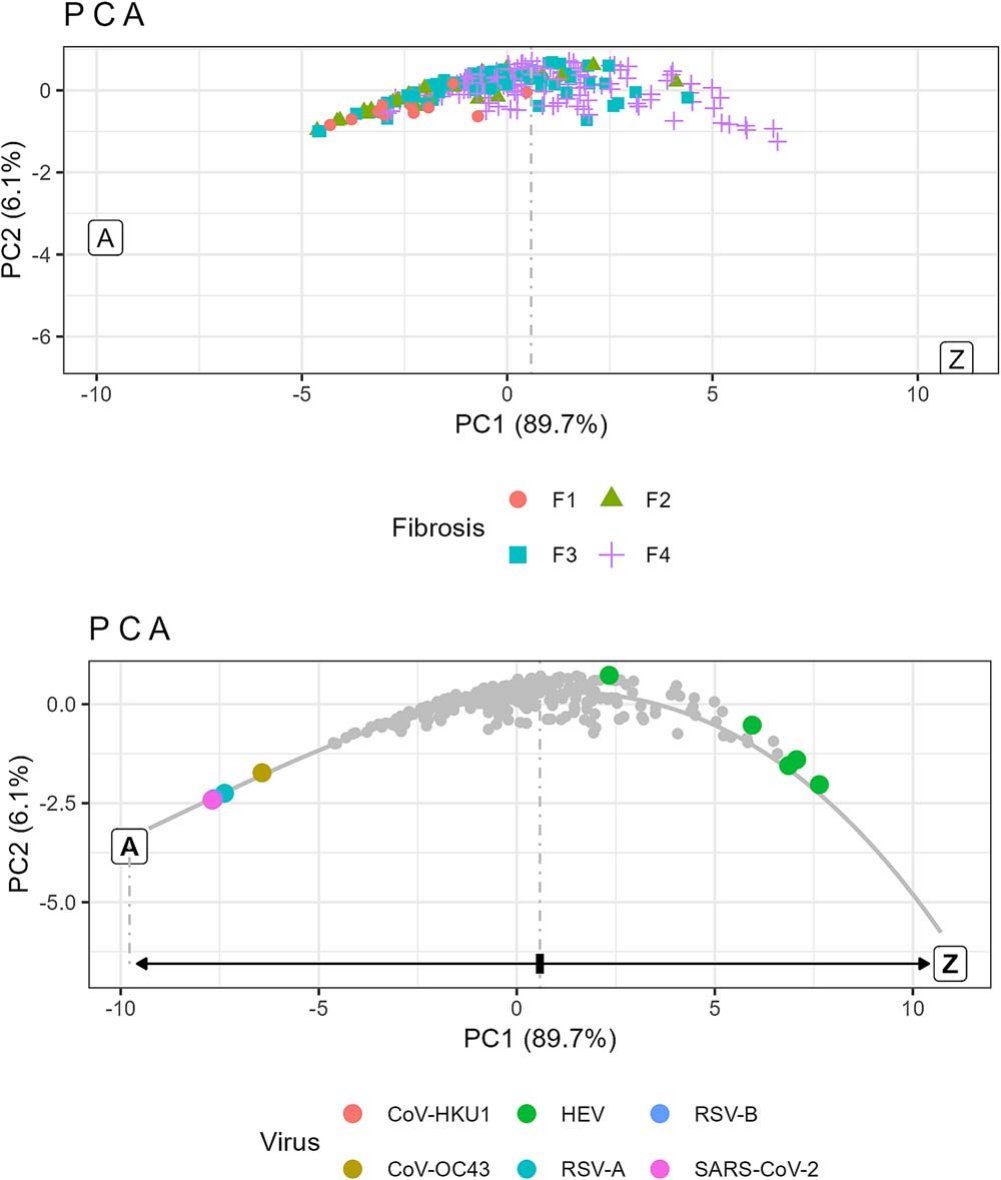
Projection of quasispecies on the PC1/PC2 plane, including limiting states A and Z, explaining 95.8% of the total variance of the dataset. Top: HCV quasispecies coloured by fibrosis stage. Bottom: Bracketing quasispecies shown over the greyed HCV data, including limiting states A and Z, with fitted cubic polynomial on the HCV dataset. Dash-dot line on curve apex.

The PCA of the matrix of these indicators for the HCV quasispecies yields a PC1 accounting for 89.7% of the variance in the full dataset and a PC2 explaining an additional 6.1%. Thus, the representation of these quasispecies on the PC1/PC2 plane captures 95.8% of the original variance of the full matrix of quasispecies indicators with high accuracy. Furthermore, the quasispecies cluster tightly around a cubic polynomial curve. A fitted cubic polynomial yields an adjusted ${R}^2$ of 0.9968 with high statistical significance, passing through the limiting states. Figure 5 illustrates the PCA representation: the top panel shows the HCV quasispecies, while the bottom panel displays the bracketing quasispecies projected onto this subspace, closely aligning to the fitted curve.

### Maturity scores

**Figure 6:**
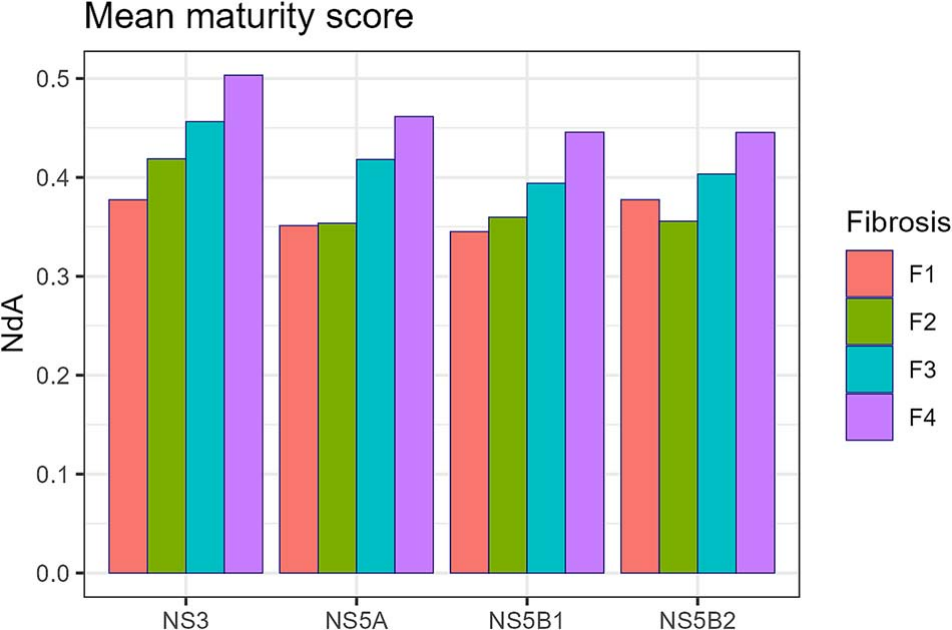
Mean normalized maturity scores as NdA by amplicon and fibrosis stage, computed as the arc-length on the PCA cubic polynomial from state A, normalized by the A-to-Z arc-length.

**Figure 7:**
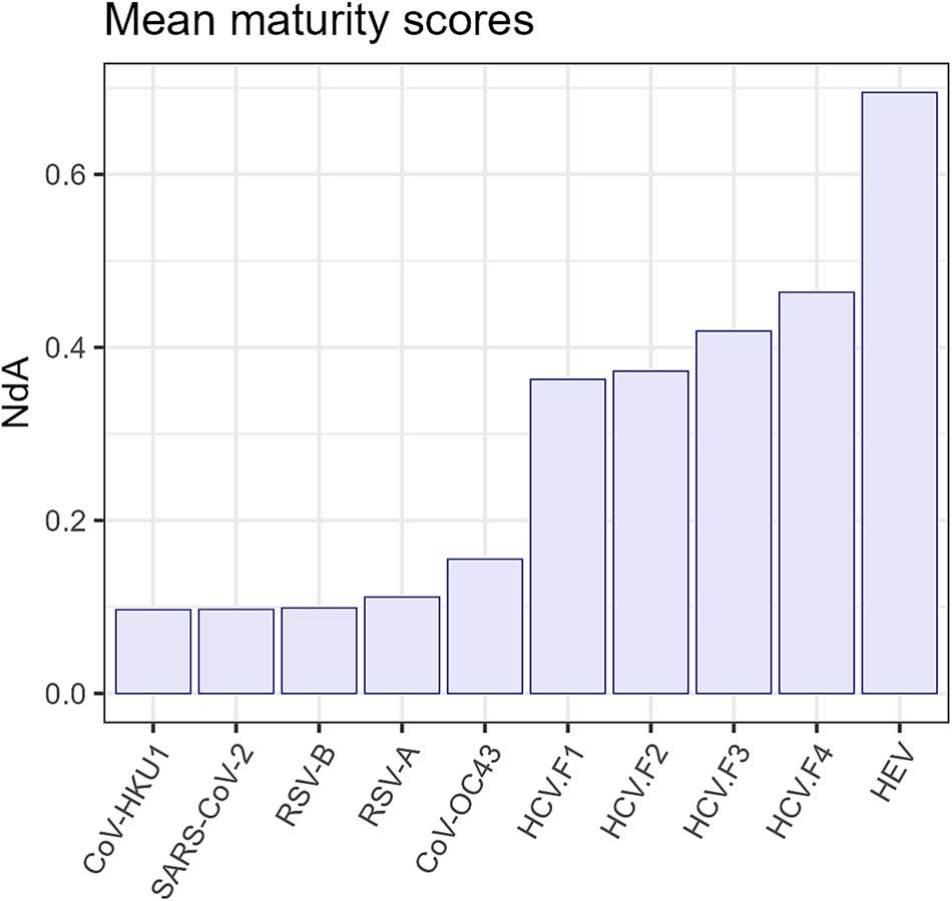
Mean normalized maturity scores as NdA by virus, computed as the arc-length on the fitted cubic polynomial curve on the PCA from state A, normalized by the A-to-Z arc-length.

Besides the proposed maturity scores ${Y}_e$ and $I3$, as shown in Fig. 4, we introduce a general maturity score $NdA$, defined as the arc-length along the fitted polynomial curve from state A to the quasispecies position in the PC1/PC2 plane, normalized relative to the total A-to-Z arc-length. Figure 6 displays the mean $NdA$ values for each genomic region and fibrosis stage in the HCV dataset. Figure 7 shows the mean $NdA$ values by virus type, including the different fibrosis stages; this later comparison is not intended to establish statistical differences—given the limited number of bracketing samples—but rather to illustrate general trends.

**Figure 8:**
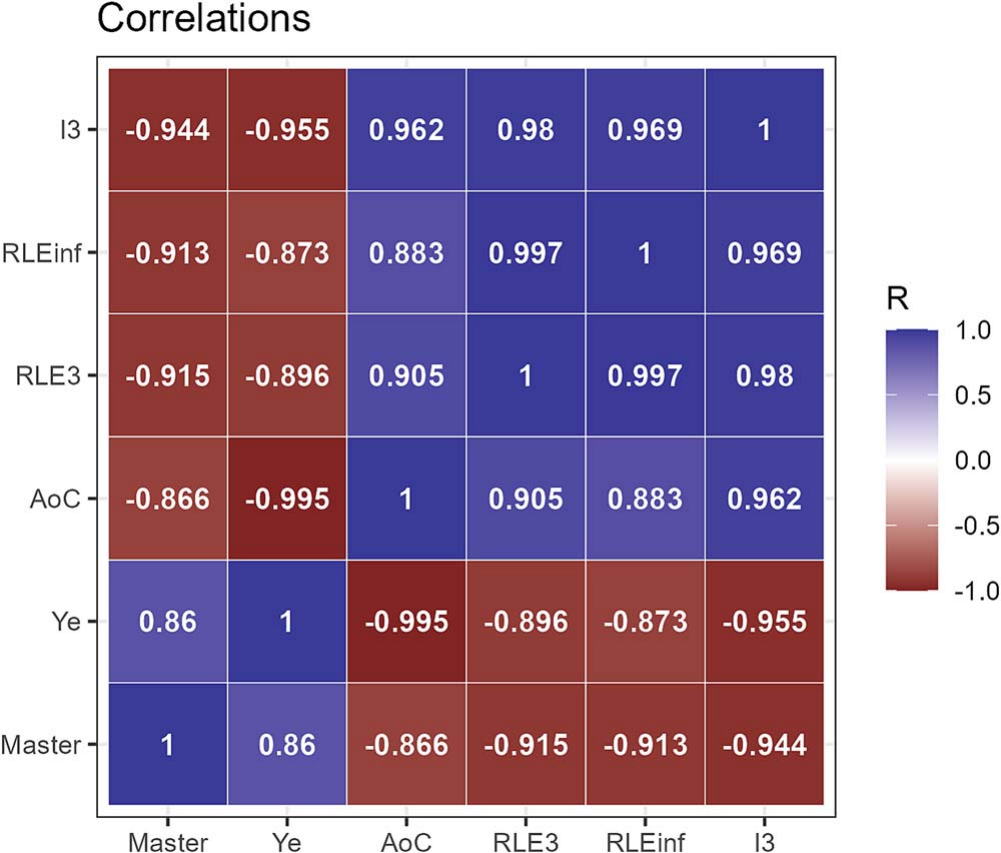
Correlations among the proposed scores to assess the maturity stage of a quasispecies in the full HCV dataset. These correlations are based on the 263 amplicons in the HCV cohort.

The set of proposed maturity metrics (*Ye*, *I3*, *NdA*) is highly correlated, as shown in Fig. 8, where they are also compared with simpler metrics such as *AoC*, the master haplotype frequency, *RLE3*, and *RLEinf*. High values of *Master* or *Ye* are indicative of a peaked low entropy distribution, whereas high values of *AoC*, *RLE3*, *RLEinf*, or *I3* indicate high evenness and maturity. These two groups are negatively correlated, with high absolute values of *R*.

Essentially, any of these (*Ye*, *I3*, *NdA*) may be used as a proxy for the overall replicative maturity of the quasispecies, depending on the specific experimental or analytical context. While *Ye* emphasizes the threshold between abundant and rare haplotypes, *I3* captures the evenness of the haplotype distribution, and NdA reflects the combined effect of quasispecies fitness fractions and distributional evenness. *NdA* is a smoothed score that depends on several indicators and may prove to be more robust than the other two. The Supplementary material includes a document with practical recommendations, offering options of graded difficulty and providing all necessary data, such as centring and scaling parameters, PCA loadings, and coefficients of the fitted polynomials.

### *In silico* study

The observed behaviour of these clinical samples was reproduced with an *in silico* study using quasispecies structures composed of 1000 haplotypes each, encompassing the full spectrum of diversities, from peaked to even distributions. Fifteen rank–abundance haplotype distributions were generated using Zipf–Pareto models [50–52], with α values ranging from 0.11 to 5, along with two bracketing quasispecies and two idealized limiting models (Figs. B1–B2). The same parabolic behaviour observed in the clinical samples, illustrating the A-to-Z evolutionary path, was also obtained **(**Figs. B3–B4**)**. These results are presented and discussed in the Supplementary material, which includes an appendix listing the R code used in the *in silico* study. Tables and Figures are prefixed with B in this document.

### Statistical analysis across fibrosis stage

A full statistical analysis of changes in an expanded set of quasispecies structure indicators across transitions between consecutive fibrosis stages is provided in the Supplementary material, including *P*-values, effect sizes, boxplots, and ROC curves. Tables and Figures are prefixed with S in this document. Unbalanced sample sizes (14 amplicons in F1, 23 in F2, 80 in F3, and 157 in F4; see Table S1) affect the results through their impact on statistical power. No significant changes were detected in the F1-to-F2 transition; however, the small number of F1 patients and amplicons (*n* = 4 and 14, respectively) results in low statistical power. Changes between F3 and F4 are highly statistically significant but exhibit lower effect sizes than those in the F2-to-F3 transition (Tables S3 and S4 and Figs. S6 and S7). The largest changes occur from F2 to F3, estimated to happen ~20–25 years post-infection (Thein et al. 2008) (Figs. S7, and  S8 to S12). Effect sizes (Fig. S7) suggest that diversification in early fibrosis stages is driven mainly by changes in QFF indicators, namely, decreasing top haplotype frequencies alongside increasing fractions of rare haplotype reads, whereas later diversification is primarily governed by increasing evenness in the quasispecies.

This statistical analysis included the study of haplotype synonymy, consistent with previous HEV and HCV quasispecies studies (Gregori et al. 2022, 2024a, 2024b). Notably, the association between maturity and synonymy scores exceeds their individual associations with fibrosis stage. Clinical confounders influencing quasispecies maturation exert parallel effects on haplotype synonymy.

The combined test results indicate that the F2-to-F3 transition is the key turning point in HCV quasispecies evolution, associated with a profound reshaping of the genetic structure. This transition occurs within the expected interval of 20 to 30 years post-infection (Thein et al. 2008).

### Implementation

A supplementary document discusses implementation details and offers options of graded difficulty, including all necessary data, such as centering and scaling parameters, PCA loadings, and coefficients of the fitted polynomials. R code snippets are included, complementing the R code in the appendix of the supplementary document containing the *in silico* study. The pseudocode describing the complete workflow, from raw FASTQ files to FASTA files with haplotypes and frequencies, is also provided to facilitate implementation of the entire data processing framework. Tables and Figures are prefixed with A in this document.

## Discussion

Viral evolution operates at two hierarchical levels: in-host and inter-host (Domingo and Perales 2019). A viral quasispecies represents an in-host evolutionary unit that is not fully transmissible, as transmission bottlenecks restrict the process, drastically reducing genetic diversity when only a subset of variants establishes infection in a new host. The present study addresses in-host evolution, defined as the temporal changes in composition, structure, and both genotypic and phenotypic diversity that a quasispecies experiences during infection, either naturally or under external perturbations such as antiviral therapy.

High-coverage NGS has greatly expanded our understanding of quasispecies complexity. Early analyses often applied stringent abundance filtering to minimize sequencing artefacts by excluding low-frequency haplotypes or reads not detected across complementary strands. Although this improved data reliability, it simultaneously removed potentially meaningful variants. Increasing evidence now shows that these low-abundance haplotypes often carry valuable information on quasispecies structure, evolutionary dynamics, and treatment response. Experimental data from HCV replication systems revealed continuous diversification driven by internal disequilibria and haplotype fitness gradients, even in the absence of external selective forces (Moreno et al. 2017, Domingo et al. 2020, Gallego et al. 2020). This diversification enhanced genetic diversity, improved quasispecies fitness, and promoted resistance to classically acting antivirals, DAAs, and mutagens (Perales et al. 2013, Sheldon et al. 2014, Gallego et al. 2016, 2018).

Further studies showed that the rare haplotype load (RHL), the fraction of haplotypes below 1% or 0.1% abundance, was a stronger marker of mutagenic pressure than traditional indices such as mutation frequency or nucleotide diversity (Gregori et al. 2018). These results underline limitations of classical diversity measures, which often fail to discriminate between distinct quasispecies states. Clinical observations have reinforced the importance of retaining low-frequency variants. In ribavirin-treated chronic HEV infections, extraordinarily high genetic diversity coexisted with preserved replication competence, even when most reads would have been excluded under strict abundance thresholds. Indicators such as haplotype synonymy and fitness fractions resolved this apparent paradox (Gregori et al. 2022, Colomer-Castell et al. 2023). Such findings prompted the introduction of new structural descriptors—quasispecies maturity, haplotype synonymy, and genetic amplitude—which have proven clinically relevant. In chronic HCV infection, for instance, higher maturity values correlate with more advanced liver disease (Gregori et al. 2024a).

Together, these findings support the existence of two limiting quasispecies states. State A represents a simple, short-lived structure typical of acute infections, whereas state Z corresponds to a highly diverse but functionally stable configuration found in chronic infections subjected to long-term replication or mutagenic pressure. These two idealized states, characterized by distinct combinations of structural indicators (Table 2), define a continuum from A to Z describing the deterministic progression of quasispecies structure. This trajectory reflects changes in the distribution profile of haplotypes rather than the persistence of specific variants, which remain unpredictable. In accordance with the concept of quasispecies as units of selection, long-term adaptation results from properties of the population as a whole, not from individual mutants.

Our results align with previous demonstrations that viral adaptation in cell culture proceeds through expansion of the mutant spectrum rather than convergence towards a new consensus sequence (Perales et al. 2013, Sheldon et al. 2014, Gallego et al. 2016, 2018, 2020, Moreno et al. 2017, Domingo et al. 2020). In addition, the concept of quasispecies as units of selection underscores how populations, rather than individual variants, drive long-term adaptation.

Mechanistically, this dynamic can be interpreted as progressive exploration of viral sequence space. Much of this space comprises synonymous substitutions or mutations with minimal functional cost. With each replication cycle, the quasispecies progressively occupies these low-cost regions, increasing haplotype richness and reducing dominance of the initial master haplotype. Over prolonged replication, numerous variants of comparable fitness accumulate, broadening the population’s repertoire of potential escape routes against immune or antiviral pressure. This mechanism explains how chronic infections sustain highly diverse yet functional populations and why persistence favours quasispecies with extensive adaptive potential.

In the present study, the stage of liver fibrosis, used as a proxy for infection duration, enabled investigation of quasispecies evolution even in the absence of detailed clinical timelines. Quasispecies from patients with advanced fibrosis displayed greater average diversity and maturity, in line with prolonged evolution. However other confounding factors in the patient’s clinic history impact the evolution state of quasispecies structure, as evidenced by the overlap of fibrosis populations (Figs. S8 to S12). High haplotype synonymy appeared to help preserve overall functionality despite extensive diversification. The inclusion of other viruses at different infection stages supports the view that this structural evolution model applies broadly across RNA viruses, albeit likely progressing at variable rates.

The statistical analysis of the HCV cohort, presented in the Supplementary material, reveals consistent mathematical relationships linking major descriptors of quasispecies structure. Particularly, top phenotype and top haplotype frequencies show coherent dependencies at low master haplotype frequencies (Fig. S14), and synonymy correlates with maturity following a double-exponential function (Fig. S17). Together, these relationships define a continuum of quasispecies states that project onto a polynomial curve along the PC1/PC2 plane, summarizing the main structural evolutionary axis.

Note, however, that although infection duration is highly correlated with fibrosis severity, multiple uncontrolled confounders—often not documented in clinical records—may influence quasispecies diversity and the progression of liver damage. These include

- The trajectory of viral load throughout infection, as persistently high viral loads imply accelerated accumulation of substitutions.
- Previous treatment history, including the type (mutagenic *versus* direct-acting antivirals), duration, and dosage. Mutagenic compounds tend to accelerate diversification, whereas DAAs promote the selection of subpopulations with reduced drug responsiveness and can transiently decrease quasispecies diversity.
- Targeted genomic regions: NS3/4A, NS5A, NS5B, or combinations thereof.
- Host factors: immune status, steatosis, co-infections, and metabolic syndrome.
- Time since treatment cessation or discontinuation, representing the quasispecies recovery period.

Notably, progression along the A–Z axis is unlikely to be linear or strictly monotonic (Fig. S11). Quasispecies evolution may include transient reversals caused by external perturbations such as immune activity or antiviral treatment. Such events can temporarily drive populations towards state A through selection of subpopulations with reduced antiviral sensitivity (a bottleneck effect). This behaviour has been observed in HCV replication studies in non-coevolving systems. There, quasispecies fitness increased by ~20% from passage 0 to 100, yielding populations resistant to multiple antiviral types for 10 passages at dosages that extinguished the p0 population within 4 to 5 passages (Perales et al. 2013, Sheldon et al. 2014, Gallego et al. 2016, 2018). Fitness gains paralleled rising genetic diversity, but the specific outcome depended on the drug type: mutagenic agents accelerated maturity relative to the p0–p100 progression, whereas the DAA sofosbuvir reduced both diversity and maturity, likely through enrichment of less susceptible haplotypes (Gregori et al. 2025). These shifts represent temporary regressions from state Z to state A, after which the quasispecies is expected to resume diversification and continued progression towards state Z once selective pressure is relaxed or removed.

The proposed maturity scores encapsulate the global evolutionary state of the quasispecies beyond that conveyed by fibrosis grade or infection duration. By integrating the cumulative influence of these covariates across each patient’s clinical history, they provide a composite measure of quasispecies maturity and, consequently, an indicator of its resilience to antiviral pressure.

Pending further validation, we propose that any RNA virus quasispecies will lie close to this polynomial curve in the PC1/PC2 plane. This hypothesis rests on three lines of evidence: (i) the strong correlations among quasispecies structure indicators, (ii) the characterization of two distinct limiting states with well defined values, and (iii) the constraint that all intermediate quasispecies configurations must fall between these two states. In agreement, all quasispecies examined—across disease stages and acute infections—clustered tightly around the predicted curve. Moreover, quasispecies from patients with advanced liver pathology displayed higher average maturity scores estimated along this trajectory. The observed unification across infections in ‘diversity space’ would benefit from further validation with additional datasets and infection types.

## Conclusions

This study integrates experimental, clinical, and statistical evidence to describe a unified model of in-host quasispecies structural evolution. Viral populations appear to follow a deterministic structural trajectory between two limiting states, from a simple configuration typical of acute infection (state A) towards a mature, highly diverse yet functionally stable structure (state Z). This trajectory, captured by a polynomial relationship among structural indicators, reflects population-level adaptation rather than the persistence of individual variants.

The proposed framework provides a quantitative language to describe quasispecies dynamics across RNA viruses, linking genetic structure with replication fitness and clinical outcomes. It also rationalizes how chronic infections maintain high diversity while preserving replication competence, and how transient perturbations, such as antiviral treatments, can momentarily reverse progression along this axis.

More broadly, these results position quasispecies structure as a central, measurable dimension of viral evolution. Extending this approach across different viruses and host contexts will help assess its generality, support predictive modelling of antiviral response, and refine our understanding of the balance between diversification and functional stability in persistent RNA virus populations.

## Inclusion and ethics

In this study, we have used leftover plasma or serum samples from diagnosis procedures of anonymized patients infected by one of the viruses described in the manuscript. The study was approved by Vall d’Hebron University Hospital Ethics Committee, with the reference numbers PR(AG)287-2022, PR(AG)118-2021, and PR(AG)259-2020.

## Acronyms and symbols

### Acronyms

CoV-HKU1 Human coronavirus HKU1

CoV-OV43 Human coronavirus OC43

DAA Direct-acting antiviral

e.a. Equally abundant

HCV Hepatitis C virus

HEV Hepatitis E virus

PC Principal component

PCA Principal components analysis

QFF Quasispecies fitness fraction

RAS Resistance-associated substitution

RAV Resistance-associated variant

RHL Rare haplotypes load

RLE Relative logarithmic evenness

RSV-A Respiratory syncytial virus A

RSV-B Respiratory syncytial virus B

RV Respiratory virus

SARS-CoV-2 Severe acute respiratory syndrome coronavirus 2

Symbols of quasispecies diversity, maturity and structure features.

### Symbols

AmplLen Amplicon length

$d2A$ Distance of a quasispecies from state A

$d2Z$ Distance of a quasispecies to state Z

$D\left(p,q\right)$ Hill number of order *q*

$D\left(p,0\right)$ Number of haplotypes

Hpl Haplotype

${I}_3$ Area under the RLE profile from $q=0$ to $q=3$

Master Most abundant haplotype frequency

$NdA$ Normalized distance from state A

Phn Phenotype

${p}_i$ *i*-th haplotype frequency

*q* Order of Hill numbers or RLE

Rare1 Fraction of reads for haplotypes $\le 1\%$

$\mathrm{RL}{\mathrm{E}}_q$ Relative logarithmic evenness of order *q*

TopN Top N haplotypes cumulated frequency

## Supplementary Material

Supplementary_material_veag024

## Data Availability

The data that support the findings of this study are openly available in the GenBank Sequence Read Archive database with BioProject accessions PRJNA1176499 (HCV), PRJNA1081281 (RV), PRJNA1195906 (HEV), and PRJNA876218 (HEV).
