# Supplementary material for: Peaked-to-flat transition in quasispecies structure evolution
Source: Virus Evol. 2026 Apr 14;12(1):veag024. doi: 10.1093/ve/veag024 (PMC13137331; doi:10.1093/ve/veag024)
Supplement: Supplementary_material_veag024 [file supplementary_material_veag024.zip › VEVOLU-2025-229_R2_Supplementary_1_In-Silico_veag024.pdf]

# Peaked-to-Flat Transition in Quasispecies Structure Evolution

Supplementary material: Simulated quasispecies structures

Josep Gregori* Sergi Colomer-Castell Carolina Campos Marta Ibañez-Lligoña
Damir García-Cehic Alvaro González-Camuesco Maria F. Cortese
David Tabernero Mar Riveiro-Barciela Maria Buti Ariadna Rando-Segura
Roser Ferrer Tomás Pumarola Cristina Andrés Andrés Antón
Francisco Rodríguez-Frías Josep Quer†

2026-04-06

**Abstract**

Through studies of HEV quasispecies with exceptional diversity yet high functionality, we developed novel indicators—such as quasispecies maturity and synonymy—that reveal the complex interplay between genetic diversity and functional stability. These concepts provide insights into flat-like quasispecies, characterized by dynamic, low-dominance haplotype distributions. We propose an expanded analytical framework using these indicators, which offer more intuitive, structure-focused interpretations than classical diversity indices. This approach aligns closely with quasispecies fitness and may have substantial implications for clinical applications, enhancing our understanding of viral population dynamics, disease progression, and therapeutic interventions. Two limiting quasispecies models are proposed bridging quasispecies origins and endpoints.

## In-silico study with simulated quasispecies structures

The hypothesis underlying this simulation is that a quasispecies, free from external evolutionary pressures, will evolve through newly generated variants arising from replication errors. Less fit or deleterious variants will be purged from the quasispecies, while those with synonymous substitutions, neutral, or quasi-neutral mutations will increase in frequency based on their fitness potential in competition with existing genomes. Over an unlimited period of evolution, the founding genome will gradually lose dominance, while the promoted variants will become progressively more abundant. The endpoint represents a quasispecies characterized by a high number of equally fit genomes at shared low frequencies, maintained in dynamic equilibrium, with multiple low-cost escape routes against external interferences such as the immune system or antiviral treatments, owing to the broad genetic space occupied by the multiple variants.

Rank-abundance haplotype distributions (RAD) (Whittaker 1965; Saeedghalati et al. 2017) based on power law functions are employed as mock quasispecies to illustrate the concepts discussed in

*Corresponding authors josep.gregori@gmail.com
†and josep.quer@vhir.org

the main text. These distributions may be also represented by the rank-abundance cumulative frequency distribution (RACD). The goal is to cover the full range of possible RAD, RACD, and relative logarithmic evenness (RLE) (Jost 2010; Studeny et al. 2011) profiles.

Quasispecies structures, each consisting of a fixed number of one thousand haplotypes with frequency distributions ranging from highly peaked and skewed to flat and homogeneous, are simulated. Each is illustrated by its corresponding RAHD, RACD, and RLE profiles and characterized by quasispecies structure indicators. These simulations serve as toy models relative to real-world viral populations, with the fixed number of haplotypes across simulations representing a key limitation. Nonetheless, they offer a useful framework for understanding the fundamental principles underlying the quasispecies structure indicators proposed in the main text, covering a wide spectrum of complexity. The simulated sequence of quasispecies may be interpreted as the temporal evolution of a single quasispecies over an unlimited period, or as independent viral populations of one thousand haplotypes exhibiting increasing maturity. This simulation mimics the observed diversity spectra and behavior in the clinical samples described in the main text.

Quasispecies populations are simulated using power-law rank–abundance distributions of haplotype frequencies, following Zipf-Pareto models (Newman 2005; Callaghan et al. 2023; Da Silva and Matsushita 2023). This model is convenient for multiple reasons: A single parameter, $\alpha$, controls the distribution profile, capturing viral diversity spectra without introducing piecewise complexity, and is capable of fitting a wide range of tail distributions, including those with heavy tails containing many rare haplotypes.

A quasispecies following a Zipf-Pareto distribution with shape parameter $\alpha$ has rank-abundances $A(r) \propto r^{-\alpha}$, where $r = 1 \ldots H$ denotes the haplotype rank. The corresponding normalized frequencies are given by:

$$p_r = K\, r^{-\alpha}, \quad \text{where} \quad K = \left( \sum_{r=1}^{H} r^{-\alpha} \right)^{-1}$$

ensuring $\sum_{r=1}^{H} p_r = 1$. This formulation spans distributions that range from peaked dominance ($\alpha = 5$) to high evenness nearly uniform distributions ($\alpha = 0.11$), making it suitable for modeling quasispecies diversity.

## Thousand-haplotype quasispecies

Fifteen quasispecies, each composed of one thousand haplotypes whose frequencies are generated as described above, are simulated to cover the entire range from peaked-fitness quasispecies, characterized by a highly dominant haplotype, to flat-fitness quasispecies, where all haplotypes occur at comparable frequencies. The selected $\alpha$ values are as follows: 5.00, 2.50, 2.00, 1.67, 1.43, 1.25, 1.11, 1.00, 0.87, 0.76, 0.65, 0.54, 0.43, 0.32, and 0.11.

Additionally, two bracketing quasispecies are defined. Quasispecies A, consists of a master haplotype at a frequency approaching 1, and 999 variant haplotypes each with a frequency of $(1. \times 10^{-50}/\ 999)$. Quasispecies Z, comprises one thousand haplotypes, each occurring at a frequency of $1. \times 10^{-3}$.

## Quasispecies distribution profiles

Beyond the haplotype distributions representation as rank-abundance cumulative curves (Whittaker 1965; Saeedghalati et al. 2017) (Figure B1), these quasispecies may be characterized by their relative

logarithmic evenness (RLE) (Jost 2010; Studeny et al. 2011) profiles (Figure B2).

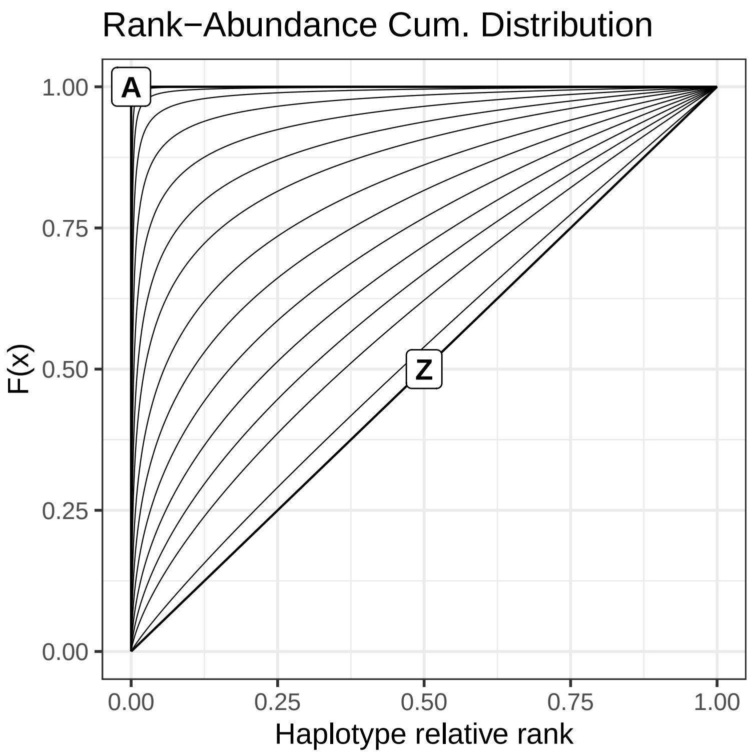


Figure B1: Rank-abundance cumulative distribution for each quasispecies. Haplotypes ranked in decreasing abundance order.

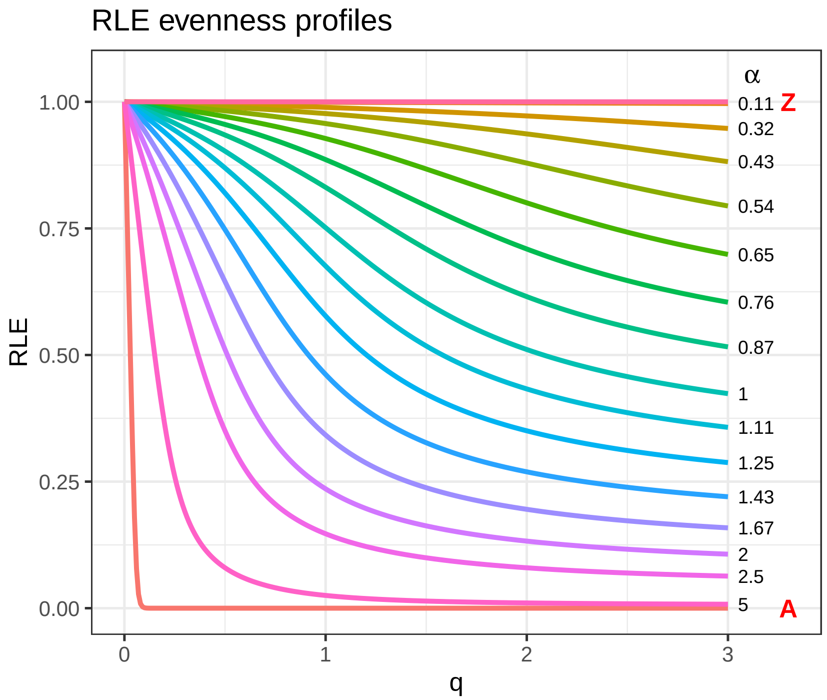


Figure B2: Evenness profiles of simulated quasispecies structures composed of one thousand haplotypes. The corresponding alpha values of the simulated rank-abundance distributions are indicated next to each curve on the right. Quasispecies A and Z are labeled accordingly.

## Quasispecies structure indicators

The quasispecies structure indicators used (Gregori, Colomer-Castell, et al. 2024; Gregori, Ibañez-Lligoña, et al. 2024; Gregori et al. 2025) are listed in Table B1, along with their expected values in the two extreme cases: regular (peaked) quasispecies, as those developing under short-duration acute infections, and flat-like quasispeces, as those developing after prolonged periods under chronic infection with high viral loads (Gregori, Colomer-Castell, et al. 2024).

This is an expanded set of quasispecies structure indicators relative to those used in the main text, including the full set developed in recent studies by our group (Gregori, Colomer-Castell, et al. 2024; Gregori, Ibañez-Lligoña, et al. 2024; Gregori et al. 2025) for comparative purposes. There is high flexibility in selecting the set of indicators, provided they have well-defined values in the two limiting states. Their correlations are accounted for in the principal components decomposition by retaining the first two components, PC1 and PC2..

Table B1: Quasispecies structure indicators and expected level per quasispecies type.

| Feature | Description | Regular Qs | Flat Qs |
|---|---|---|---|
| Top10 | Fraction of reads for top 10 hpl. | High | Low |
| Master | Dominant haplotype frequency | High | Low |
| Rare1 | Fraction of reads for hpl $\leq 1\%$ | Low | High |
| $RLE_1$ | Relative logarithmic evenness at $q = 1$ | Low | High |
| $RLE_2$ | Relative logarithmic evenness at $q = 2$ | Low | High |
| $RLE_3$ | Relative logarithmic evenness at $q = 3$ | Low | High |
| $RLE_\infty$ | Relative logarithmic evenness at $q = \infty$ | Low | High |
| $I_3$ | Normalized RLE integral for $q = 0 : 3$ | Low | High |
| $R_k$ | Evenness of top k haplotypes (k= 5, 10) | Low | High |

All these indicators may be calculated directly from the vector of haplotype ranked abundances $\hat{\mathbf{P}} = (\hat{p_1}, \hat{p_2}, \cdots, \hat{p_H})$, with $H$ the number of haplotypes, and $\hat{p_1} \geq \hat{p_2} \geq \cdots \geq \hat{p_H}$.

- Quasispecies fitness fractions:

$$Top10 = \sum_{k=1}^{10} \hat{p}_i$$

$$Master = max(\hat{p}_i) = \hat{p}_1$$

$$Rare1 = \sum_{i}^{H} \hat{p}_i \; I(\hat{p}_i \leq 0.01)$$

where $I(.)$ is the indicator function, yielding 0 or 1 depending on whether the expression inside the parentheses is false or true.

- Relative logarithmic evenness indicators:

$$RLE_q = \frac{log(D(q, \hat{\mathbf{P}}))}{log(H)}$$

where $D(q, \hat{\mathbf{P}})$ is the Hill number of order $q$, with $q = 1, 2, 3, \infty$.

$$D(q, \hat{\mathbf{P}}) = \left( \sum_{i=1}^{H} \hat{p}_i^q \right)^{1/(1-q)}$$

- Top haplotypes evenness indicators:

$$R_k = \frac{k \cdot \hat{p}_k}{\sum_i^{k-1} \hat{p}_i}$$

the ratio of the k-th ranked haplotype frequency to the mean of the $(k-1)$ top ranked haplotype frequencies

## Limiting quasispecies states

Two idealized models of extreme quasispecies states were considered in this study as the limiting forms of quasispecies structure. Quasispecies AM represents a highly peaked population, characterized by a dominant haplotype with a frequency approaching 100% and a single variant genome present at an infinitesimal frequency. In contrast, quasispecies ZM represents a completely flat population, consisting of an infinite number of haplotypes, each occurring at infinitesimally small and equal frequencies.

Model AM can be viewed as representing the emergence or early stage of a quasispecies and is expected to resemble those found in acute, short-duration infections. Conversely, Model ZM corresponds to the theoretical endpoint of quasispecies evolution after an infinitely long infection period and may approximate the structure of quasispecies in chronic, long-duration infections (Gregori, Ibañez-Lligoña, et al. 2024). The quasispecies structure indicator values corresponding to these two reference models are presented in Table B2.

Table B2: Quasispecies structure indicator values characterizing the two idealized limiting states, AM and ZM.

| Model | Top10 | Master | Rare1 | RLE1 | RLE2 | RLE3 | RLEinf | R5 | R10 |
|---|---|---|---|---|---|---|---|---|---|
| AM | 1.000 | 1.000 | 0.000 | 0.000 | 0.000 | 0.000 | 0.000 | 0.000 | 0.000 |
| ZM | 0.000 | 0.000 | 1.000 | 1.000 | 1.000 | 1.000 | 1.000 | 1.000 | 1.000 |

The zeroes for *Top10* and *Master*, in Model ZM, should be interpreted as an infinitesimal frequency $\epsilon$, that is $\epsilon > 0$ and $\epsilon < 1/n$ for any positive integer $n$, rather than strictly zero. In biochemical terms this $\epsilon$ may be interpreted as one to a few or several molecules, in the context of a high viral titer. Similarly, the zeroes for *Rare1*, in Model AM, should be interpreted as a result of several variants other than the master haplotype, at infinitesimal frequencies, $\epsilon$. However, we represent $\epsilon$ formally as 0.

## Quasispecies structure characterization

Table B3 lists the quasispecies structure indicator values that characterize each simulated quasispecies structure in terms of fitness fractions and evenness. The quasispecies are listed in order of increasing *I3* values, representing progressive maturity.

Table B3: Quasispecies structure indicators

| ID | Top10 | Master | Rare1 | RLE1 | RLE2 | RLE3 | RLEinf | I3 | R5 | R10 |
|---|---|---|---|---|---|---|---|---|---|---|
| AM | 1.0000 | 1.0000 | 0.0000 | 0.0000 | 0.0000 | 0.0000 | 0.0000 | 0.0000 | 0.0000 | 0.0000 |
| A | 1.0000 | 1.0000 | 0.0000 | 0.0000 | 0.0000 | 0.0000 | 0.0000 | 0.0137 | 0.0000 | 0.0000 |
| Qs_5 | 1.0000 | 0.9644 | 0.0055 | 0.0252 | 0.0104 | 0.0079 | 0.0052 | 0.0803 | 0.0015 | 0.0001 |
| Qs_2.5 | 0.9854 | 0.7455 | 0.0383 | 0.1470 | 0.0798 | 0.0634 | 0.0425 | 0.2089 | 0.0693 | 0.0239 |
| Qs_2 | 0.9427 | 0.6083 | 0.0804 | 0.2357 | 0.1325 | 0.1067 | 0.0720 | 0.2835 | 0.1366 | 0.0645 |
| Qs_1.67 | 0.8601 | 0.4758 | 0.1399 | 0.3421 | 0.1953 | 0.1587 | 0.1075 | 0.3618 | 0.2073 | 0.1183 |
| Qs_1.43 | 0.7418 | 0.3554 | 0.2365 | 0.4616 | 0.2693 | 0.2201 | 0.1498 | 0.4433 | 0.2755 | 0.1780 |
| Qs_1.25 | 0.6111 | 0.2575 | 0.3541 | 0.5771 | 0.3503 | 0.2876 | 0.1964 | 0.5227 | 0.3370 | 0.2369 |
| Qs_1.11 | 0.4899 | 0.1844 | 0.4748 | 0.6757 | 0.4330 | 0.3572 | 0.2448 | 0.5958 | 0.3913 | 0.2921 |
| Qs_1 | 0.3913 | 0.1336 | 0.5752 | 0.7515 | 0.5109 | 0.4238 | 0.2914 | 0.6589 | 0.4380 | 0.3414 |
| Qs_0.87 | 0.2822 | 0.0850 | 0.7072 | 0.8312 | 0.6153 | 0.5161 | 0.3568 | 0.7370 | 0.4976 | 0.4065 |
| Qs_0.76 | 0.2037 | 0.0548 | 0.8058 | 0.8859 | 0.7093 | 0.6042 | 0.4205 | 0.8024 | 0.5517 | 0.4672 |
| Qs_0.65 | 0.1412 | 0.0336 | 0.8926 | 0.9275 | 0.8005 | 0.6987 | 0.4912 | 0.8632 | 0.6090 | 0.5329 |
| Qs_0.54 | 0.0946 | 0.0198 | 0.9557 | 0.9571 | 0.8790 | 0.7943 | 0.5678 | 0.9156 | 0.6693 | 0.6032 |
| Qs_0.43 | 0.0618 | 0.0113 | 0.9887 | 0.9768 | 0.9366 | 0.8818 | 0.6494 | 0.9558 | 0.7324 | 0.6777 |
| Qs_0.32 | 0.0395 | 0.0062 | 1.0000 | 0.9890 | 0.9721 | 0.9474 | 0.7350 | 0.9822 | 0.7980 | 0.7559 |
| Qs_0.11 | 0.0162 | 0.0019 | 1.0000 | 0.9990 | 0.9979 | 0.9966 | 0.9068 | 1.0000 | 0.9290 | 0.9138 |
| Z | 0.0100 | 0.0010 | 1.0000 | 1.0000 | 1.0000 | 1.0000 | 1.0000 | 1.0000 | 1.0000 | 1.0000 |
| ZM | 0.0000 | 0.0000 | 1.0000 | 1.0000 | 1.0000 | 1.0000 | 1.0000 | 1.0000 | 1.0000 | 1.0000 |

### Principal components analysis

Despite measuring different aspect of quasispecies structure and diversity these indicator are highly correlated. This correlation, however, allows for a highly approximated representation of the quasispecies in the space of diversity and structure by projecting the quasispecies on the plane PC1/PC2 defined by the two principal components (PC) in a Principal Components Analysis (PCA) of the matrix of quasispecies structure indicator values. PCA involves finding the eigenvectors and eigenvalues of the indicators' covariance matrix (Jolliffe 2002; Jolliffe and Cadima 2016; Demidovich and Maron 1981) (Table B4 and Figure B3).

Table B4: Importance of principal components

| | PC1 | PC2 | PC3 | PC4 |
|---|---|---|---|---|
| Standard deviation | 3.0962 | 0.5766 | 0.2614 | 0.1066 |
| Proportion of Variance | 0.9586 | 0.0332 | 0.0068 | 0.0011 |
| Cumulative Proportion | 0.9586 | 0.9919 | 0.9987 | 0.9999 |

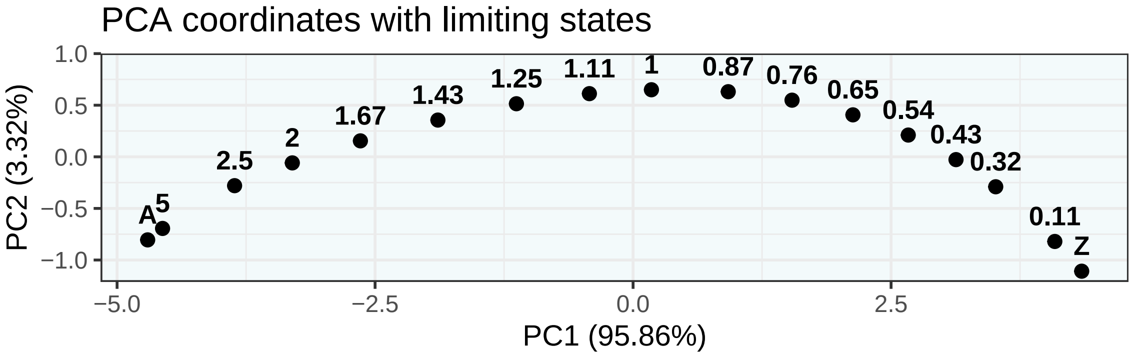


Figure B3: Simulated quasispecies structures projected onto PC1/PC2 plane. Quasispecies labeled with alpha values.

### The AM-to-ZM path

A parabola may be fitted by linear regression to the projection of the quasispecies on the PC1/PC2 plane, with adjusted $R^2$ 0.962. Table B5 lists corresponding coefficients, standard errors and p-vlaues. Figure B4 shows the parabola over the quasispecies plotted on the PC1/PC2 plane. A cubic polynomial may be fitted with an adjusted $R^2$ of 0.9893; however the parabolic representation is retained due to its theoretical implications.

Table B5: Fitted parabola by linear regression.

| | Estimate | Std. Error | t value | Pr(>\|t\|) |
|---|---|---|---|---|
| (Intercept) | 0.6899 | 0.0440 | 15.6787 | 0.0000 |
| PC1 | -0.0307 | 0.0093 | -3.3202 | 0.0051 |
| I(PC1^2) | -0.0765 | 0.0038 | -20.0498 | 0.0000 |

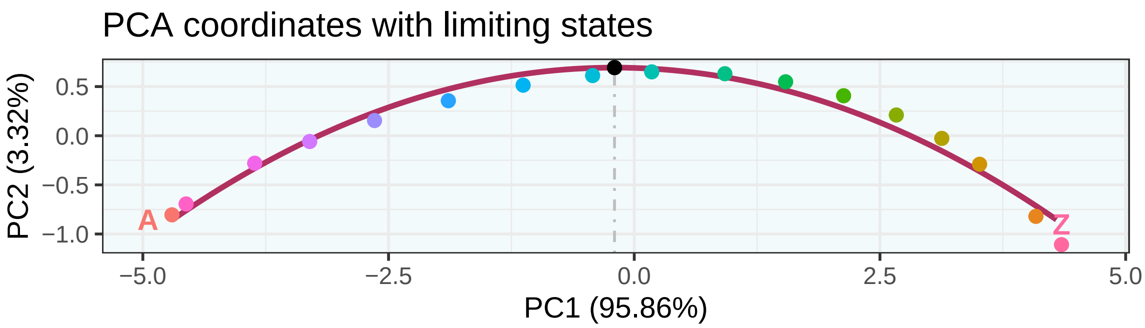


Figure B4: Scatterplot of the simulated quasispecies structures on the PC1/PC2 plane, with the two bracketing quasispecies A and Z. Red curve: cubic parabola fitted by linear regression. Black dot: parabola apex.

Table B6: Quasispecies nearest to the parabola apex.

| ID | Top10 | Master | Rare1 | RLE1 | RLE2 | RLE3 | RLEinf | I3 | R5 | R10 |
|---|---|---|---|---|---|---|---|---|---|---|
| Qs_1.11 | 0.4899 | 0.1844 | 0.4748 | 0.6757 | 0.433 | 0.3572 | 0.2448 | 0.5958 | 0.3913 | 0.2921 |

### PC-based distance form Model A

The distance of each quasispecies from Model A (AM), *PC.d2A.ln*, on the *PC1/PC2* plane, provides smoothed quasispecies maturity scores on the *Peaked-to-Flat* fitness landscape axis. Alternatively, instead of the linear distance from AM, the arc length from AM to each quasispecies on the fitted parabola may be computed. Given the parabola $f(x) = a + b \cdot x + c \cdot x^2$, the arc length between $x = x_1$ and $x = x_2$, is calculated as the definite integral:

$$L(x_1, x_2) = \int_{x_1}^{x_2} \sqrt{1 + f'(x)^2}\; dx$$

The analytic solution to the above definite integral, providing the exact parabola arc length between two $x$ points, is given by

$$L(x_1, x_2) = \int_{x_1}^{x_2} \sqrt{1 + f'(x)^2}\; dx = \int_{x_1}^{x_2} \sqrt{1 + (2cx + b)^2}\; dx =$$

$$= \frac{1}{4c}\left[(2cx + b)\sqrt{1 + (2cx + b)^2} + \ln\left(\left|(2cx + b) + \sqrt{1 + (2cx + b)^2}\right|\right)\right]_{x_1}^{x_2}$$

A normalized score is obtained dividing these distances by the corresponding AM-to-ZM distance.

### Full dimension distance from Model A

In having a single quasispecies, or very few, a PCA is not feasible. In this case, we may obtain maturity scores from the full dimension distance of each quasispecies from AM, instead of the 2D-PCA-based arc length from AM. In this case the score will be slightly larger.

### Quasispecies maturity scores

In addition to the distance from AM, the area under the RLE profile between $q = 0$ and $q = 3$, denoted $I_3$, provides an integrated score of quasispecies evenness that can also serve as a maturity score (Figure B5). The most conservative maturity score is obtained from the PCA-parabola arc length.

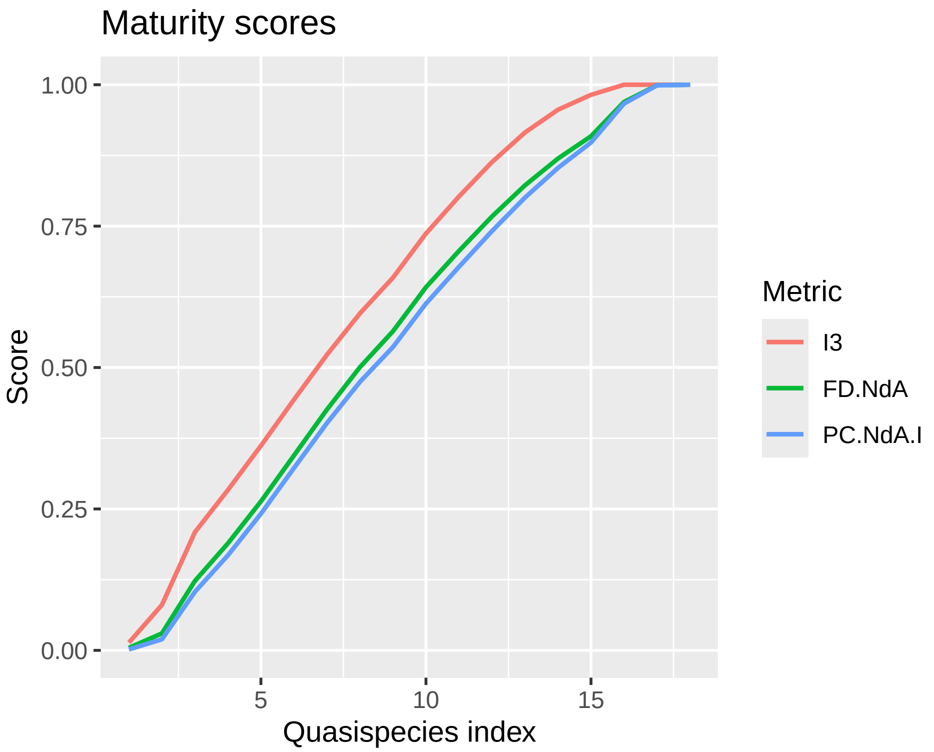


Figure B5: Maturity scores of the simulated quasispecies structures. FD.NdA: Full-dimension distance from AM; PC.NdA.I: 2D-PCA parabola arc length from AM (analytical value). Quasispecies indices on x-axis as per Table B3.

### Evolution of quasispecies indicators

Table B7 and Figure B6 illustrate the evolution of quasispecies structure indicators as the population matures, as measured by the $NdA$ score. The plot highlights two critical points: the intersection of the $Master$ and $RLE_\infty$ curves, and the apex of the parabola. The intersection between $Master$ and $RLE_\infty$ corresponds to the condition $-log(M) = M\ log(H)$, which can be rewritten as $M = 1/H^M$, (for example, with $H = 1000$, $M = 21.95\%$). Here $H$ denotes the number of haplotypes, and $M$ represents the frequency of the master haplotype. By interpolating the curves, we obtain a numerical estimate of $M = 22.1\%$, very close to the theoretical value (Figure B6). Interestingly, in our case, the curves corresponding to $Top10$ and $I_3$ intersect at the same $NdA$ value as $Master$ and $RLE_\infty$ curves. $Top10$ is a dominance-related metric, whereas $I_3$ reflects evenness within the population.

The $NdA$ value at which these intersections occur may thus be interpreted as a boundary between the two evolutionary phases. From State A, the evolution is dominated by diversification, whereas as the system approaches State Z, increasing evenness overtakes increasing diversity. Quasispecies with $RLE_\infty > M$ are expected to exhibit structures closer to ZM than to AM, possibly including related functional aspects such as high viremia, improved fitness, and reduced response to antiviral treatments.

Table B7: Quasispecies structure indicators and maturity score.

| ID | NdA | Top10 | Master | Rare1 | RLE1 | RLE2 | RLE3 | RLEinf | I3 | R5 | R10 |
|---|---|---|---|---|---|---|---|---|---|---|---|
| A | 0.0016 | 1.0000 | 1.0000 | 0.0000 | 0.0000 | 0.0000 | 0.0000 | 0.0000 | 0.0137 | 0.0000 | 0.0000 |
| Qs_5 | 0.0195 | 1.0000 | 0.9644 | 0.0055 | 0.0252 | 0.0104 | 0.0079 | 0.0052 | 0.0803 | 0.0015 | 0.0001 |
| Qs_2.5 | 0.1035 | 0.9854 | 0.7455 | 0.0383 | 0.1470 | 0.0798 | 0.0634 | 0.0425 | 0.2089 | 0.0693 | 0.0239 |
| Qs_2 | 0.1680 | 0.9427 | 0.6083 | 0.0804 | 0.2357 | 0.1325 | 0.1067 | 0.0720 | 0.2835 | 0.1366 | 0.0645 |
| Qs_1.67 | 0.2415 | 0.8601 | 0.4758 | 0.1399 | 0.3421 | 0.1953 | 0.1587 | 0.1075 | 0.3618 | 0.2073 | 0.1183 |
| Qs_1.43 | 0.3223 | 0.7418 | 0.3554 | 0.2365 | 0.4616 | 0.2693 | 0.2201 | 0.1498 | 0.4433 | 0.2755 | 0.1780 |
| Qs_1.25 | 0.4019 | 0.6111 | 0.2575 | 0.3541 | 0.5771 | 0.3503 | 0.2876 | 0.1964 | 0.5227 | 0.3370 | 0.2369 |
| Qs_1.11 | 0.4746 | 0.4899 | 0.1844 | 0.4748 | 0.6757 | 0.4330 | 0.3572 | 0.2448 | 0.5958 | 0.3913 | 0.2921 |
| Qs_1 | 0.5363 | 0.3913 | 0.1336 | 0.5752 | 0.7515 | 0.5109 | 0.4238 | 0.2914 | 0.6589 | 0.4380 | 0.3414 |
| Qs_0.87 | 0.6131 | 0.2822 | 0.0850 | 0.7072 | 0.8312 | 0.6153 | 0.5161 | 0.3568 | 0.7370 | 0.4976 | 0.4065 |
| Qs_0.76 | 0.6780 | 0.2037 | 0.0548 | 0.8058 | 0.8859 | 0.7093 | 0.6042 | 0.4205 | 0.8024 | 0.5517 | 0.4672 |
| Qs_0.65 | 0.7414 | 0.1412 | 0.0336 | 0.8926 | 0.9275 | 0.8005 | 0.6987 | 0.4912 | 0.8632 | 0.6090 | 0.5329 |
| Qs_0.54 | 0.8005 | 0.0946 | 0.0198 | 0.9557 | 0.9571 | 0.8790 | 0.7943 | 0.5678 | 0.9156 | 0.6693 | 0.6032 |
| Qs_0.43 | 0.8530 | 0.0618 | 0.0113 | 0.9887 | 0.9768 | 0.9366 | 0.8818 | 0.6494 | 0.9558 | 0.7324 | 0.6777 |
| Qs_0.32 | 0.8978 | 0.0395 | 0.0062 | 1.0000 | 0.9890 | 0.9721 | 0.9474 | 0.7350 | 0.9822 | 0.7980 | 0.7559 |
| Qs_0.11 | 0.9667 | 0.0162 | 0.0019 | 1.0000 | 0.9990 | 0.9979 | 0.9966 | 0.9068 | 1.0000 | 0.9290 | 0.9138 |
| Z | 0.9989 | 0.0100 | 0.0010 | 1.0000 | 1.0000 | 1.0000 | 1.0000 | 1.0000 | 1.0000 | 1.0000 | 1.0000 |

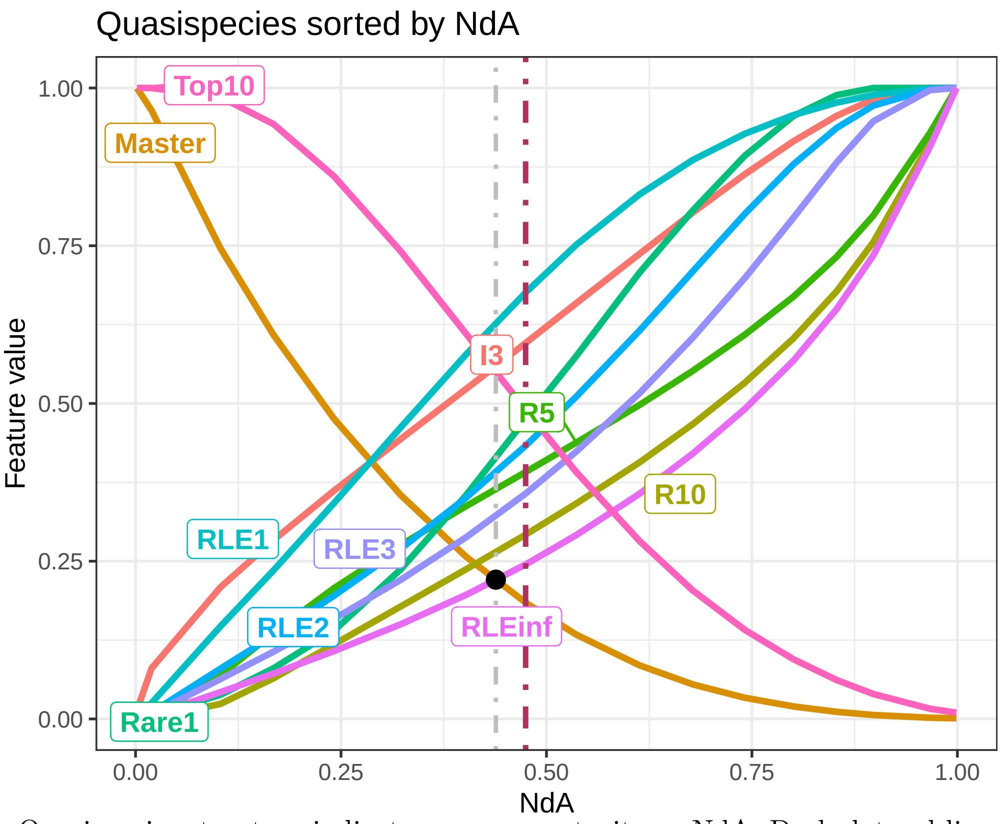


Figure B6: Quasispecies structure indicators versus maturity as NdA. Dash-dot red line: parabola apex. Black dot and dash-dot gray line: Crossing of Master with RLEinf at N.d2A = 0.438, with Master = 0.221

## References


Callaghan, C. T., et al. (2023) ‘Unveiling Global Species Abundance Distributions’, *Nat Ecol Evol*, 7: 1600–9, https://doi.org/10.1038/s41559-023-02173-y.

Da Silva, S., and R. Matsushita (2023) ‘Power Laws Govern the Abundance Distribution of Birds by Rank’, *Birds*, 4/2: 171–8, https://doi.org/10.3390/birds4020014.

Demidovich, B. P., and I. A. Maron (1981) *Computational Mathematics* (Moscow: Mir Publishers).

Gregori, J., M. Ibañez-Lligoña et al. (2024) ‘Association of Liver Damage and Quasispecies Maturity in Chronic HCV Patients: The Fate of a Quasispecies’, *Microorganisms*, 12/11: 2213:1–15, https://doi.org/10.3390/microorganisms12112213.

Gregori, J., S. Colomer-Castell et al. (2024) ‘In-Host Flat-Like Quasispecies: Characterization Methods and Clinical Implications’, *Microorganisms*, 12/5: 1011:1–14, https://doi.org/10.3390/microorganisms12051011.

Gregori, J., et al. (2025) ‘Viral quasispecies inference from single observations—Mutagens as accelerators of quasispecies maturity’, *Microorganisms*, 13/9: 2029, https://doi.org/10.3390/microorganisms13092029.

Jolliffe, I. T. (2002) *Principal Component Analysis* (2nd ed., New York, NY: Springer-Verlag).

Jolliffe, I. T., and J. Cadima (2016) ‘Principal Component Analysis: A Review and Recent Developments’, *Phil. Trans. R. Soc. A*, 374: 20150202, https://doi.org/10.1098/rsta.2015.0202.

Jost, L. (2010) ‘The Relation Between Evenness and Diversity.’, *Diversity*, 2/2, https://doi.org/10.3390/d2020207.

Newman, M. E. J. (2005) ‘Power Laws, Pareto Distributions and Zipf’s Law’, *Contemporary Physics*, 46/5: 323–51, https://doi.org/10.1080/00107510500052444.

Saeedghalati, M., et al. (2017) ‘Quantitative Comparison of Abundance Structures of Generalized Communities: From b-Cell Receptor Repertoires to Microbiomes’, *PLoS Comput Biol*, 13/1: e1005362, https://doi.org/10.1371/journal.pcbi.1005362.

Studeny, A. C., et al. (2011) ‘Goodness-of-Fit Measures of Evenness: A New Tool for Exploring Changes in Community Structure.’, *Ecosphere*, 2/2, https://doi.org/10.1890/ES10-00074.1.

Whittaker, R. H. (1965) ‘Dominance and Diversity in Land Plant Communities: Numerical Relations of Species Express the Importance of Competition in Community Function and Evolution’, *Science*, 147/3655: 250–60, https://doi.org/10.1126/science.147.3655.250.

# Appendix A

## R code

```
### Load required libraries
library(tidyverse)
library(stringr)
library(gridExtra)
library(ggrepel)

###    w  vector of observed frequencies
###    q  exponent
qD <- function(p,q)
{ if(q==0) return(length(p))
  if(q==1) return( exp(-sum(p*log(p))) )
  if(q==Inf) return(1/max(p))
  if(q==-Inf) return(1/min(p))
  sum(p^q)^(1/(1-q))
}

###    w  vector of observed counts or frequencies
qD.profile <- function(w,q=NULL)
{ if(is.null(q))
    q <- c(seq(0,3,0.001))
  p <- w/sum(w)
  dv <- numeric(length(q))
  sapply(1:length(q),function(i) qD(p,q[i]))
}

### Power-law parameters
n <- 1000
alpha <- c(5.00,2.50,2.00,1.67,1.43,1.25,1.11,1.00,0.87,0.76,0.65,0.54,
           0.43,0.32,0.11)
np <- length(alpha)

RAD_Pareto <- function(n,shape)
{
  # Simulate species abundances following a Pareto distribution
  abundance <- (1:n)^(-shape)
  freq <- abundance/sum(abundance)
  sorted_freq <- sort(freq, decreasing = TRUE)
  return(sorted_freq)
}

### Generate quasispecies
p.m <- matrix(0,nrow=1000,ncol=length(alpha))
for(j in 1:length(alpha))
   p.m[,j] <- RAD_Pareto(n,alpha[j])
```

```
colnames(p.m) <- paste('Qs',round(alpha,2),sep='_')

### Add limiting quasispecies A and Z
p.m <- cbind(A=c(1.-1.e-50,rep(1.e-50/999,999)),
             p.m,
             Z=rep(0.001,1000))


### Solve zeros
p.m[p.m < .Machine$double.xmin] <- .Machine$double.xmin

### Normalitzation
p.m <- apply(p.m,2,function(x) x/sum(x))

### Cumulative frequency distribution
cum.p.m <- apply(p.m,2,function(x) cumsum(x))

### Rank-abundance cumulative distribution
RACD.df <- data.frame(x=(1:1000)/1000,cum.p.m)

### Normalització a master = 1
N.p.m <- apply(p.m,2,function(x) x/max(x))

### RACD elbow
curve_elbow <- function(A,B,x,y)
{
  Dk <- abs((B[2]-A[2])*x - (B[1]-A[1])*y + B[1]*A[2] - B[2]*A[1])
  k <- which.max(Dk)[1]
  return(c(x[k],y[k]))
}

A <- c(1,0)
B <- c(1000,1)
e.dt <- t(apply(cum.p.m,2,function(x) curve_elbow(A,B,1:nrow(cum.p.m),x)))
colnames(e.dt) <- c('Rank','Ye')
e.df <- data.frame(e.dt) %>%
  mutate(RelRank=Rank/1000)

e.df['Z','Rank'] <- 362
e.df['Z','Ye'] <- 0.362
e.df['Z','RelRank'] <- 0.362


### Rank-abundance cumulative distribution for each quasispecies.
### Haplotypes ranked in decreasing abundance order.
P <- RACD.df %>%  pivot_longer(-x,names_to='ID',values_to='F') %>%
  ggplot() +
```

```
  geom_line(aes(x=x,y=F,group=ID),lwd=0.2) +
  # geom_line(aes(x=RelRank,y=Fr,group=1),col='blue',lwd=0.8,data=e.df) +
  annotate('segment',x=0,xend=1,y=0,yend=1,lwd=1) +
  annotate('segment',x=0,xend=0,y=0,yend=1,lwd=1) +
  annotate('segment',x=0,xend=1,y=1,yend=1,lwd=1) +
  annotate('label',x=0,y=1,label='A',fontface='bold') +
  annotate('label',x=0.5,y=0.5,label='Z',fontface='bold') +
  ylim(0,1) +
  labs(x='Haplotype relative rank',y='F(x)',
       title='Rank-Abundance Cum. Distribution') +
  theme_bw()
P

### Compute quasispecies hill numbers profile
q=seq(0,3,0.01)
qD.prfs <- apply(p.m,2,function(p) qD.profile(p,q))
qD.prfs <- data.frame(q=q,qD.prfs)

###  Compute quasispecies RLE profiles
RLE.prfs <- qD.prfs %>%
  mutate(across(-q,~log(.)/log(1000)))

### Trapezoidal rule in numeric integration
h <- RLE.prfs$q[2]-RLE.prfs$q[1]
I.trp <- 1/3 * h/2 * (2*apply(RLE.prfs[-c(nrow(RLE.prfs)),-1],2,sum) +
                RLE.prfs[1,-1] + RLE.prfs[nrow(RLE.prfs),-1])
It <- data.frame(ID=names(I.trp),
                 I=unlist(I.trp),x=3.05,y=unlist(RLE.prfs[nrow(RLE.prfs),-1]),
                 Lbl=paste(round(I.trp,3)))

Qs.RLE.lb <- data.frame(ID=c('A','Z'),x=3.3,y=c(0,1))
lb.q <- data.frame(ID=colnames(RLE.prfs),y=unlist(RLE.prfs[301,]))[3:15,] %>%
  mutate(x=3.05,alpha=as.numeric(substring(ID,4)))

### Evenness profiles of simulated quasispecies structures of one thousand
##  haplotypes.  Alpha values next to each curve on the right.
P <- RLE.prfs %>%
  pivot_longer(-q,names_to='Qs',values_to='RLE') %>%
  ggplot() +
  geom_line(aes(x=q,y=RLE,group=Qs,col=Qs),lwd=1) +
  geom_text(aes(x=x,y=y,label=ID),col='red',fontface='bold',data=Qs.RLE.lb) +
  geom_text(aes(x=x,y=y,label=alpha),adj=0,size=8/.pt,data=lb.q) +
  annotate('text',x=3.12,y=1.05,label=expression(alpha),fontface='bold') +
  xlim(0,3.3) +
  labs(title='RLE evenness profiles') +
  theme_bw() +
  theme(legend.position='none')
```

```
P

### Diversities and structure indicators
div.nms <- c('Top10','Master','Rare1',
             'q0','q1','q2','q3','qinf',
             'R5','R10')

###  Function computing selected diversity indices
diversity <- function(nr)
{
   p <- nr/sum(nr)
   q_0 <- length(p)               # Hill number for q=0
   q_1 <- exp(-sum(p*log(p)))     # Hill number for q=1
   q_2 <- 1/sum(p^2)              # Hill number for q=2
   q_3 <- 1/sqrt(sum(p^3))        # Hill number for q=3
   q_inf <- 1/max(p)              # Hill number for q=Infinity

   p <- sort(p,decreasing=TRUE)  # Frequencies in decreasing order
   Master <- p[1]                 # Master frequency
   Top10 <- sum(p[1:10])            # Top 5 haplotypes, reads fraction
   Rare1 <- sum(p[p<=0.01])       # Fraction of reads for all Hpl <1%
   R5 <- p[5]/mean(p[1:5])        # Evenness in top 5 haplotypes
   R10 <- p[10]/mean(p[1:10])     # Evenness in top 10 haplotypes

   vdiv <- c(Top10,Master,Rare1,q_0,q_1,q_2,q_3,q_inf,R5,R10)
   names(vdiv) <-
       c('Top10','Master','Rare1','q0','q1','q2','q3','qinf',
             'R5','R10')
   return(vdiv)
}

### Compute quasispecies diversity and structure indicators
divs <- t(apply(p.m,2,function(p) diversity(p)))
divs.df <- data.frame(ID=rownames(divs),divs)
row.names(divs.df) <- NULL
mat.vals <- divs.df %>%
  mutate(RLE1=log(q1)/log(q0),
         RLE2=log(q2)/log(q0),
         RLE3=log(q3)/log(q0),
         RLEinf=log(qinf)/log(q0)) %>%
  left_join(It %>% select(ID,I),by='ID') %>%
  dplyr::rename(I3=I) %>%
  mutate(I3=ifelse(I3>1,1,I3)) %>%
  select(-starts_with('q')) %>%
  relocate(Top10,.before='Master') %>%
  relocate(R5,R10,.after='I3')
```

```r
### Add limiting models
AM <- c(1,1,0,0,0,0,0,0,0,0)
ZM <- c(0,0,1,1,1,1,1,1,1,1)
AM.ZM <- rbind(AM=AM,ZM=ZM)
colnames(AM.ZM) <- colnames(mat.vals)[-1]
AM.ZM <- data.frame(AM.ZM) %>%
  rownames_to_column(var='ID')

pprt <- rbind(AM.ZM[1,],mat.vals,AM.ZM[2,])
rownames(pprt) <- NULL
pprt %>%
  knitr::kable(caption="Quasispecies structure indicators",
               digits=4)


### Principal Components Annalysis
pca <- prcomp(data.matrix(mat.vals[,-1]),scale.=TRUE)
summary(pca)$importance[,1:4] %>%
  knitr::kable(digits=4,caption='Importance of principal components')
### PC axis labels
pca.pct <- round(summary(pca)$importance[2,1:2] * 100,2)
pca.lbls <- paste(c('PC1','PC2'),' (',pca.pct,'%)',sep='')

###  Take care of signs indeterminacy under PCA
x.sgn <- 1
if( pca$x[1,1] > 0 ) x.sgn <- -1  # A left, Z right.
y.sgn <- 1
if( pca$x[1,2] > 0 ) y.sgn <- -1  # Appex above.
pca$x[,1] <- pca$x[,1] * x.sgn
pca$x[,2] <- pca$x[,2] * y.sgn
pca.df <- data.frame(ID=mat.vals$ID,PC1=pca$x[,1],PC2=pca$x[,2]) %>%
  arrange(PC1)

AZ.df <- pca.df %>% filter(ID %in% c('A','Z'))

### Plot quasispecies projected onto PC1/PC2 plane
library(ggrepel)
pca.df %>%
  mutate(Type=substr(ID,1,3)) %>%
  ggplot() +
  geom_label(aes(x=PC1,y=PC2,label=ID,col=ID,fontface='bold'),
                 data=AZ.df) +
  geom_point(aes(x=PC1,y=PC2),size=2) +
  geom_point(aes(x=PC1,y=PC2,col=ID),size=2,data=AZ.df) +
  xlim(range(pca$x[,1])*1.05) +
  ylim(range(pca$x[,2])*1.1) +
  coord_fixed(ratio = 1) +
```

```r
  labs(x=pca.lbls[1],y=pca.lbls[2],title='PCA coordinates') +
  theme_bw() +
  theme(legend.position='none') +
  theme(panel.background = element_rect(fill='#f3fafb'))

### The AM-to-ZM path
### Fit a parabola by linear regression
lm.sq <- lm(PC2~PC1+I(PC1^2),data=pca.df)
lm.sm <- summary(lm.sq)
cf.lm <- round(lm.sq$coefficients,4)
names(cf.lm) <- c('b0','b1','b2')

p.vals <- signif(lm.sm$coefficients[,4],3)
names(p.vals) <- c('b0','b1','b2')

lm.sm$coefficients %>%
  knitr::kable(digits=4,caption='Fitted parabola by linear regression.')

### Fit a cubic polynomial
lm.cub <- lm(PC2~poly(PC1,3,raw=TRUE),data=pca.df)
lm.sm.cub <- summary(lm.cub)
lm.sm.cub

### Plot PCA scatterplot with fitted parabola
# Data to draw paràbola
x <- seq(min(pca.df$PC1),max(pca.df$PC1),by=0.1)
lm.sq <- data.frame(x=x, y=cf.lm[1]+cf.lm[2]*x+cf.lm[3]*x^2)
# appex coordinates
v.x <- -cf.lm[2]/(2*cf.lm[3])
v.y <- cf.lm[1]+cf.lm[2]*v.x+cf.lm[3]*v.x^2

### Scatterplot of the simulated quasispecies structures on the PC1/PC2 plane,
### with the two bracketing quasispecies A and Z.
### Red curve: parabola fitted by linear regression.
### Black dot: parabola apex
P <- pca.df %>%
  ggplot() +
  geom_line(aes(x=x,y=y),col='maroon',lwd=1,data=lm.sq) +
  #geom_smooth(aes(x=PC1,y=PC2),method='loess',se=FALSE,lwd=1) +
  geom_point(aes(x=PC1,y=PC2,col=ID),size=2) +
  annotate('point',x=v.x,y=v.y,size=2) +
  geom_vline(aes(xintercept=v.x),lty=4,col='gray') +
  annotate('point',x=v.x,y=v.y,size=2) +
  geom_text_repel(aes(x=PC1,y=PC2,label=ID,col=ID,fontface='bold'),
                  data=AZ.df) +
  #geom_text_repel(aes(x=PC1,y=PC2,label=ID,col=ID),fontface='bold') +
  coord_fixed(ratio = 1) +
```

```r
  xlim(range(pca.df$PC1)*1.05) +
  labs(x='PC1 (92.8%)',y='PC2 (5.6%)',title='PCA coordinates with limiting models') +
  theme_bw() +
  theme(legend.position='none') +
  theme(panel.background = element_rect(fill='#f3fafb'))
P

### Identify quasispecies nearest to the appex
Idx.QsX <- which.min(abs(pca.df$PC1-v.x))
Apex.ID <- mat.vals$ID[Idx.QsX]
mat.vals %>% dplyr::slice(Idx.QsX) %>%
  knitr::kable(digits=4,caption='Quasispecies on parabola apex.')

###  PC1/PC2 coordinates for AM and ZM
AZM <- t(data.matrix(AM.ZM[,-1]))
AZMcs <- (AZM-pca$center)/pca$scale
colnames(AZMcs) <- c('AM','ZM')
AM.ZM.pca <- data.frame(t(t(pca$rotation[,1:2]) %*% AZMcs)) %>%
   rownames_to_column(var='ID')
AM.ZM.pca [,'PC1'] <- AM.ZM.pca [,'PC1'] * x.sgn
AM.ZM.pca [,'PC2'] <- AM.ZM.pca [,'PC2'] * y.sgn
Mpca.df <- rbind(AM.ZM.pca[1,],pca.df,AM.ZM.pca[2,])

### PCA-2D-base linear distance from A
PC.d2A.ln <- as.matrix(dist(Mpca.df[,-1]))
rownames(PC.d2A.ln) <- colnames(PC.d2A.ln) <- Mpca.df$ID
PC.d2A.ln <- as.matrix(dist(Mpca.df[,-1]))[-1,1]
PC.d2A.ln <- data.frame(ID=Mpca.df$ID[-1],
                        PC.d2A.ln=PC.d2A.ln,
                        PC.NdA.ln=PC.d2A.ln/max(PC.d2A.ln))
PC.d2A.ln %>%
  arrange(PC.NdA.ln) %>%
  knitr::kable(digits=5,
               caption='PCA-2D-based linear distance from limiting model A')

### PCA-2D parabola arc length from A by rectification
x <- seq(from=min(pca.df$PC1),to=max(pca.df$PC1),by=0.01)
x <- sort(unique(c(pca.df$PC1,x)))
y <- cf.lm[1]+cf.lm[2]*x+cf.lm[3]*x^2

dh <- sapply(1:(length(x)-1),function(i)
           sqrt((x[i+1]-x[i])^2+(y[i+1]-y[i])^2))
idx <- which(x %in% pca.df$PC1)[-1]
d2A.h <- cumsum(dh)[idx-1]

arc.parab <- function(x0,x1,a,b,c)
{ I.parab <- function(x,a,b,c) {
```

```
     A <- 2*c*x+b
    (A*sqrt(1+A^2) + log(abs(A+sqrt(1+A^2)))) / (4*c) }
  I.parab(x1,a,b,c)-I.parab(x0,a,b,c)
}

### PCA-2D parabola analytic arc length.
AM.PC1 <- AM.ZM.pca[1,2]
d2A.I <- sapply(2:nrow(Mpca.df), function(i)
   arc.parab(AM.PC1,Mpca.df$PC1[i],cf.lm[1],cf.lm[2],cf.lm[3]))


PC.d2A <- data.frame(ID=PC.d2A.ln$ID,
                     PC.d2A.I=d2A.I) %>%
  mutate(PC.NdA.I=PC.d2A.I/max(PC.d2A.I))

PC.d2A %>%
  arrange(PC.NdA.I) %>%
  knitr::kable(digits=5,
               caption='PCA-2D-based parabola arc length from limiting model A')

PC.d2A %>%
  left_join(PC.d2A.ln,by='ID') %>%
  select(ID,PC.NdA.I,PC.NdA.ln) %>%
  arrange(PC.NdA.I) %>%
  knitr::kable(digits=4,
              caption='PC-based distance from AM. ln: linear distance; I: parabola arc length.'

### Full dimension distance from A
dtm <- mat.vals %>% column_to_rownames(var='ID') %>%
  data.matrix()
dtm <- rbind(AM=AM,dtm,ZM=ZM)

dtm <- scale(dtm)
D <- as.matrix(dist(dtm))
d2A <- D[1,-1]
N.d2A <- d2A/D['AM','ZM']

d2A.df <- data.frame(ID=names(d2A),FD.d2A=d2A,FD.NdA=N.d2A) %>%
   left_join(mat.vals %>% select(ID,I3),by='ID')
d2A.df %>% select(-I3) %>%
  arrange(FD.NdA) %>%
  knitr::kable(digits=4,caption='Full dimension distance from AM')

### Quasispecies maturity scores
d2A.df <- d2A.df %>%
  select(ID,FD.NdA,I3) %>%
  left_join(PC.d2A %>% select(ID,PC.NdA.I),by='ID') %>%
```

```r
  select(ID,I3,FD.NdA,PC.NdA.I) %>%
  arrange(PC.NdA.I) %>%
  mutate(across(where(is.numeric),~replace(.,is.na(.),1)))
d2A.df %>%
  knitr::kable(digits=4,
               caption='Quasispecies maturity scores')

### Plot maturity scores
d2A.df %>%
  mutate(Idx=1:nrow(d2A.df)) %>%
  pivot_longer(-c(Idx,ID),names_to='Metric',values_to='Score') %>%
  mutate(Metric=factor(Metric,levels=c('I3','FD.NdA','PC.NdA.I'))) %>%
  ggplot() +
  geom_line(aes(x=Idx,y=Score,group=Metric,col=Metric),lwd=0.8) +
  #geom_abline(aes(intercept=-1/16,slope=1/16),lty=4,lwd=0.4) +
  labs(x='Quasispecies index',y='Score',title='Maturity scores')


### Evolution of quasispecies structure indicators.

### Quasispecies indicators and maturity scores
### Quasispecies sorted by NdA values
qs.dt <- mat.vals %>%
  left_join(d2A.df %>% select(ID,PC.NdA.I),by='ID') %>%
  arrange(PC.NdA.I) %>%
  relocate(PC.NdA.I,.after='ID')
qs.dt %>%
  dplyr::rename(NdA=PC.NdA.I) %>%
  mutate(across(where(is.numeric),~replace(.,is.na(.),0))) %>%
  arrange(NdA) %>%
  knitr::kable(digits=4,
               caption="Quasispecies structure indicators and maturity score.")

### Nearest Qs index to the crossing of Master with RLEinf
idx.cross <- which.min(abs(qs.dt$Master-qs.dt$RLEinf))
cross.ID <- qs.dt$ID[idx.cross]

### Appex quasispecies index and values
Apex.idx <- which(qs.dt$ID==Apex.ID)
apex.NdA <- (qs.dt %>% filter(ID==Apex.ID))$PC.NdA.I
apex.x <- which(qs.dt$NdA==apex.NdA)

### Feature labels
### Feature labels
fnms <- colnames(qs.dt)[-(1:2)]
lbl <- data.frame(x=1:10,
                  Lbl=fnms) %>%
```

```
  mutate(y=as.numeric(qs.dt[cbind(x,3:12)]))

### NdA corresponding to the crossing of Master with RLEinf
cross.df <- qs.dt %>%
  select(PC.NdA.I,Master,RLEinf) %>%
  mutate(Dif=Master-RLEinf)
cross.y <- approx(x=cross.df$Dif,y=cross.df$PC.NdA.I,xout=0)$y
cross.p <- approx(x=cross.df$PC.NdA.I,y=cross.df$Master,xout=cross.y)

### Plot: Quasispecies structure indicators versus maturity as NdA.
### Dash-dot red line: parabola apex. Black dot and dash-dot gray line:
### Crossing of Master with RLEinf at N.d2A = 0.462, with Master = 0.221"

lbl2 <- lbl %>%
  mutate(NdA=qs.dt$PC.NdA.I[1:10])

qs.dt %>%
  dplyr::rename(NdA=PC.NdA.I) %>%
  #filter(ID!='A') %>%
  select(-ID) %>%
  pivot_longer(-NdA,names_to='Feat',values_to='Vals') %>%
  ggplot() +
  geom_line(aes(x=NdA,y=Vals,group=Feat,col=Feat),lwd=1.2) +
  geom_vline(aes(xintercept=apex.NdA),lty=4,col='maroon',lwd=1) +
  geom_vline(aes(xintercept=cross.p$x),lty=4,col='gray',lwd=0.8) +
  geom_label_repel(aes(x=NdA,y=y,label=Lbl,col=Lbl),fontface='bold',
                   data=lbl2) +
  annotate('point',x=cross.p$x,y=cross.p$y,size=3,col='black') +
  labs(x='NdA',y='Feature value',
       title='Quasispecies sorted by NdA') +
  theme_bw() +
  theme(legend.position='right')
```

### Session info

R version 4.3.3 (2024-02-29 ucrt) Platform: x86_64-w64-mingw32/x64 (64-bit) Running under: Windows 11 x64 (build 22631)

Matrix products: default

locale: [1] LC_COLLATE=Catalan_Spain.utf8 LC_CTYPE=Catalan_Spain.utf8
[3] LC_MONETARY=Catalan_Spain.utf8 LC_NUMERIC=C
[5] LC_TIME=Catalan_Spain.utf8

time zone: Europe/Madrid tzcode source: internal

attached base packages: [1] stats4 stats graphics grDevices utils datasets methods
[8] base

other attached packages: [1] ggrepel_0.9.6 gridExtra_2.3 lubridate_1.9.4

[4] forcats_1.0.0 stringr_1.5.1 dplyr_1.1.4
[7] purrr_1.0.2 readr_2.1.5 tidyr_1.3.1
[10] tibble_3.2.1 ggplot2_4.0.1 tidyverse_2.0.0
[13] Biostrings_2.70.3 GenomeInfoDb_1.38.8 XVector_0.42.0
[16] IRanges_2.36.0 S4Vectors_0.40.2 BiocGenerics_0.48.1

loaded via a namespace (and not attached): [1] utf8_1.2.4 generics_0.1.3 bitops_1.0-9
[4] stringi_1.8.4 hms_1.1.3 digest_0.6.37
[7] magrittr_2.0.3 timechange_0.3.0 evaluate_1.0.1
[10] grid_4.3.3 RColorBrewer_1.1-3 fastmap_1.2.0
[13] tinytex_0.54 fansi_1.0.6 scales_1.4.0
[16] cli_3.6.3 rlang_1.1.4 crayon_1.5.3
[19] withr_3.0.2 yaml_2.3.10 tools_4.3.3
[22] tzdb_0.4.0 GenomeInfoDbData_1.2.11 vctrs_0.6.5
[25] R6_2.5.1 lifecycle_1.0.4 zlibbioc_1.48.2
[28] pkgconfig_2.0.3 pillar_1.9.0 gtable_0.3.6
[31] Rcpp_1.0.13-1 glue_1.8.0 xfun_0.49
[34] tidyselect_1.2.1 rstudioapi_0.17.1 knitr_1.49
[37] dichromat_2.0-0.1 farver_2.1.2 htmltools_0.5.8.1
[40] labeling_0.4.3 rmarkdown_2.29 compiler_4.3.3
[43] S7_0.2.0 RCurl_1.98-1.16
