# Supplementary material for: Peaked-to-flat transition in quasispecies structure evolution
Source: Virus Evol. 2026 Apr 14;12(1):veag024. doi: 10.1093/ve/veag024 (PMC13137331; doi:10.1093/ve/veag024)
Supplement: Supplementary_material_veag024 [file supplementary_material_veag024.zip › VEVOLU-2025-229_R2_Supplementary_2_Statistics_veag024.pdf]

Peaked-to-Flat Transition in Quasispecies Structure Evolution

Supplementary material - Statistical analysis

Josep Gregori Sergi Colomer-Castell Carolina Campos Marta Ibañez-Lligoña
Damir García-Cehic Alvaro González-Camuesco Maria F. Cortese David Tabernero
Mar Riveiro-Barciela Maria Buti Ariadna Rando-Segura Roser Ferrer
Tomás Pumarola Cristina Andrés Andrés Antón Francisco Rodríguez-Frías
Josep Quer

2026-04-06

Abstract

Previous studies based on clinical data from HCV and HEV infections revealed a deterministic evolution of quasispecies structure, irrespective of haplotype identities, toward a flat-like landscape, characterized by the absence of dominance and high evenness, combined with high haplotype synonymy. Here, two idealized limiting quasispecies states, A and Z, are defined, and it is shown that the A-to-Z evolution describes a parabolic trajectory between these two states. The initial phase is dominated by increasing genetic diversity, whereas the subsequent phase is driven primarily by increasing evenness in the haplotype distribution. This evolutionary progression confers a broad domain within the genetic space, resulting in increased fitness and resilience, accompanied by a diminished response to antiviral therapies and multiple low-cost escape routes. Finally, a normalized quasispecies maturity score is proposed to position a given quasispecies along this evolutionary trajectory. This conceptual framework helps to account for the challenges in treating advanced chronic infections, in which therapeutic failure frequently occurs in the absence of resistance-associated mutations.

Introduction

This supplementary document presents a comprehensive statistical analysis of changes in quasispecies structure indicators across transitions between consecutive fibrosis stages. The analyses include p-values, effect sizes, boxplots, and ROC curves. In addition to examining central tendency, the degree of overlap between populations at different fibrosis stages is also assessed. Furthermore, the study explores haplotype synonymy, consistent with previous HEV and HCV quasispecies analyses (Gregori et al. 2022; Gregori, Colomer-Castell, et al. 2024), and its relationship with the maturation state of the quasispecies.

Table S1: Number of samples per fibrosis level and genomic amplicon

Ampl	F1	F2	F3	F4	Total
NS3	4	6	18	39	67
NS5A	4	6	18	40	68
NS5B1	3	6	17	38	64
NS5B2	3	5	16	40	64
Total	14	23	69	157	263

1 Statistic comparisons and tests

1.1 HCV fibrosis dataset

In this document we inspect the likely correlation between liver fibrosis levels and different indicators of quasispecies genetic structure, including quasispecies maturity and haplotype synonymy. The dataset consists in the following:

- Number of patients with fibrosis data 69
- Total number of amplicons 263
- Number of samples per amplicon and fibrosis level listed in Table S1.

All quasispecies studied derive from HCV chronically infected patients with failed antiviral treatments, quantified liver fibrosis scores (F1-F4), and medium-to-high viremia ($> 10^4$ IU/mL), confirming functional viral populations.

While infection duration shows high correlation with fibrosis severity, multiple uncontrolled confounders, not always recorded in clinical histories, impact both quasispecies diversity and liver damage progression. These include:

- Viral load trajectory over infection course, where sustained higher viral loads imply an increased rate of accumulated substitutions.
- Prior treatment history includes the type (mutagenic versus direct-acting antivirals), duration, and dosage. Mutagenic drugs accelerate quasispecies diversification, whereas unsuccessful DAA treatments select subpopulations with reduced treatment response, transiently reducing quasispecies diversity.
- Targeted genomic regions: NS3/4A, NS5A, NS5B, or combinations threof.
- Host factors: immune status, steatosis, co-infections, metabolic syndrome.
- Time since treatment cessation or discontinuation, representing the quasispecies recovery period.

This real-world clinical heterogeneity underscores the value of reference-free maturity metrics, which quantify quasispecies evolution independent of incomplete covariates. This is evident in cases where F2 patients exhibit maturity scores typical of advanced stages, or F4 patients show unexpectedly moderate maturity scores. We propose these scores serve as surrogates for quasispecies fitness and treatment resilience, consistent with "survival-of-the-flattest" theory, where flatter (higher-entropy) distributions confer robustness and adaptive advantage under therapeutic pressure. Haplotype synonymy plays and important role in keeping functionality and viremia, despite the high genetic diversity. Critically, this high-entropy functional state (compatible with sustained high viremia) is distinct from error catastrophe, where quasispecies collapse into non-functional diversity.

1.2 Quasspecies representation by rank-abundance cumulative distributions

The quasispecies distributional structure can be visualized using the rank-abundance cumulative distribution (RACD), obtained by plotting cumulative frequencies of haplotypes sorted by decreasing abundance. A key feature of this representation is the height of the curve's elbow relative to the 45° diagonal, which represents the uniform distribution (State Z: equal frequencies across all haplotypes). Distributions closer to State A (single dominant master) form a sharp-elbowed curve hugging the axes, while flatter distributions approach the diagonal.

Figure S1 shows the averaged RACD curve by fibrosis stage. Figure S2 depicts the cumulative distributions of all quasispecies in the dataset, separated by fibrosis score but excluding F1. The tendency to lower and flatter distributions in patients with advanced liver damage is evident, however a significant overlap exists.

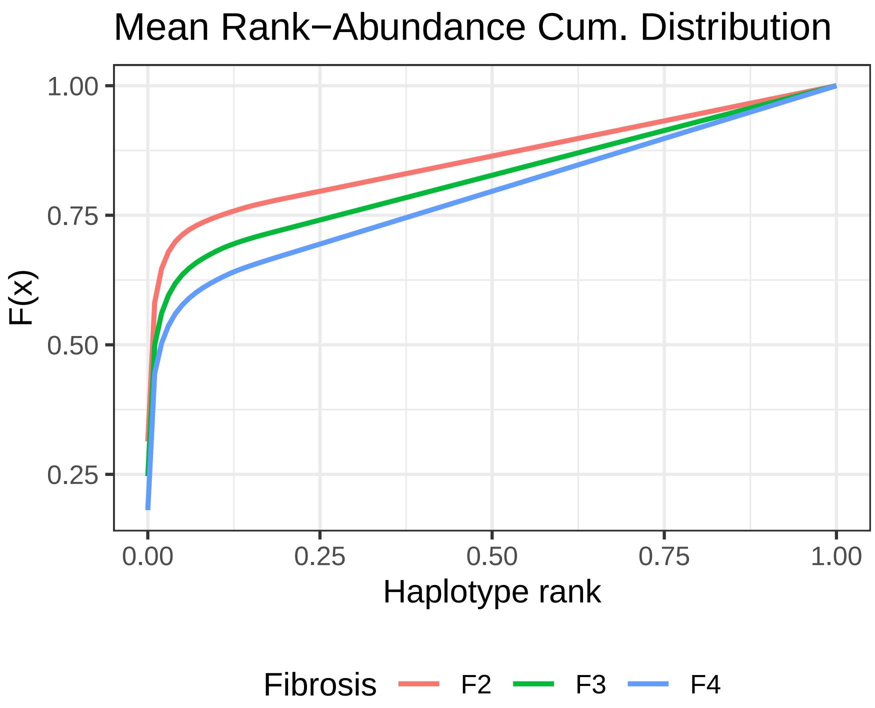

Figure S1: Mean haplotype rank-abundance cumulative distribution for each fibrosis stage in the HCV cohort.

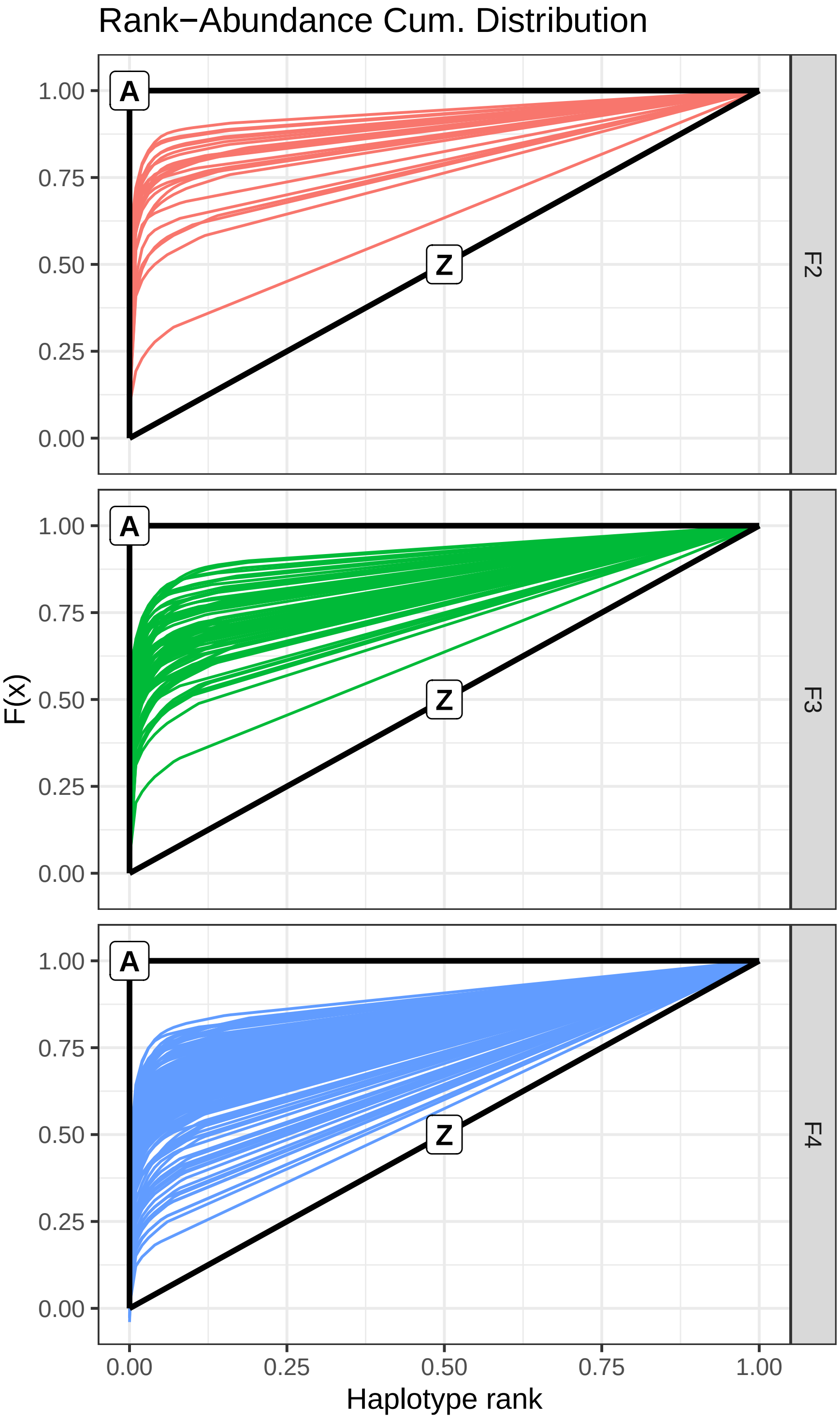

Figure S2: Haplotype rank-abundance cumulative distribution. HCV cohort.

1.3 Quasspecies representation by Relative Logarithmic Evenness profiles

A complementary representation to the RACD is the relative logarithmic evenness profile (RLE) for q values between $q = 0$ and $q = 3$. Beyond $q = 3$ the profile is highly asymptotic untill $q = \infty$. Low profiles are indcative of peaed dstrbutions, whereas high profiles are representative of more even flatter distributions. Figure S3 shows the averaged profile by fibrosis stage. Figure S4 show the RLE profiles of all quasispecies in the HCV dataset, separated by fibrosis score with F1 samples excluded.

As with the RACD representation we may observe the tendency to higher profiles in patients with advanced liver damage, but with a significant overlap between fibrosis stages.

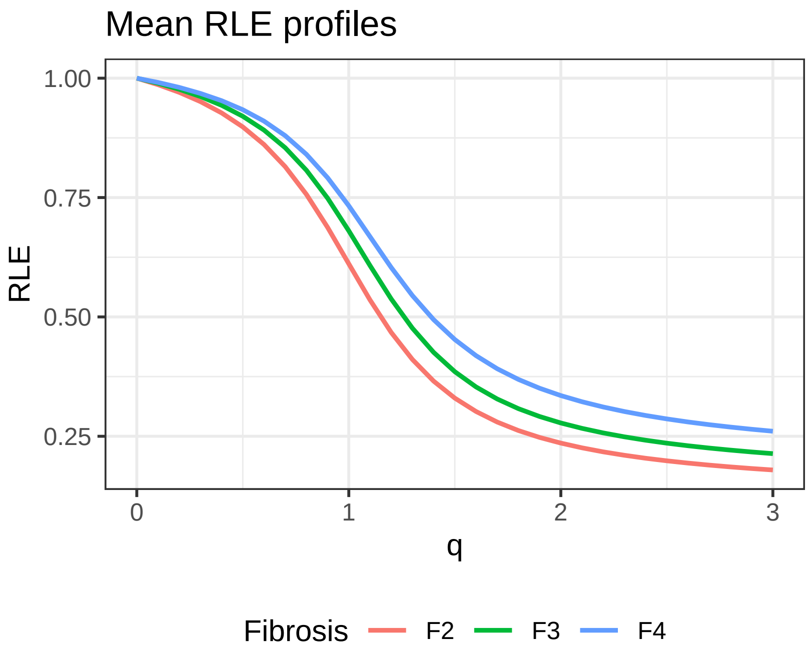

Figure S3: Averaged RLE profiles by fibrosis stage. HCV cohort.

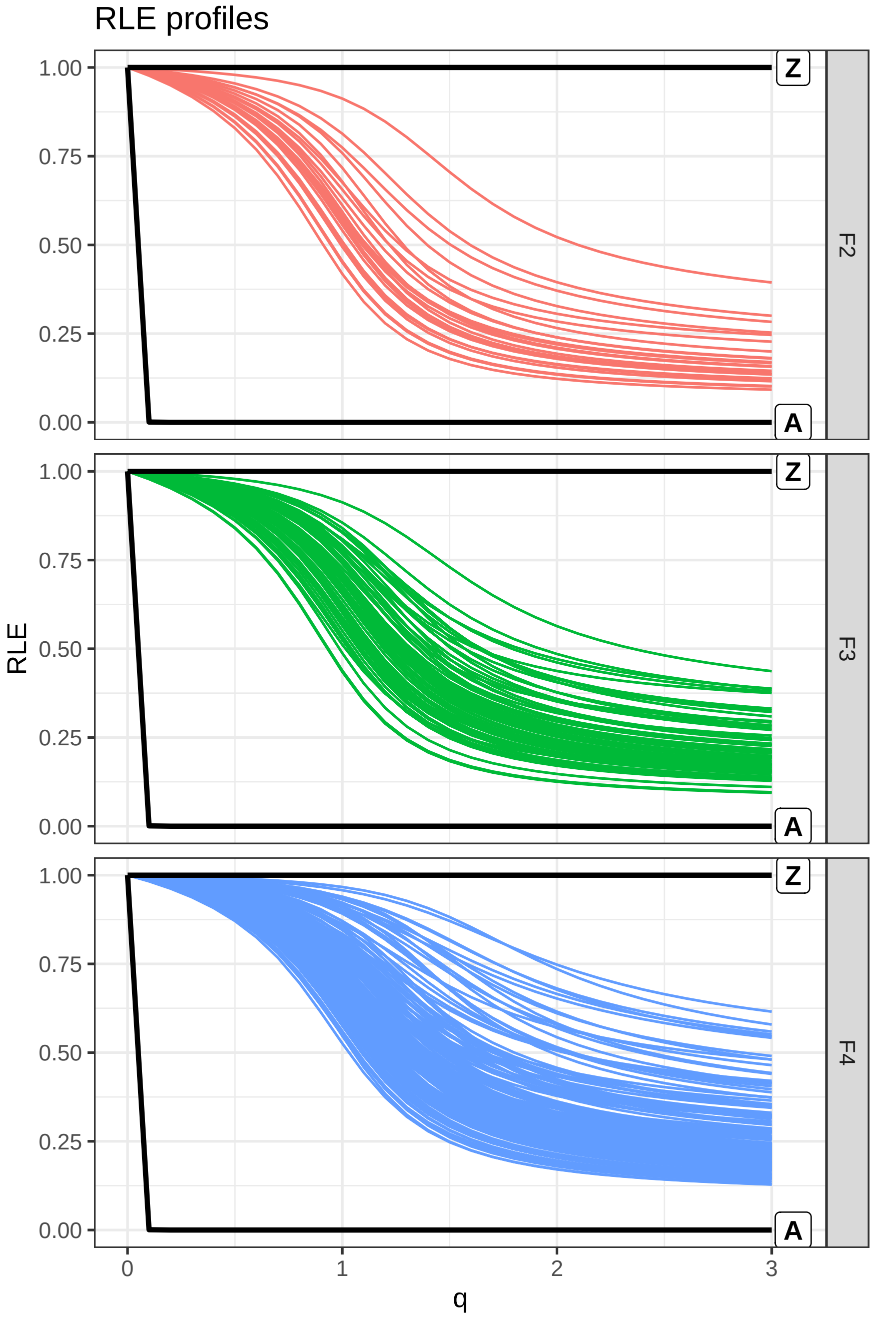

Figure S4: Relative logarithmic evenness profiles. HCV cohort.

1.4 Quasispecies structure indicators

An expanded set of quasispecies structure indicators, relative to those used in the main text, and developed in recent studies (Gregori, Colomer-Castell, et al. 2024; Gregori, Ibañez-Lligoña, et al. 2024; Gregori et al. 2025), is considered in this statistical analysis to assess their association with liver damage as measured by fibrosis scores. Table S2 lists these indicators together with their defined values in the two limiting states, A and Z.

Table S2: Quasispecies structure indicators and corresponding values in the two idealized limiting states A and Z.

Feature	Description	State A	State Z
TopN	Fraction of reads for top N hpl.	1	0
Master	Dominant haplotype frequency	1	0
Rare1	Fraction of reads for hpl $\leq 1\%$	0	1
Rare2	Fraction of reads for hpl $\leq 0.1\%$	0	1
Singl	Fraction of reads for singletons	0	1
RLE_1	Relative logarithmic evenness at $q = 1$	0	1
RLE_2	Relative logarithmic evenness at $q = 2$	0	1
RLE_3	Relative logarithmic evenness at $q = 3$	0	1
RLE_∞	Relative logarithmic evenness at $q = \infty$	0	1
I_3	Normalized RLE integral for $q = 0:3$	0	1
R_k	Evenness of top k haplotypes (k= 5, 10)	0	1
$TopNR$	Ratio of frequencies TopN to Master	0	1

All these indicators may be calculated directly from the vector of haplotype ranked abundances $\hat{\mathbf{P}} = (n_1/N, n_2/N, \cdots, n_H/N) = (\hat{p_1}, \hat{p_2}, \cdots, \hat{p_H})$, with n_i haplotype read counts, H number of haplotypes, N the total number of reads, and $\hat{p_1} \geq \hat{p_2} \geq \cdots \geq \hat{p_H}$.

- Quasispecies fitness fractions:

$$TopN = \sum_{k=1}^{N} \hat{p}_i$$

$$Master = max(\hat{p}_i) = \hat{p}_1$$

$$Rare1 = \sum_{i}^{H} \hat{p}_i \; I(\hat{p}_i \leq 0.01)$$

$$Rare2 = \sum_{i}^{H} \hat{p}_i \; I(\hat{p}_i \leq 0.001)$$

$$Singl = \frac{1}{\sum_i^H n_i} \sum_{i}^{H} n_i \; I(n_i = 1)$$

where $I(.)$ is the indicator function, yielding 0 or 1 depending on whether the expression inside the parentheses is false or true.

- Relative logarithmic evenness indicators:

$$RLE_q = \frac{log(D(\hat{\mathbf{P}}, q))}{log(H)}$$

where $D(q, \hat{\mathbf{P}})$ is the Hill number of order q, with $q = 1, 2, 3, \infty$.

$$D(\hat{\mathbf{P}}, q) = \left(\sum_{i=1}^{H} \hat{p}_i^q \right)^{1/(1-q)}$$

- Top haplotypes evenness indicators R_k:

$$R_k = \frac{k \cdot \hat{p}_k}{\sum_i^{k-1} \hat{p}_i}$$

the ratio of the k-th ranked haplotype frequency to the mean of the $(k-1)$ top ranked haplotype frequencies

$$TopNR = \frac{1}{p_1} \sum_i^N \hat{p}_i$$

the ratio of the cumulative frequency of the N top ranked haplotypes to the Master haplotype frequency.

The zeroes for *TopN* and *Master*, in State Z, should be interpreted as an infinitesimal frequency ϵ (a positive real number, $\epsilon > 0$ and $\epsilon < 1/n$ for any positive integer n), rather than strictly zero. Biochemically, this corresponds to one to a few (or several) molecules in the context of high viral titre. Similarly, the zeros for *Rare1* and *Singl* in State A represent minor variants (other than the master haplotype) at infinitesimal frequencies. However, we represent ϵ formally as 0.

1.5 Maturity score

A maturity score, *NdA*, is also calculated as the arc length from State A to the quasispecies position, normalised to the total arc length from A to Z, along the cubic polynomial fitted (in the main text) to the HCV quasispecies cloud projected onto the PC1/PC2 plane. This plane is derived from principal components analysis of the covariance matrix of quasispecies structure indicators for the HCV cohort.

1.6 Mann-Whitney U test and statistics

Statistic significance was assessed using the non-parametric Mann-Whitney (MW) test (Mann and Whitney 1947), comparing each indicator between consecutive fibrosis stages: F1-F2, F2-F3, and F3-F4 (Table S3 and Figure S5). Despite differences in amplicon length and functionality among the four sequenced regions -NS3 444bp, NS5A 387bp, NS5B1 396 bp and NS5B2 396bp- they were analyzed equivalently, as if representing a single genomic region. This approximation assumes quasispecies with flat-like fitness landscapes exhibit consistent characteristics genome-wide (See Figure S11), introducing additional variability that may reduce the test power but strengthens the robustness of the observed effects. Each amplicon is characterized by selected structure indicators and labeled by patient fibrosis stage.

The U_1 statistic of the MW test directly relates to the area under the ROC curve (AUC) as a normalized probability measure, the probability of superiority (PS), assessing the probability $P(X > Y)$ that a randomly selected score from the X group exceeds one from the Y group.

$$\text{AUC} = \text{PS} = \frac{U_1}{n_1 n_2}$$

Table S3 shows test results comparing each indicator between two consecutive fibrosis stages. Besides statistical significance from Benjamini-Hochberg multitest-adjusted p-values (Benjamini and Hochberg 1995) the table also includes AUC values. The F2 vs. F1 comparison has been excluded from this table, as no indicator shows statistically significant p-values. Figure S5 plots the p-values of all tests in log-scale.

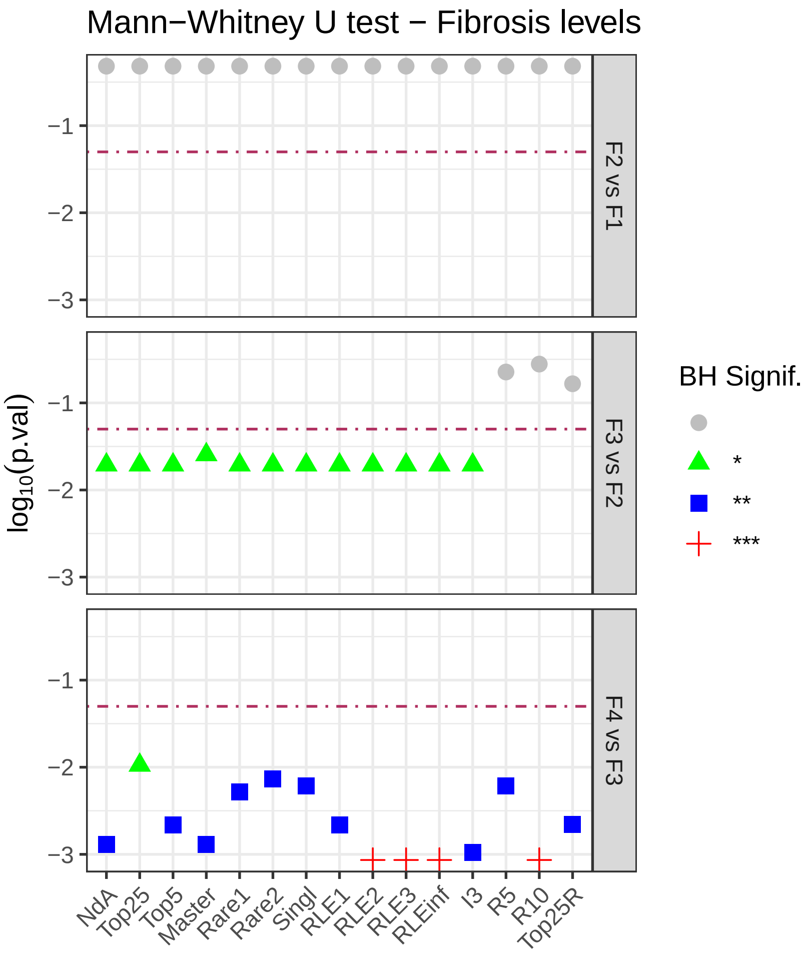

Figure S5: Mann-Whitney U test, Benjamini-Hochberg adjusted p-values in log10 scale. BH Signif: * <0.05, ** <0.01, *** <0.001. Dash-dot red line at 0.05.

Table S3: Mann-Whitney U tests results, Stg2 vs Stg1. Effect-size as the area under the ROC curve (AUC), p-values, Benjamini-Hotchberg adjusted p-values, and statistical significance: * <0.05, ** <0.01, *** <0.001

Stg2	Stg1	Feature	AUC	p.val	BH.adj	Star
F3	F2	NdA	0.6692	7.834e-03	1.939e-02	*
F3	F2	Top25	0.6786	5.358e-03	1.939e-02	*
F3	F2	Top5	0.6534	1.422e-02	1.939e-02	*
F3	F2	Master	0.6434	2.034e-02	2.542e-02	*
F3	F2	Rare1	0.6648	9.301e-03	1.939e-02	*
F3	F2	Rare2	0.6578	1.209e-02	1.939e-02	*
F3	F2	Singl	0.6982	2.318e-03	1.939e-02	*
F3	F2	RLE1	0.6830	4.462e-03	1.939e-02	*
F3	F2	RLE2	0.6591	1.153e-02	1.939e-02	*
F3	F2	RLE3	0.6560	1.297e-02	1.939e-02	*
F3	F2	RLEinf	0.6578	1.209e-02	1.939e-02	*
F3	F2	I3	0.6730	6.743e-03	1.939e-02	*
F3	F2	R5	0.5564	2.111e-01	2.262e-01	
F3	F2	R10	0.5413	2.789e-01	2.789e-01	
F3	F2	Top25R	0.5747	1.437e-01	1.658e-01	
F4	F3	NdA	0.6353	6.056e-04	1.298e-03	**
F4	F3	Top25	0.5964	1.055e-02	1.055e-02	*
F4	F3	Top5	0.6259	1.302e-03	2.170e-03	**
F4	F3	Master	0.6371	5.183e-04	1.296e-03	**
F4	F3	Rare1	0.6115	3.834e-03	5.229e-03	**
F4	F3	Rare2	0.6030	6.887e-03	7.379e-03	**
F4	F3	Singl	0.6069	5.263e-03	6.072e-03	**
F4	F3	RLE1	0.6261	1.274e-03	2.170e-03	**
F4	F3	RLE2	0.6465	2.296e-04	8.609e-04	***
F4	F3	RLE3	0.6483	1.943e-04	8.609e-04	***
F4	F3	RLEinf	0.6482	1.959e-04	8.609e-04	***
F4	F3	I3	0.6417	3.484e-04	1.045e-03	**
F4	F3	R5	0.6072	5.163e-03	6.072e-03	**
F4	F3	R10	0.6506	1.573e-04	8.609e-04	***
F4	F3	Top25R	0.6244	1.462e-03	2.193e-03	**

Most quasispecies structure indicators significantly distinguish between fibrosis levels F2 and F3, and F3 and F4, but none is statistically significant when comparing F2 to F1. Notably, p-values for all indicators are lower for the F4 vs. F3 comparison than for F3 vs. F2. These p-values should be interpreted, however, with consideration of the unbalanced sample sizes, with fewer amplicons for lower stages, particularly F1 (14 amplicons), F2 (23), F3 (69), and F4 (157). Of note, the three non-significant indicators between F3 and F2 correspond to top-haplotype evenness measures, while all indicators are statistically significant when distinguishing F4 from F3. Note also that the indicators showing higher statistic significance in F4 vs. F3 are quasispecies evenness (RLE) indicators.

1.7 Effect size

Beyond statistical significance indicated by p-values, effect size measures are essential because they are insensitive to sample size and quantify effect magnitude. While p-values test whether an observed signal exceeds noise, they provide no information about practical importance (Nakagawa and Cuthill 2007; Kelley and Preacher 2012). The AUC (Hanley and McNeil 1982) serves as the natural effect size measure for the MW test. We also report the rank-biserial correlation $R_{RB} = 2 \cdot \text{AUC} - 1$ (Cureton 1956) and the standardized robust nonparametric $\gamma_{0.5}$ (Lötsch and Ultsch 2020; Akinshin 2020), a robust version of Cohen's *d*. See Figures S6 and S7, and Tables S4 and S5.

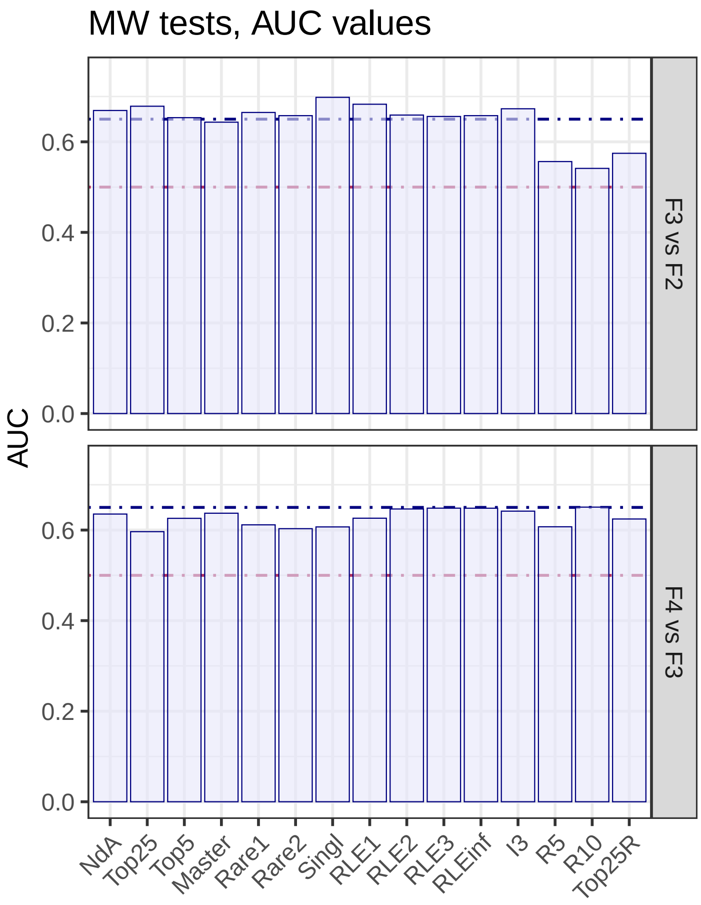

Figure S6: AUC values resulting from the Wicoxon tests. Dash-dot lines at AUC= 0.5 and 0.65

The $\gamma_{0.5}$ statistic, as a standardized effect size measure, more explicitly distinguishes effect magnitudes than the AUC.

Table S4: Robust Cohen's effect size $\gamma_{0.5}$.

Feature	F3 vs F2	F4 vs F3
NdA	0.8347	0.2921
Top25	-1.1516	-0.1326
Top5	-0.6883	-0.3009
Master	-0.5558	-0.4571
Rare1	0.9762	0.2452
Rare2	0.8747	0.2757
Singl	1.1843	0.2022
RLE1	0.9096	0.2417
RLE2	0.6123	0.4093
RLE3	0.6518	0.4657
RLEinf	0.6523	0.4629
I3	0.7319	0.3479
R5	0.2314	0.6218
R10	0.2187	0.4926
Top25R	0.4931	0.4201

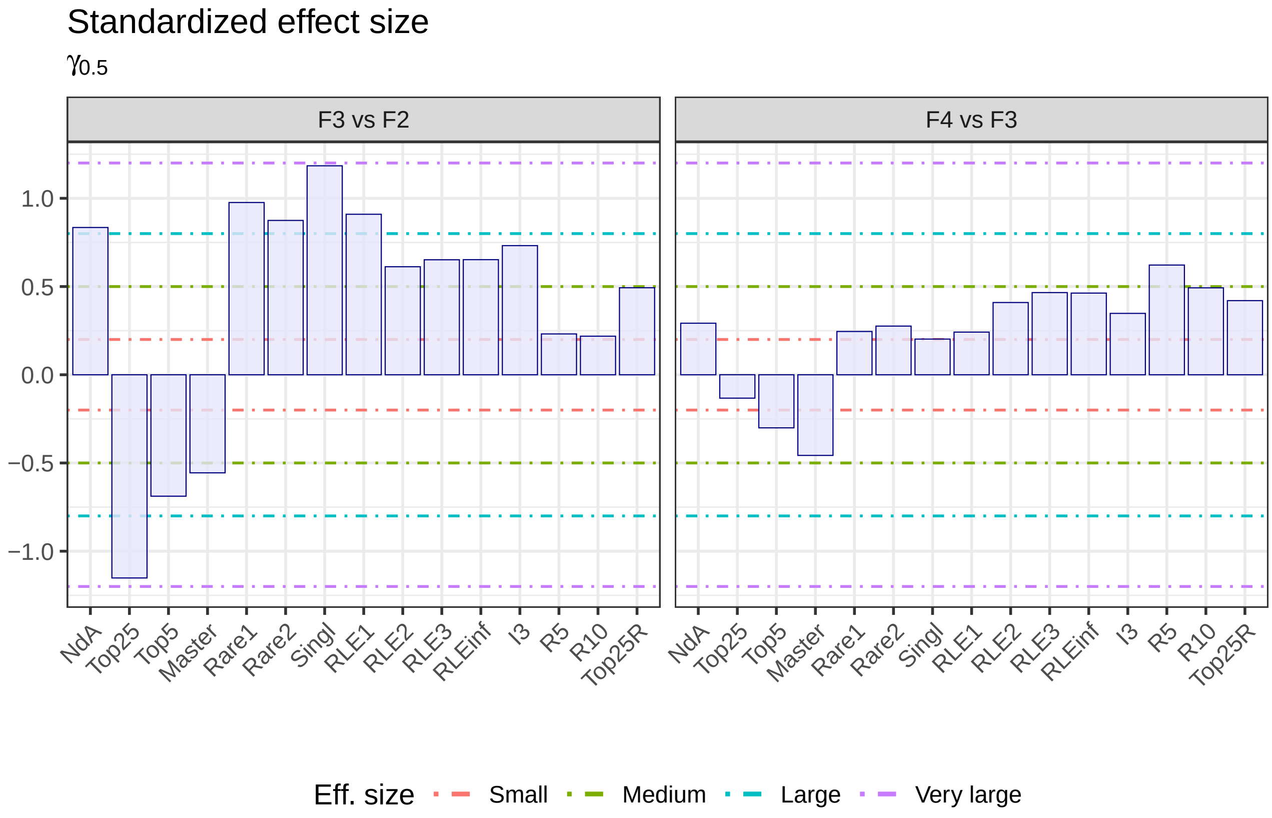

Figure S7: $\gamma_{0.5}$ effect size, with effect magnitude borders. Dash-dot lines at 0.2, 0.5, 0.8, and 1.2.

Table S5: Test results, Benjamini-Hochberg adjusted p-values and effect size metrics.

Test	Feature	BH.adj	Signif	AUC	RRB	Gamma
F3 vs F2	NdA	1.939e-02	*	0.669	0.3380	0.835
F3 vs F2	Top25	1.939e-02	*	0.679	0.3570	-1.150
F3 vs F2	Top5	1.939e-02	*	0.653	0.3070	-0.688
F3 vs F2	Master	2.542e-02	*	0.643	0.2870	-0.556
F3 vs F2	Rare1	1.939e-02	*	0.665	0.3300	0.976
F3 vs F2	Rare2	1.939e-02	*	0.658	0.3160	0.875
F3 vs F2	Singl	1.939e-02	*	0.698	0.3960	1.180
F3 vs F2	RLE1	1.939e-02	*	0.683	0.3660	0.910
F3 vs F2	RLE2	1.939e-02	*	0.659	0.3180	0.612
F3 vs F2	RLE3	1.939e-02	*	0.656	0.3120	0.652
F3 vs F2	RLEinf	1.939e-02	*	0.658	0.3160	0.652
F3 vs F2	I3	1.939e-02	*	0.673	0.3460	0.732
F3 vs F2	R5	2.262e-01		0.556	0.1130	0.231
F3 vs F2	R10	2.789e-01		0.541	0.0826	0.219
F3 vs F2	Top25R	1.658e-01		0.575	0.1490	0.493
F4 vs F3	NdA	1.298e-03	**	0.635	0.2710	0.292
F4 vs F3	Top25	1.055e-02	*	0.596	0.1930	-0.133
F4 vs F3	Top5	2.170e-03	**	0.626	0.2520	-0.301
F4 vs F3	Master	1.296e-03	**	0.637	0.2740	-0.457
F4 vs F3	Rare1	5.229e-03	**	0.612	0.2230	0.245
F4 vs F3	Rare2	7.379e-03	**	0.603	0.2060	0.276
F4 vs F3	Singl	6.072e-03	**	0.607	0.2140	0.202
F4 vs F3	RLE1	2.170e-03	**	0.626	0.2520	0.242
F4 vs F3	RLE2	8.609e-04	***	0.646	0.2930	0.409
F4 vs F3	RLE3	8.609e-04	***	0.648	0.2970	0.466
F4 vs F3	RLEinf	8.609e-04	***	0.648	0.2960	0.463
F4 vs F3	I3	1.045e-03	**	0.642	0.2830	0.348
F4 vs F3	R5	6.072e-03	**	0.607	0.2140	0.622
F4 vs F3	R10	8.609e-04	***	0.651	0.3010	0.493
F4 vs F3	Top25R	2.193e-03	**	0.624	0.2490	0.420

1.8 Collected metrics of all comparisons

Table S5 lists p-values for all tests, Benjamini-Hochberg multitest-adjusted p-values (Benjamini and Hochberg 1995), and the effect size evaluated as the statistics AUC (Hanley and McNeil 1982), R_{BR} (Cureton 1956), and $\gamma_{0.5}$ (Lötsch and Ultsch 2020; Akinshin 2020). Results for F2 vs. F1 have been excluded from the table.

Note that although p-values are generally smaller (and thus more statistically significant) for the F3–F4 transition, effect sizes show the opposite pattern, the former being strongly influenced by sample size imbalance, the latter unaffected.

Positive $\gamma_{0.5}$ indicates larger indicator values in the left class (i.e. F3 > F2), negative $\gamma_{0.5}$ indicates larger values in the right class (i.e. F3 < F2).

Changes between F2 and F3 exceed those between F3 and F4 for all indicators except the top haplotype evenness measures R5 and R10. Only R5 shows a medium effect in the later transition, while Top25 and

Singl exhibit the largest effects in the F2–F3 transition. The most affected indicators differ between phases: QFF measures associated with diversification dominate the F2–F3 transition, whereas evenness indicators predominate in the F3–F4 transition.

$\gamma_{0.5}$ provides finer resolution than AUC. R5 and R10 are minimally affected in F2–F3 but most changed in F3–F4; Top25 and rare fractions show largest F2–F3 effects but stabilise later. This pattern indicates rapid diversification early on, slowing thereafter as evenness among top haplotypes increases through proliferation of alternative functional variants generated by replication errors in the early phase. However global quasispecies evenness, as captured by RLE indicators, rises most sharply during the F2–F3 transition.

This supports a two-phase model of quasispecies structural evolution: an initial phase driven by increasing diversification and declining top haplotype frequencies, followed by a second phase primarily characterised by rising evenness among top haplotypes.

1.9 Boxplot and ROC curves for selected indicators

Figures S8 to S10 show boxplots of selected indicators by fibrosis stage. Although distributions across stages largely overlap for these indicators, they become increasingly right-skewed at higher fibrosis levels, with medians shifting toward higher values. The ROC curves besides each boxplot show the discriminant capacity of each indicator between fibrosis stages, measured by the area under the ROC curve, the AUC metric (Table S5). F2 vs. F1 comparisons show high distributional overlaps with null discriminant capacity, whereas the F3 vs. F2 and F4 vs. F3 comparisons reveal the expected evolution toward higher quasispecies diversity and maturity as liver damage progresses. Infection duration links increasing quasispecies diversity with fibrosis progression. Additional data may be required to detect changes between F1 and F2, as F1 is the least represented stage in the dataset (4 patients, 14 amplicons). Other reasons may be considered: the distinction between F1 and F2 may be unreliable, or the time interval between these stages may be insufficient for detectable diversification to occur, or a combination of these reasons may apply.

These plots reveal that the differences in indicator medians between F2 and F1, and between F4 and F3, are more similar to each other than those between F3 and F2, indicating that the most profound changes occur between F2 and F3. It may be observed also how the effect sizes are larger for F3 vs. F2 than for F4 vs. F3 (Table S5, and Figure S7). From the meta-analysis and meta-regression of (Thein et al. 2008), the estimated annual probabilities of progression between fibrosis stages are: F0 to F1, 0.11-0.13, F1 to F2, 0.08–0.09; F2 to F3, 0.12; and F3 to F4, 0.11–0.12, corresponding to transition times of 8-9, 11–12, 8–9, and 8–9 years, respectively. Thus, the largest differentiation in quasispecies structure occurs during the F2-to-F3 transition, approximately 20–25 years post-infection.

Transition	Annual probability (Thein et al. 2008)	Estimated years (Thein et al. 2008)
**F0 → F1**	~0.107–0.128	**~8–9 years**
**F1 → F2**	~0.082–0.085	**~11–12 years**
**F2 → F3**	~0.117–0.120	**~8–9 years**
**F3 → F4**	~0.116	**~8–9 years**

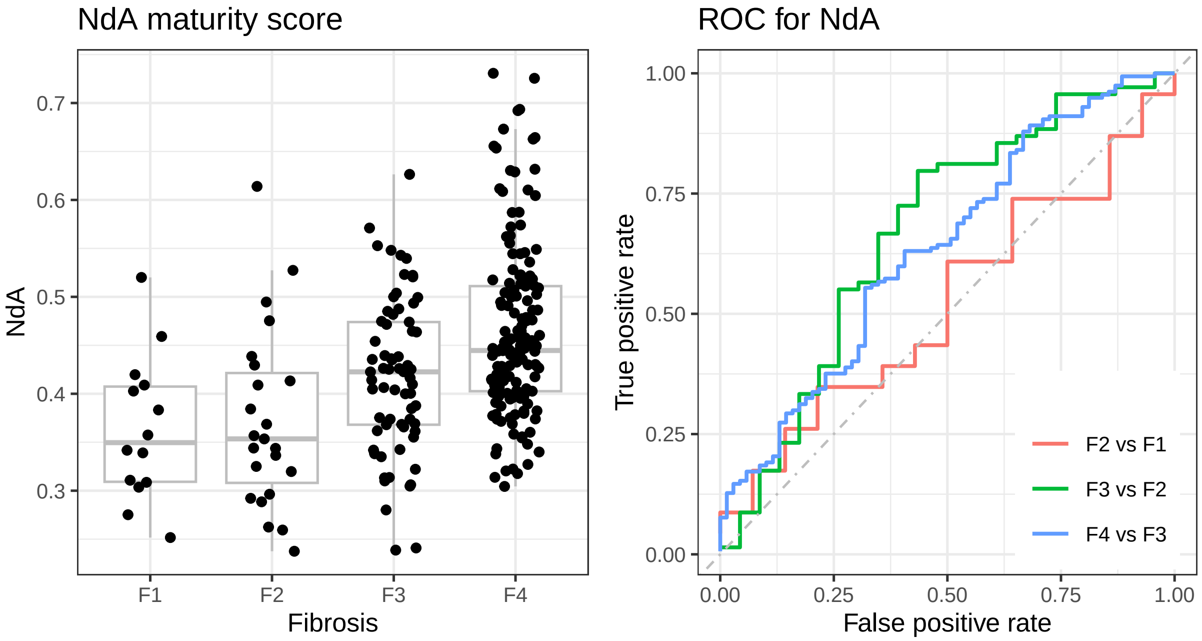

Figure S8: NdA maturity score. Boxplots and ROC curves.

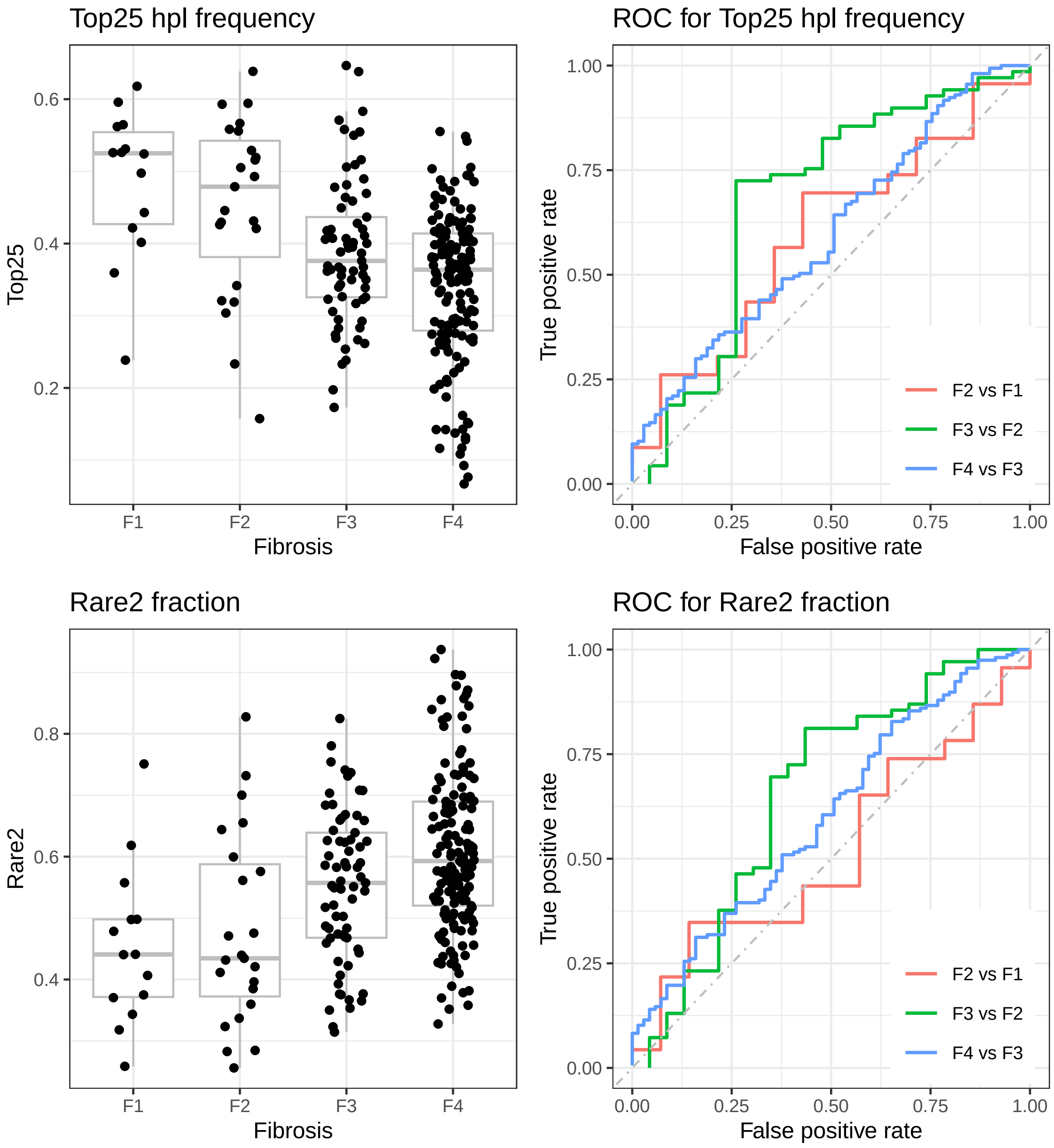

Figure S9: Top: Fraction of reads for top 25 haplotypes.. Bottom: Fraction of reads for Rare2. Boxplots and ROC curves.

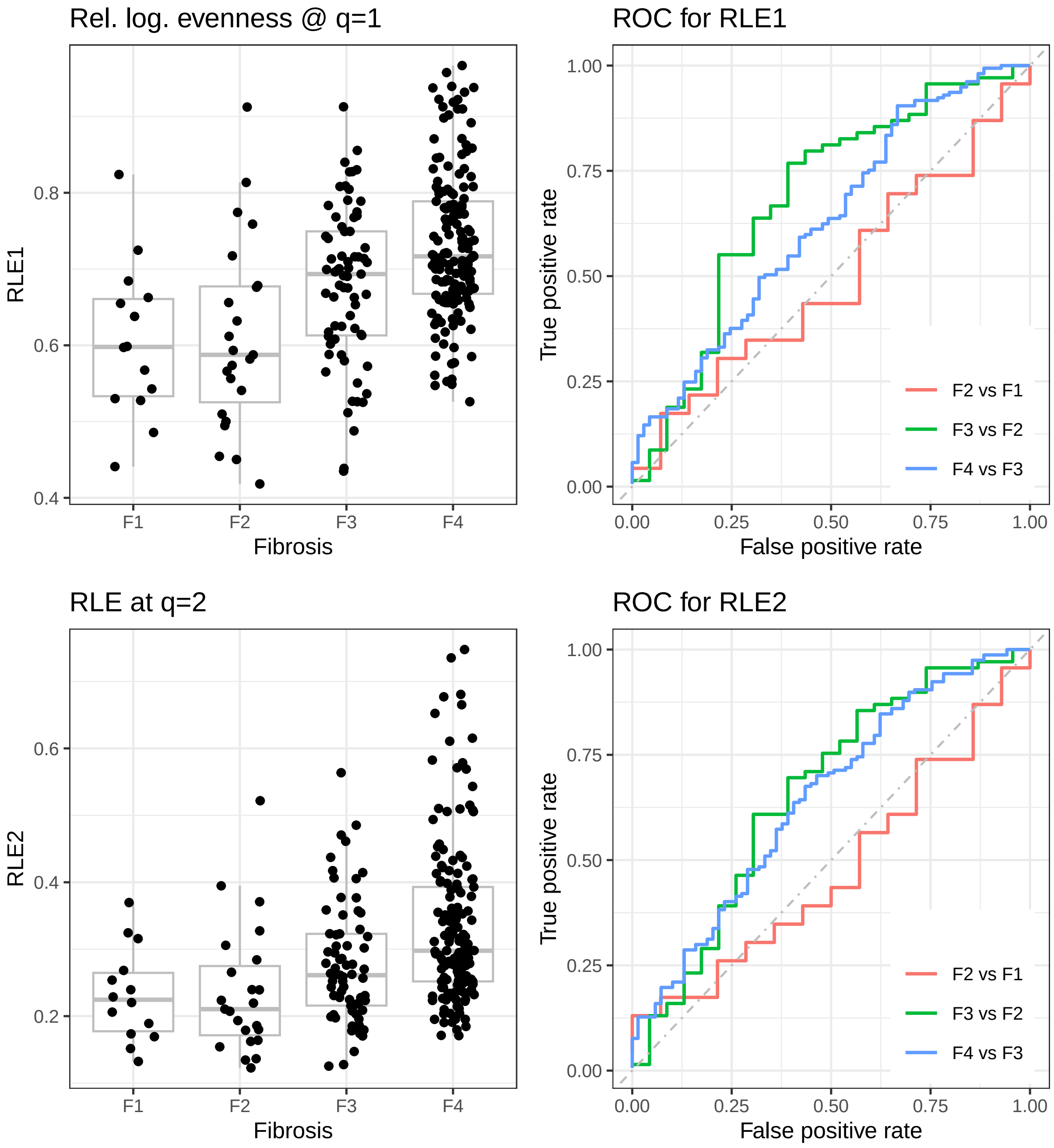

Figure S10: Top: Relative logatithmic evenness at q=1. Bottom: Top: Relative logatithmic evenness at q=2. Boxplots and ROC curves.

1.10 Maturity scores

The spread of NdA values within each fibrosis stage can be better illustrated by plotting ranked NdA against raw NdA values by fibrosis stage, with vertical jitter applied to the points via random displacements in the range (0, 0.1), as in Figure S11. The line in these plots shows the exact vertical position of the corresponding dots, while black triangles represent group medians. This representation distinguishes amplicons, revealing the impact and spread across the four analyzed genomic regions.

The observed overlaps suggest that, beyond the association of the NdA maturity score with fibrosis damage (and indirectly with infection duration), other unknown confounding factors in patients' clinical histories also influence the evolutionary state of quasispecies structure.

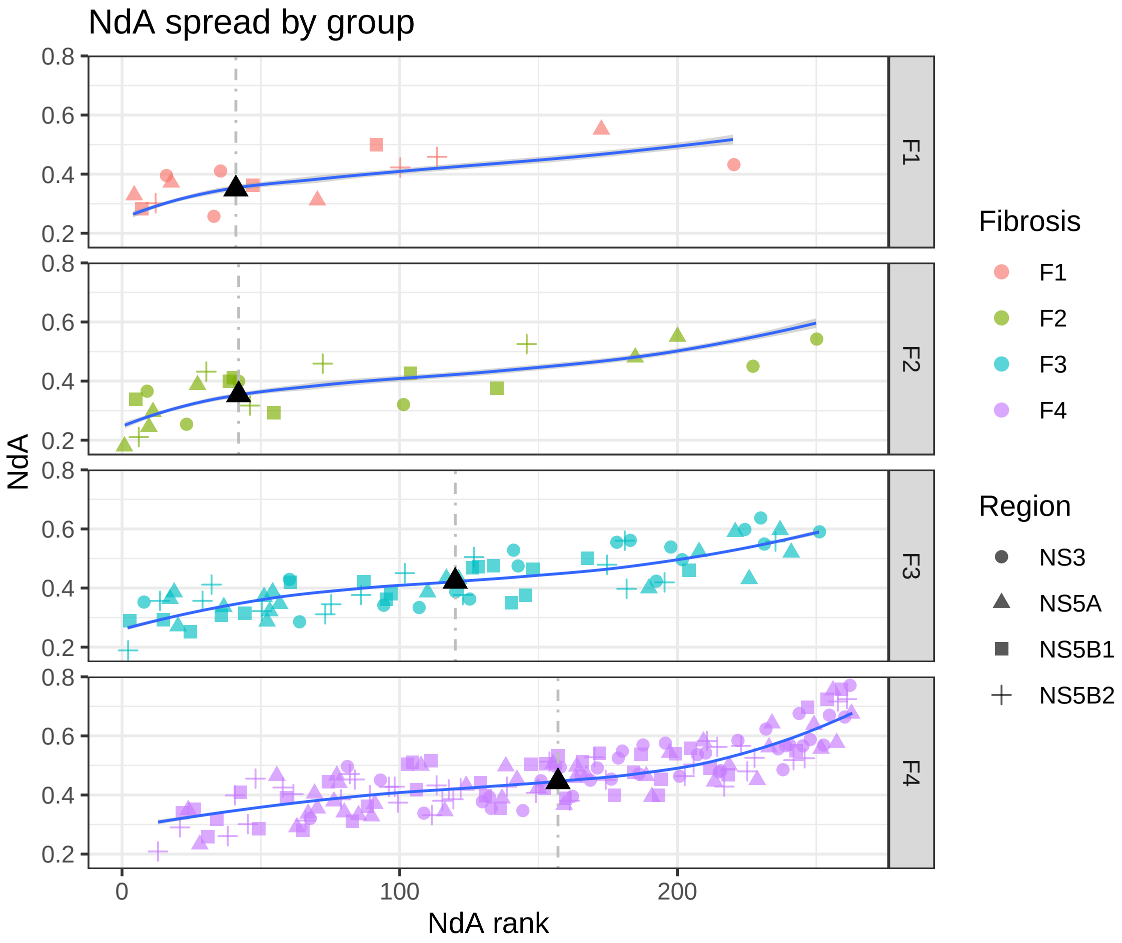

Figure S11: Group overlaps illustrated by ranked versus raw NdA values by fibrosis stage. Dots are jittered vertically by a random value within (0, 0.1). The line shows the exact vertical position of the corresponding dots. Black triangles represent group medians. Shapes of colored dots distinguish amplicons.

The four proposed scores, Y_e for haplotype dominance, and AoC, $I3$ and NdA for evenness and maturity, are highly correlated. Y_e and AoC, both derived from the RACD representation, are inversely correlated ($R = -0.995$), whereas $I3$ obtained from the RLE profile, and NdA, calculated from the set of quasispecies structure indicators, are positively correlated ($R = 0.995$). Figure S12 presents a pairs plot showing score densities by fibrosis stage, scatterplots for each score pair, and the corresponding correlation coefficients.

As previously observed in the boxplots of structure indicators, there is substantial overlap in the score distributions for F1 and F2, and for F3 and F4, with a clear gap between F2 and F3, where the largest

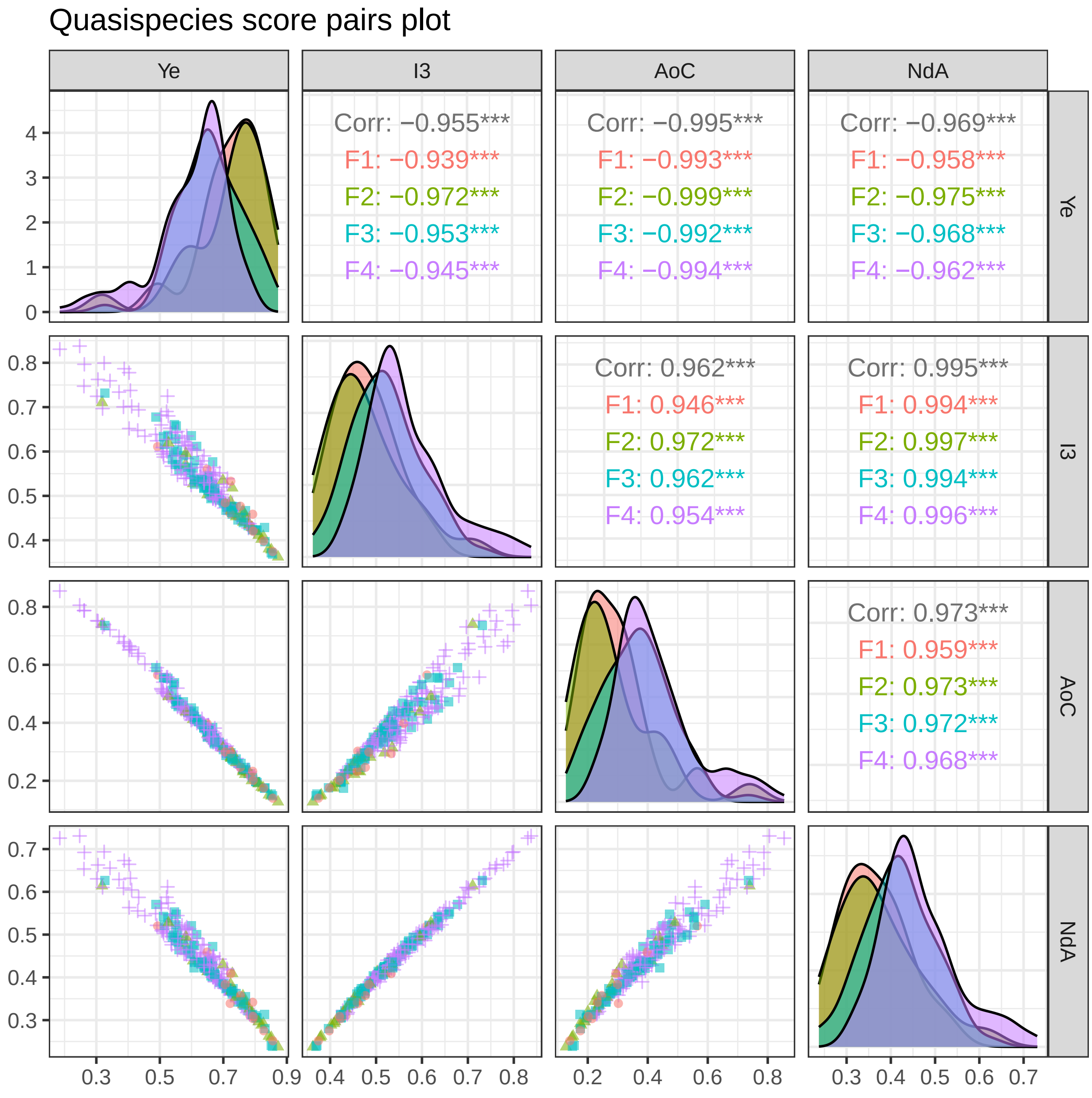

Figure S12: Pairs plot with densities by fibrosis stage, scatterplots of score pairs, and correlation coefficients

Table S7: Maturity scores. Test results, BH adjusted p-values and effect size metrics.

Test	Feature	BH.adj	Signif	AUC	RRB	Gamma
F3 vs F2	Ye	7.834e-03	**	0.684	0.369	-1.080
F3 vs F2	I3	7.834e-03	**	0.673	0.346	0.732
F3 vs F2	AoC	7.834e-03	**	0.701	0.403	1.120
F3 vs F2	NdA	7.834e-03	**	0.669	0.338	0.835
F4 vs F3	Ye	2.564e-03	**	0.618	0.235	-0.191
F4 vs F3	I3	1.211e-03	**	0.642	0.283	0.348
F4 vs F3	AoC	2.564e-03	**	0.617	0.234	0.227
F4 vs F3	NdA	1.211e-03	**	0.635	0.271	0.292

transition differences are observed across all indicators.

Table S7 shows the results of the MW tests on the four maturity scores, and Figure S13 plots the $\gamma_{0.5}$ effect sizes for these indicators in the transitions F2-F3 and F3-F4.

The dominance-related indicators Ye and AoC show the largest effects in the F2–F3 transition, consistent with diversification as the primary structural evolution driver, whereas the evenness-related maturity indicator I3 shows the highest effect size in the F3–F4 transition, consistent with evenness as the main driver. NdA displays an intermediate pattern, with stronger effects in F2–F3. Both maturity scores show considerably larger effect sizes in F2–F3 than in F3–F4. However, the expected time required for each transition is approximately the same ($\approx 8 - 9$ years) (Thein et al. 2008).

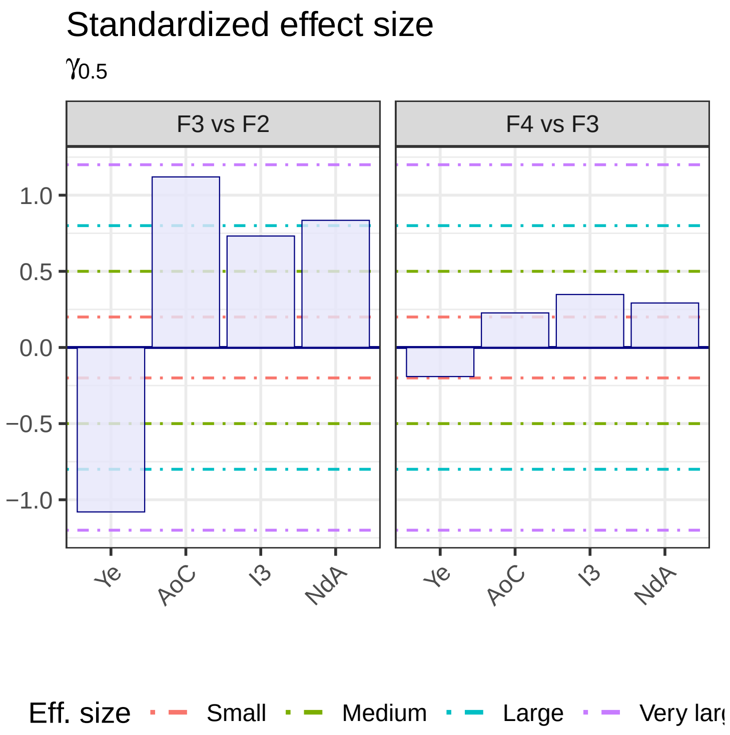

Figure S13: Maturity scores. $\gamma_{0.5}$ effect size, with effect magnitude borders.

1.11 Quasispecies synonymy

In addition to characterizing quasispecies maturity through indicators based on haplotype distribution, haplotype synonymy (Gregori et al. 2022; Gregori, Colomer-Castell, et al. 2024) offers further insights into the maturation process. Synonymy emphasizes the relationship between haplotype diversity and expressed phenotypes. During maturation, multiple haplotypes that express a limited set of functional phenotypes accumulate, resulting in increased haplotype synonymy within the quasispecies. As replication errors and fitness gradients drive the exploration of genetic space primarily through synonymous haplotypes connected to functional phenotypes, more mature quasispecies typically exhibit higher levels of synonymy. This feature can be quantified by calculating the ratio of the master phenotype to the master haplotype frequencies (RMstr) and, more broadly, by comparing the frequencies of the top N phenotypes to the top N haplotypes (RTopN), where $N = \{1, 5, 10, ...\}$. Synonymy provides valuable insights into the quasispecies' evolutionary optimization and its ability to maintain functional stability despite showing very high genetic diversity (Gregori et al. 2022; Gregori, Colomer-Castell, et al. 2024).

Figure S14 shows, on the left, the ratio of master phenotype frequency to master haplotype frequency plotted against master haplotype frequency, and on the right, the same for the top 5 sequences. Higher ratios appear at lower haplotype frequencies in both plots, particularly near zero, where the curve exhibits a hyperbolic singularity.

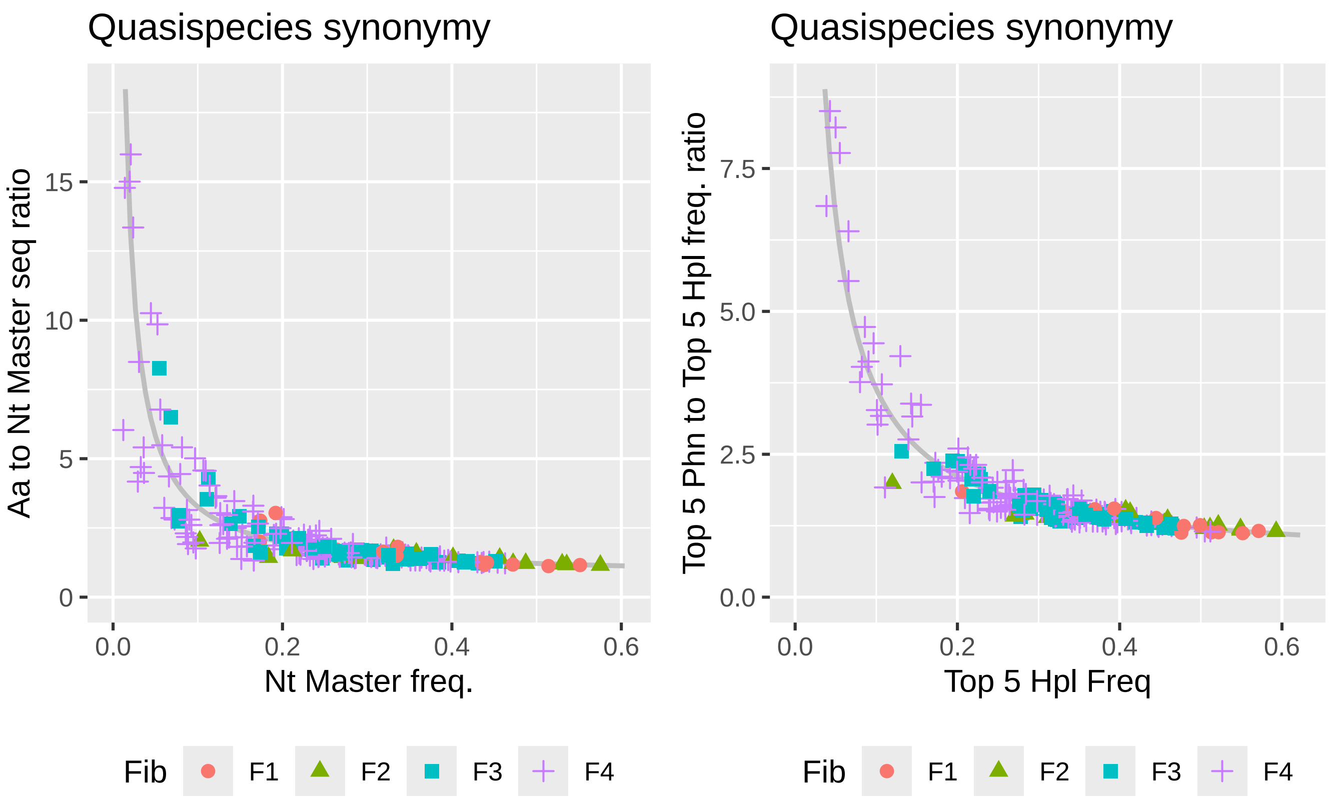

Figure S14: Left: Quasispecies synonymy as the ratio of frequencies top phenotype to top haplotype (RMstr). Right: Quasispecies synonymy as the ratio of frequencies of top 5 phenotypes to top 5 haplotypes (RTop5), with hyperbolic regression curves.

The regression of master phenotype frequency ($AaMstr$) to master haplotype frequency ($NtMstr$) is highly statistically significant with p-value $< 2.2 \times 10^{-16}$, and R^2 0.587, corresponding to a signal-to-noise ratio of 1.42. Table S8 lists regression coefficients, standard errors, and p-vaues. This linear relationship corresponds to the following hyperbola for RMstr, plotted as a grey curve in Figure S14:

Table S8: Linear regression of master phenotype frequency to master haplotype frequency.

	Estimate	Std. Error	t value	Pr(>|t|)
(Intercept)	0.2558	0.0107	23.8504	0
NtMstr	0.7047	0.0386	18.2641	0

Table S9: Linear regression of top 5 phenotypes to top 5 haplotypes aggregated frequencies.

	Estimate	Std. Error	t value	Pr(>|t|)
(Intercept)	0.3040	0.0090	33.7704	0
NtTop5	0.5987	0.0272	21.9874	0

$$RMstr = \frac{AaMstr}{NtMstr} = 0.705 + \frac{0.256}{NtMstr} + \epsilon$$

with $\mathbb{E}(\epsilon) = 0$ and asymptotes $NtMstr = 0$ and $RMstr = 0.705$.

Similarly, the regression of the aggregated frequencies of the top 5 phenotypes (*AaTop5*) to the aggregated frequencies of the top 5 haplotypes (*NtTop5*) is highly statistically significant with p-value $< 2.2 \times 10^{-16}$, and R^2 0.673, corresponding to a 2.06 signal-to-noise ratio. Table S9 lists corresponding coefficients, standard errors and p-vaues. This linear relationship corresponds to the hyperbola:

$$RTop5 = \frac{AaTop5}{NtTop5} = 0.599 + \frac{0.304}{NtTop5} + \epsilon$$

with $\mathbb{E}(\epsilon) = 0$ and asymptotes $NtTop5 = 0$ and $RTop5 = 0.599$.

Note that all studied quasispecies are functional, as they derive from patients with failed DAA treatments. Figure S14 exemplifies the mechanism allowing highly genetically diverse populations to maintain replication capacity: selection prioritizes synonymy as the lower-cost path to preserve functionality.

Table S10 lists the results of the MW tests on synonymy indicators for the F2-to-F3, and F3-to-F4 fibriosis transitions. Figure S15 shows the corresponding $\gamma_{0.5}$ standardized effect sizes. RMstr, Rtop5 and RTop10 are statistically significant in both transitions, with larger effect sizes for F2-to-F3 than for F3-to-F4, as observed above with the quasispecies genetic structure indicators (Figure S7).

1.12 Quasispecies maturity vs. haplotype synonymy

The master haplotype frequency drives both quasispecies maturity and haplotype synonymy. Low master frequencies correspond to elevated evenness and increased maturity. Similarly, low master haplotype frequencies correspond to elevated ratios of master phenotype to master haplotype frequencies, implying increased synonymy in the constituent haplotypes.

Figure S16 shows the relationship between synonymy and maturity, with the former expressed as the ratio of frequencies and the latter by the NdA maturity score.

This relationship follows a double exponential form, which can be linearized using a double logarithmic transformation. The linear regression $\ln(\ln(RTop10)) = a + b\ NdA + \epsilon$, with $\mathbb{E}(\epsilon) = 0$, results in an adjusted R^2 of 0.847, corresponding to a signal-to-noise ratio of 5.52, a p-value $< 2.2 \times 10^{-16}$, and the coefficients listed in Table S11 (See representation in Figure S17).

Table S10: MW test results on synonymy indicators, adjusted p-values and effect size.

Test	Feature	BH.adj	Signif	AUC	RRB	Gamma
F3 vs F2	Rn	1.492e-01		0.588	0.1770	-0.601
F3 vs F2	RMstr	2.453e-03	**	0.744	0.4880	0.702
F3 vs F2	RTop5	1.818e-03	**	0.759	0.5180	0.715
F3 vs F2	RTop10	1.400e-03	**	0.775	0.5510	0.626
F3 vs F2	RTop25	1.400e-03	**	0.791	0.5810	0.800
F4 vs F3	Rn	4.511e-01		0.506	0.0112	0.111
F4 vs F3	RMstr	4.275e-02	*	0.588	0.1760	0.323
F4 vs F3	RTop5	4.275e-02	*	0.591	0.1830	0.288
F4 vs F3	RTop10	4.275e-02	*	0.590	0.1810	0.285
F4 vs F3	RTop25	5.623e-02		0.576	0.1530	0.272

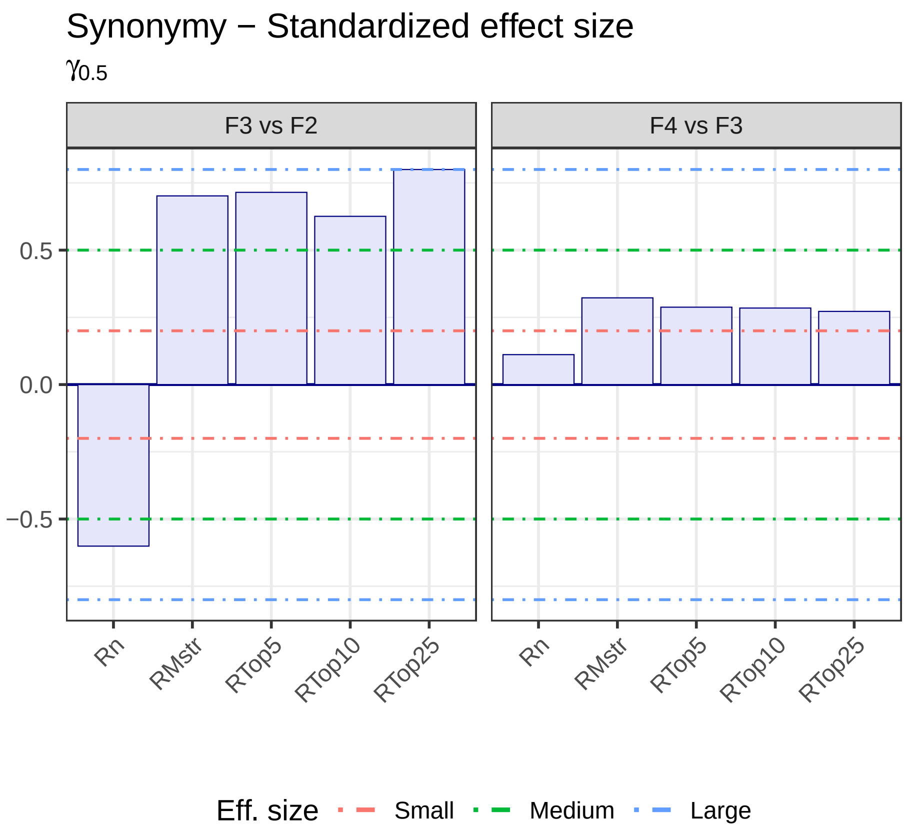

Figure S15: Synonymy indicators. $\gamma_{0.5}$ effect size, with effect magnitude borders.

Table S11: Linear regression of ln(ln(RTop10)) against NdA.

	Estimate	Std. Error	t value	Pr(>|t|)
(Intercept)	-3.2657	0.0679	-48.1235	0
NdA	5.3941	0.1501	35.9475	0

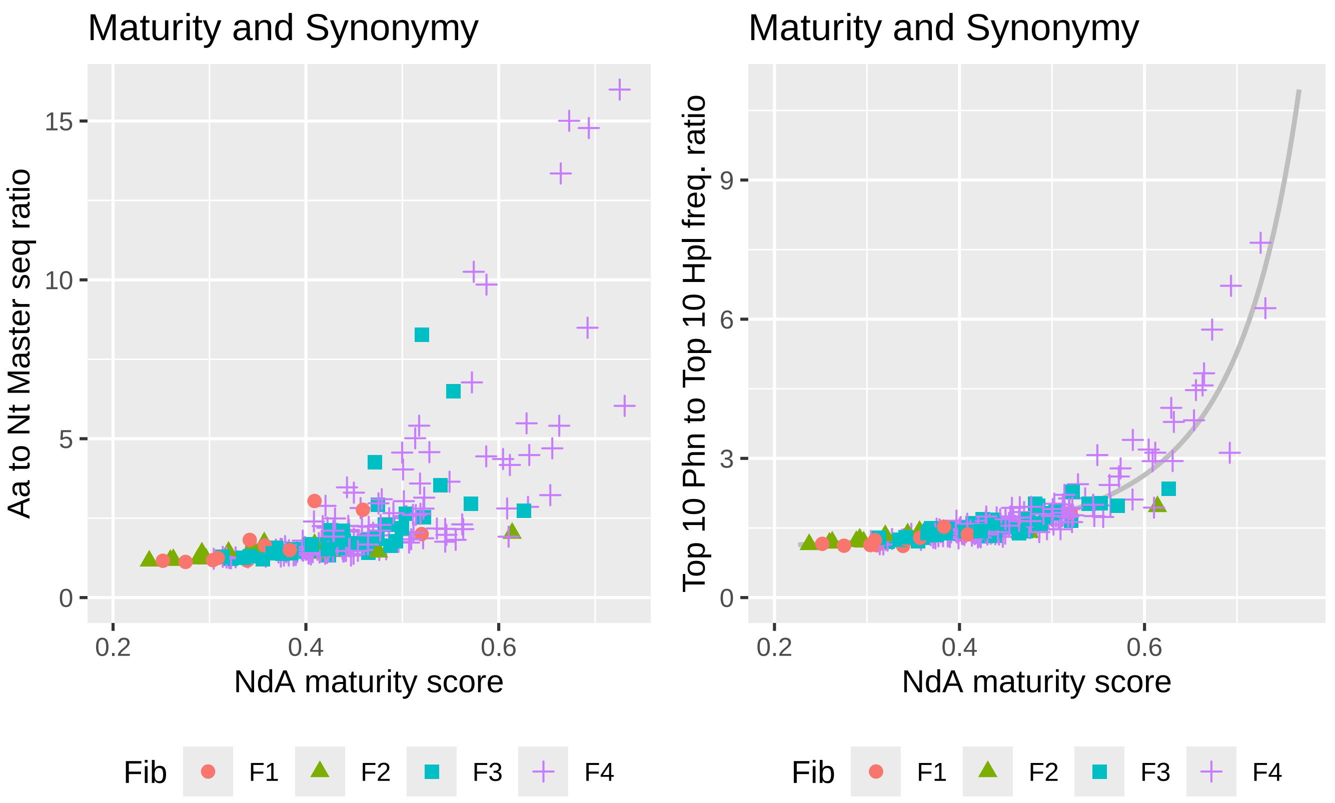

Figure S16: Quasispecies maturity versus haplotype synonymy

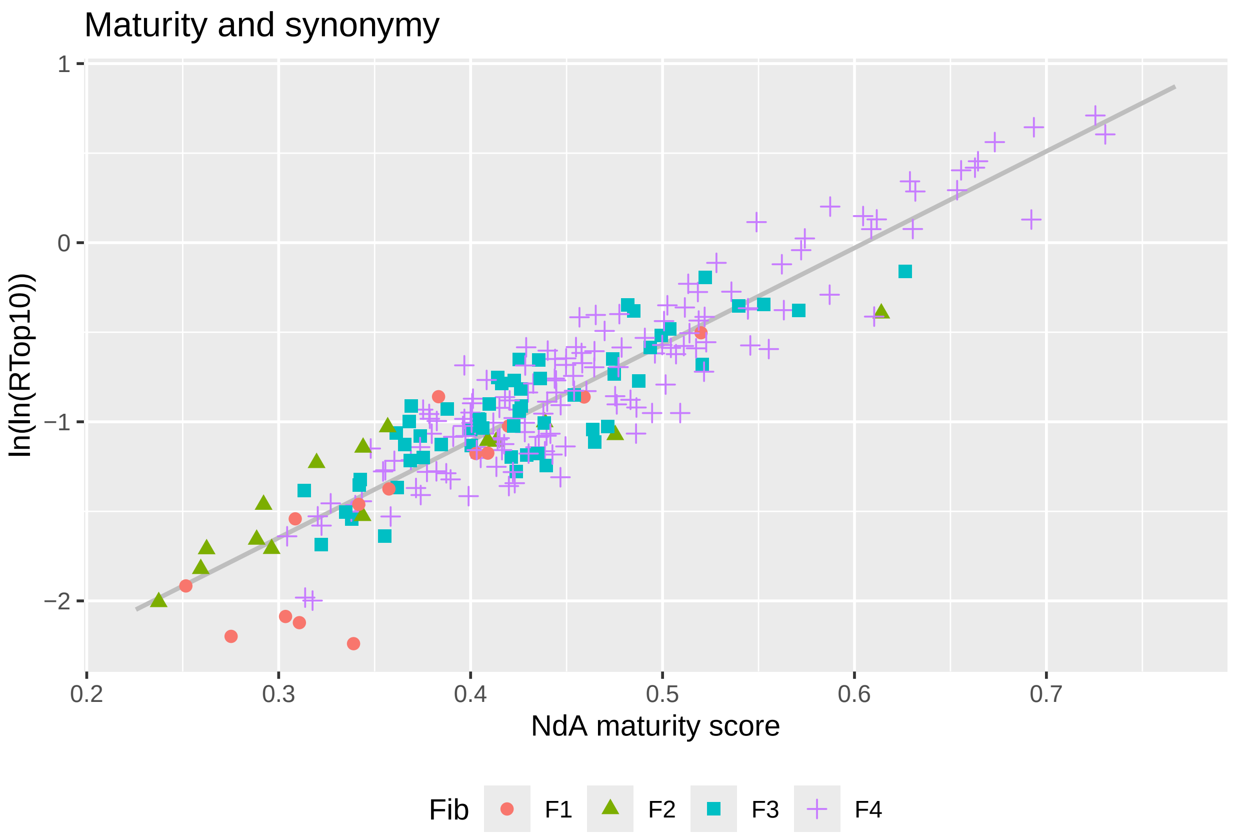

Figure S17: Maturity and synonymy. Double exponential relationship between maturity (NdA score) and synonymy (phenotype-to-haplotype frequency ratio), linearized via double logarithmic transformation.

2 Discussion

As observed with the effect sizes from tests comparing quasispecies structure indicators across consecutive fibrosis stages, quasispecies structural evolution comprises two distinct phases. The first phase is dominated by increasing genetic diversity, declining top haplotype frequencies, and rising rare haplotype fractions. The second phase is characterised primarily by increasing evenness among top haplotypes and, more generally, across the quasispecies, as replication errors from the first phase progressively promote newly fit haplotypes into competition with previously dominant ones.

Test results further indicate that the F2–F3 transition marks a key turning point in HCV quasispecies evolution, involving profound reshaping of genetic structure and broader sequence space exploration. This stage features increased quasispecies maturity and elevated haplotype synonymy, enabling highly diverse populations to maintain functionality under strong selective pressures. Evolution beyond F3 proceeds more gradually by comparison.

Haplotype synonymy was assessed via ratios of cumulative frequencies of top N phenotypes to top N haplotypes (N = {1, 5, 10}). These ratios follow a hyperbolic relationship with top haplotype frequencies, where low top haplotype frequencies yield elevated ratios, indicating greater synonymy as diversification advances. The ratio also exhibits a double-exponential relationship with the NdA maturity score, which linearises under double logarithmic transformation, with an adjusted R^2 of 0.847, such that advanced maturity corresponds to elevated synonymy, preserving functionality despite extreme genetic diversity.

Notably, the association between maturity and synonymy scores exceeds their individual associations with fibrosis stage. Clinical confounders influencing quasispecies maturation exert parallel effects on haplotype synonymy.

References

Akinshin, A. (2020) *Nonparametric Cohen's d-Consistent Effect Size*, Blog posted 2021-06-08. Last accessed 2024-06-03, https://aakinshin.net/posts/nonparametric-effect-size2/.

Benjamini, Yoav, and Yosef Hochberg (1995) 'Controlling the False Discovery Rate: A Practical and Powerful Approach to Multiple Testing', *Journal of the Royal Statistical Society: Series B (Methodological)*, 57/1: 289–300, https://doi.org/10.1111/j.2517-6161.1995.tb02031.x.

Cureton, E. E. (1956) 'Rank-Biserial Correlation', *Psychometrika*, 21/3, https://doi.org/10.1007/BF02289138.

Gregori, J., et al. (2022) 'Quasispecies Fitness Partition to Characterize the Molecular Status of a Viral Population. Negative Effect of Early Ribavirin Discontinuation in a Chronically Infected HEV Patient.', *Int J Mol Sci*, 23/23: 14654:1–17, https://doi.org/10.3390/ijms232314654.

Gregori, J., M. Ibañez-Lligoña et al. (2024) 'Association of Liver Damage and Quasispecies Maturity in Chronic HCV Patients: The Fate of a Quasispecies', *Microorganisms*, 12/11: 2213:1–15, https://doi.org/10.3390/microorganisms12112213.

Gregori, J., S. Colomer-Castell et al. (2024) 'In-Host Flat-Like Quasispecies: Characterization Methods and Clinical Implications', *Microorganisms*, 12/5: 1011:1–14, https://doi.org/10.3390/microorganisms12051011.

Gregori, J., et al. (2025) 'Viral quasispecies inference from single observations—Mutagens as accelerators of

quasispecies maturity', *Microorganisms*, 13/9: 2029, https://doi.org/10.3390/microorganisms13092029.

Hanley, J. A., and B. J. McNeil (1982) 'The Meaning and Use of the Area Under a Receiver Operating Characteristic (ROC) Curve', *Radiology*, 143/1: 29–36, https://doi.org/10.1148/radiology.143.1.7063747.

Kelley, K., and K. J. Preacher (2012) 'On Effect Size', *Psychol Methods*, 17/2, https://doi.org/10.1037/a0028086.

Lötsch, J., and A. Ultsch (2020) 'A Non-Parametric Effect-Size Measure Capturing Changes in Central Tendency and Data Distribution Shape', *PLoS One*, 15/9, https://doi.org/10.1371/journal.pone.0239623.

Mann, H. B., and D. R. Whitney (1947) 'On a Test of Whether One of Two Random Variables Is Stochastically Larger Than the Other', *Ann. Math. Statist.*, 18/1: 50–60, https://doi.org/10.1214/aoms/1177730491.

Nakagawa, S., and I. C. Cuthill (2007) 'Effect Size, Confidence Interval and Statistical Significance: A Practical Guide for Biologists', *Biol Rev Camb Philos Soc*, 82/4: 591–605, https://doi.org/10.1111/j.1469-185X.2007.00027.x.

Thein, H. H., et al. (2008) 'Estimation of Stage-Specific Fibrosis Progression Rates in Chronic Hepatitis c Virus Infection: A Meta-Analysis and Meta-Regression', *Hepatology*, 48/2: 418–31, https://doi.org/10.1002/hep.22375.
