# Supplementary material for: Peaked-to-flat transition in quasispecies structure evolution
Source: Virus Evol. 2026 Apr 14;12(1):veag024. doi: 10.1093/ve/veag024 (PMC13137331; doi:10.1093/ve/veag024)
Supplement: Supplementary_material_veag024 [file supplementary_material_veag024.zip › VEVOLU-2025-229_R2_Supplementary_3_Implementarion_veag024.pdf]

Peaked-to-Flat Transition in Quasispecies Structure Evolution

Supplementary material: Implementation.

Josep Gregori[*], Sergi Colomer-Castell, Carolina Campos, Marta Ibañez-Lligoña, Damir García-Cehic, Alvaro González-Camuesco, Maria Francesca Cortese, David Tabernero, Mar Riveiro-Barciela, Maria Buti, Ariadna Rando-Segura, Roser Ferrer, Tomás Pumarola, Cristina Andrés, Andrés Antón, Francisco Rodríguez-Frías, and Josep Quer[†]

Vall Hebron Hospital - Universitat Autonoma de Barcelona - Barcelona Campus

2026-04-06

Abstract

Previous studies based on clinical data from HCV and HEV infections revealed a deterministic evolution of quasispecies structure, irrespective of haplotype identities, toward a flat-like landscape, characterized by the absence of dominance and high evenness, combined with high haplotype synonymy. Here, two idealized limiting quasispecies states, A and Z, are defined, and it is shown that the A-to-Z evolution describes a parabolic trajectory between these two states. The initial phase is dominated by increasing genetic diversity, whereas the subsequent phase is driven primarily by increasing evenness in the haplotype distribution. This evolutionary progression confers a broad domain within the genetic space, resulting in increased fitness and resilience, accompanied by a diminished response to antiviral therapies and multiple low-cost escape routes. Finally, a normalized quasispecies maturity score is proposed to position a given quasispecies along this evolutionary trajectory. This conceptual framework helps to account for the challenges in treating advanced chronic infections, in which therapeutic failure frequently occurs in the absence of resistance-associated mutations.

Introduction

This supplementary document presents the implementation details of the framework described in the main manuscript. Section 1 provides options of graded difficulty to estimate the maturity state of a quasispecies, together with all required data, including centering and scaling parameters, PCA loadings, and fitted polynomial coefficients. The accompanying figures and tables serve as reference benchmarks to position new quasispecies samples along the A-to-Z maturity continuum and to derive calibration factors that adjust scores obtained through simplified methods. R code snippets are also included to complement the R scripts provided in the appendix of the supplementary document describing the in silico study. Section 2 contains pseudocode outlining the complete workflow, from raw FASTQ input files to FASTA outputs with haplotypes and their corresponding frequencies, to guide implementation of the entire data processing pipeline.

[*]Corresponding authors josep.gregori@gmail.com

[†]and josep.quer@vhir.org

1 Application of the maturity scoring framework

The goal is to assess quasispecies maturity state through proximity to State Z (maximum entropy), as a surrogate of enhanced fitness and treatment resilience. While all proposed indicators prove valuable for quasispecies structure analysis, researchers can implement our framework via four graduated complexity levels, from simple single-metric proxies to advanced curve-based scoring, tailored to their dataset size and analytical objectives:

1. Single-metric (simplest) approach: Use the master haplotype frequency as a proxy; a low master frequency (e.g. <10%) suggests advanced maturity, i.e. a position closer to State Z. No computation is required beyond estimating the frequency of the most abundant haplotype. Alternatively, y_e provides a more comprehensive measure of distributional peakedness, and any of I_3, RLE_3 or RLE_∞ can be used as powerful indicators of distributional evenness.
2. Full metric space: Compute the indicators (as defined in Methods) for each sample. Calculate Euclidean distance from State A in the untransformed indicator space: Maturity $= dA/\|A - Z\|$. Single-sample analysis; no PCA or fitting needed. States A and Z are analytically defined.
3. PCA projection: Apply PCA to your cohort's indicators (or use our loadings as starting point). Project samples onto PC1 / PC2 plane and compute normalized Euclidean distance from A along this reduced space. Requires ≥ 10 samples for stable PCA.
4. Full pipeline: Use our exact centering and scaling parameters, and PCA loadings (Tables A3 and A5) to project your samples onto the HCV-derived PC1/PC2 plane. Use the provided polynomial coefficients to further project onto our reference curve. Compute either:

   -Normalized Euclidean distance from State A in the PC1/PC2 plane, or

   -Normalized arc length along the fitted polynomial curve.

Recommendation: Start with #1 for detection of advanced cases. Use #2 (full space) for broad applicability. Use #4 (our reference PCA and fitted polynomial) when comparing directly to our fibrosis cohort or requiring maximum smoothing. All code, centering and scaling parameters, PCA loadings, and analytic formulas are provided in the next sections.

This graduated approach ensures accessibility while maintaining theoretical rigor across study designs.

This methodology is applied here to quasispecies samples serving as bracketing references with maturity stages below and above the main HCV cohort. These samples are projected onto the PC1/PC2 plane defined by the HCV data, as well as onto both the quadratic and cubic fitted polynomials.

The figures and tables below provide reference benchmarks for positioning new quasispecies samples along the A-to-Z maturity continuum. NdA scores >0.5 indicate a transition zone; scores >0.7 characterize highly diversified yet functional quasispecies (assuming adequate viral loads) with enhanced fitness and resilience to antiviral treatments.

Table A1: Values displayed by different indicators proposed as single metrics to evaluate the maturity stage of a quasispecies. Mean values for each fibrosis stage are given. Table sorted by I3 values.

ID	Virus	Ye	Master	AoC	RLE3	RLEinf	I3
RV.1	CoV-HKU1	0.9444	0.8253	0.0333	0.0332	0.0222	0.2507
RV.5	SARS-CoV-2	0.9443	0.8243	0.0318	0.0339	0.0226	0.2509
RV.4	RSV-B	0.9505	0.8198	0.0313	0.0335	0.0223	0.2511
RV.3	RSV-A	0.9463	0.7964	0.0373	0.0385	0.0257	0.2610
RV.2	CoV-OC43	0.9371	0.7095	0.0558	0.0528	0.0352	0.2914
F1	HCV	0.7298	0.3534	0.2836	0.1779	0.1203	0.4754
F2	HCV	0.7186	0.3434	0.2888	0.1794	0.1202	0.4789
F3	HCV	0.6596	0.2806	0.3637	0.2137	0.1446	0.5217
F4	HCV	0.6022	0.2205	0.4254	0.2605	0.1773	0.5661
HEV.1	HEV	0.5235	0.0882	0.4902	0.3073	0.2052	0.6267
HEV.3	HEV	0.2993	0.0115	0.7713	0.5435	0.3645	0.8075
HEV.5	HEV	0.2657	0.0029	0.8028	0.6520	0.4704	0.8530
HEV.2	HEV	0.2158	0.0041	0.8491	0.6513	0.4453	0.8697
HEV.4	HEV	0.1818	0.0019	0.8750	0.7079	0.5054	0.8984

1.1 Single metric approach

The proposed indicators as single metrics provide compact summaries of quasispecies composition. Master, Y_e, and AoC related to te RACD representation; RLE3, RLEinf and I3 related to the RLE profile. Master and y_e show high values near State A, whereas the other metrics in the table show high values near State Z. The high correlation among them (Figure A1) justify its potential use as single score metrics. The reference values in Table A1 enable grading of new quasispecies samples using either a single metric or any combination of these indicators.

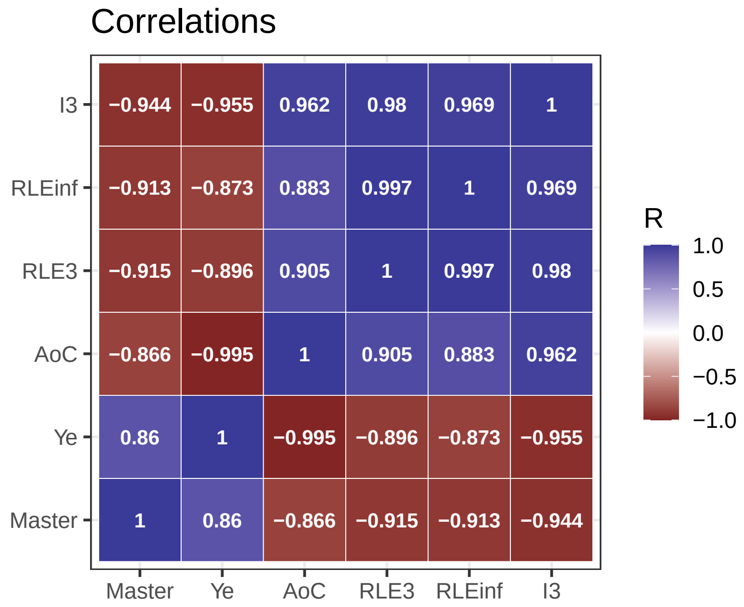

Figure A1: Correlations among the proposed scores to assess the maturity stage of a quasispecies in the full HCV dataset.

Table A2: Indicator values of bracketing samples, and mean values per fibrosis stage of HCV patients.

ID	Virus	Master	Rare1	Singl	RLE1	RLE2	RLEinf
RV.1	CoV-HKU1	0.82531	0.17469	0.01630	0.19126	0.04430	0.02216
RV.5	SARS-CoV-2	0.82432	0.17568	0.01490	0.19053	0.04524	0.02263
RV.4	RSV-B	0.81978	0.18022	0.01728	0.19188	0.04463	0.02232
RV.3	RSV-A	0.79638	0.20362	0.02093	0.21248	0.05130	0.02566
RV.2	CoV-OC43	0.70946	0.29054	0.03971	0.27763	0.07032	0.03518
F1	HCV	0.35335	0.57844	0.22265	0.60564	0.23158	0.12029
F2	HCV	0.34335	0.61463	0.23748	0.61122	0.23572	0.12022
F3	HCV	0.28059	0.67153	0.30423	0.68032	0.27788	0.14460
F4	HCV	0.22054	0.72246	0.36186	0.73271	0.33528	0.17733
HEV.1	HEV	0.08817	0.88544	0.40674	0.82450	0.40198	0.20519
HEV.3	HEV	0.01149	0.98851	0.70070	0.95671	0.69400	0.36451
HEV.2	HEV	0.00415	1.00000	0.78417	0.97825	0.80520	0.44533
HEV.5	HEV	0.00293	1.00000	0.73428	0.96501	0.76815	0.47042
HEV.4	HEV	0.00190	1.00000	0.81824	0.98402	0.85715	0.50541

Table A3: Centering and scaling parameters to be applied previous to the Principal Components decompostion.

	mu	sigma
Master	0.2559575	0.1341468
Rare1	0.6905525	0.1222003
Singl	0.3297462	0.1405670
RLE1	0.7000528	0.1183749
RLE2	0.3074577	0.1239211
RLEinf	0.1632709	0.0853376

1.2 Centering and scaling a new matrix of indicator values as per the HCV cohort

The centering and scaling parameters from the HCV cohort are given in Table A3. The matrix of new quasspecies samples is listed in Table A2, and the resulting centered and scaled matrix is given in Table A4.

With these parameters (μ and σ) a new matrix of indicator values $\mathbf{X}$ may be centered and scaled to $\mathbf{X}_{cs}$ as :

$$\mathbf{X}_{sc} = (\mathbf{X} - \mathbf{1}\ \mu^{\top}) \oslash (\mathbf{1}\ \sigma^{\top})$$

where:

- $\mathbf{1}_n$ = n×1 vector of ones (broadcasts row-wise)
- $\boldsymbol{\mu}$, $\boldsymbol{\sigma}$ = p×1 column mean/std vectors (broadcasts column-wise)
- $\oslash$ = element-wise division

In R code this operation is easily implemented as:

Table A4: Centered and scaled indicator values of bracketing samples. Includng mean values per fibrosis stage of HCV patients and limiting states A and Z.

	Master	Rare1	Singl	RLE1	RLE2	RLEinf
A	5.54648	-5.65099	-2.34583	-5.91386	-2.48108	-1.91323
RV.1	4.24424	-4.22144	-2.22990	-4.29816	-2.12358	-1.65360
RV.5	4.23687	-4.21335	-2.23982	-4.30432	-2.11600	-1.64805
RV.4	4.20301	-4.17618	-2.22292	-4.29293	-2.12093	-1.65168
RV.3	4.02857	-3.98469	-2.19694	-4.11889	-2.06714	-1.61253
RV.2	3.38064	-3.27341	-2.06336	-3.56852	-1.91363	-1.50103
F1	0.72602	-0.91742	-0.76185	-0.79755	-0.61227	-0.50363
F2	0.65147	-0.62126	-0.65639	-0.75042	-0.57894	-0.50446
F3	0.18363	-0.15563	-0.18150	-0.16670	-0.23866	-0.21880
F4	-0.26406	0.26108	0.22843	0.27585	0.22451	0.16471
HEV.1	-1.25074	1.59478	0.54772	1.05126	0.76273	0.49125
HEV.3	-1.82241	2.43829	2.63899	2.16815	3.11928	2.35818
HEV.2	-1.87713	2.53230	3.23278	2.35016	4.01665	3.30520
HEV.5	-1.88618	2.53230	2.87785	2.23826	3.71760	3.59919
HEV.4	-1.89387	2.53230	3.47518	2.39887	4.43584	4.00926
Z	-1.90804	2.53230	4.76822	2.53388	5.58857	9.80493

Table A5: PC1 and PC2 loadings.

	PC1	PC2
Master	-0.4038104	-0.2524660
Rare1	0.4106220	0.4128276
Singl	0.4115512	0.0116318
RLE1	0.4194132	0.3505905
RLE2	0.4180471	-0.3466455
RLEinf	0.3850735	-0.7229261

```
X_sc <- sweep( sweep(X, 2, mu, "-"), 2, sd, "/")
```

1.3 Projecting the new quasispecies samples on the HCV cohort PC1/PC2 plane

The PCA loadings for the first two principal components are provided in Table A5. The inherent sign indeterminacy of PCA was resolved by orienting State A at negative PC1 values (left side) and positioning the parabola apex at positive PC2 values (upper side). The resulting PC1 coefficients reveal a clear gradient: high master haplotype frequency aligns with proximity to State A (peaked distribution), while high values of all other indicators correspond to proximity to State Z (flat distribution). This systematic opposition confirms PC1 as a meaningful maturity axis distinguishing quasispecies structure extremes.

Notably, the absolute values of all PC1 loadings cluster tightly around the theoretical equivalence value of $\sqrt{1/6} = 0.4082$, reflecting the balanced contribution of the six indicators to the primary axis of variation. This near-uniform loading (≈ 40.8 per indicator) confirms PC1 as a robust, equidistributed maturity coordinate spanning the peaked-to-flat spectrum.

The rotations implied by these loadings, $\mathbf{V_{12}}$, are now applied to the centered and scaled matrix,

Table A6: Coordinates on the PC1/PC2 plane of bracketing samples, including mean values per HCV fibrosis stage and limiting states A and Z.

ID	x	y
A	-9.77987	-3.59063
RV.1	-7.69222	-2.41552
RV.5	-7.68729	-2.41924
RV.4	-7.65007	-2.38682
RV.3	-7.37976	-2.24936
RV.2	-6.43313	-1.73145
F1	-1.76783	-0.27418
F2	-1.53933	-0.12630
F3	-0.46669	0.06974
F4	0.58082	0.07692
HEV.1	2.33426	0.72954
HEV.3	5.94464	-0.52856
HEV.2	7.06586	-1.40091
HEV.5	6.86470	-1.55085
HEV.4	7.63916	-2.03108
Z	10.94731	-6.55456

$\mathbf{X}_{sc}$, effectively projecting the new quasispecies samples on the PC1/PC2 plane of the HCV cohort.

$$\mathbf{X}_{pca} = \mathbf{X}_{sc}\mathbf{V}_{12}$$

with PC1 coordinates in the first column, and PC2 coordinates in the second. This operation is implemented in R code as a matrix multiplication:

```
X_pca <- X_sc %*% V_12
```

1.4 Fitted polynomials to the HCV cohort data

The quadratic polynomial curve fitted to the HCV cohort data show the following summary of results:

```
## Summary of a square polynomial fit to HCV cohort data.
```

```
##
## Call:
## lm(formula = y ~ poly(x, 2, raw = TRUE), data = pca.df)
##
## Residuals:
##      Min       1Q   Median       3Q      Max
## -1.82532 -0.12291  0.02029  0.14503  1.23195
##
## Coefficients:
##                          Estimate Std. Error t value Pr(>|t|)
## (Intercept)              0.252030   0.020888  12.066  < 2e-16 ***
## poly(x, 2, raw = TRUE)1  0.059358   0.008394   7.071  1.4e-11 ***
## poly(x, 2, raw = TRUE)2 -0.046987   0.001699 -27.660  < 2e-16 ***
```

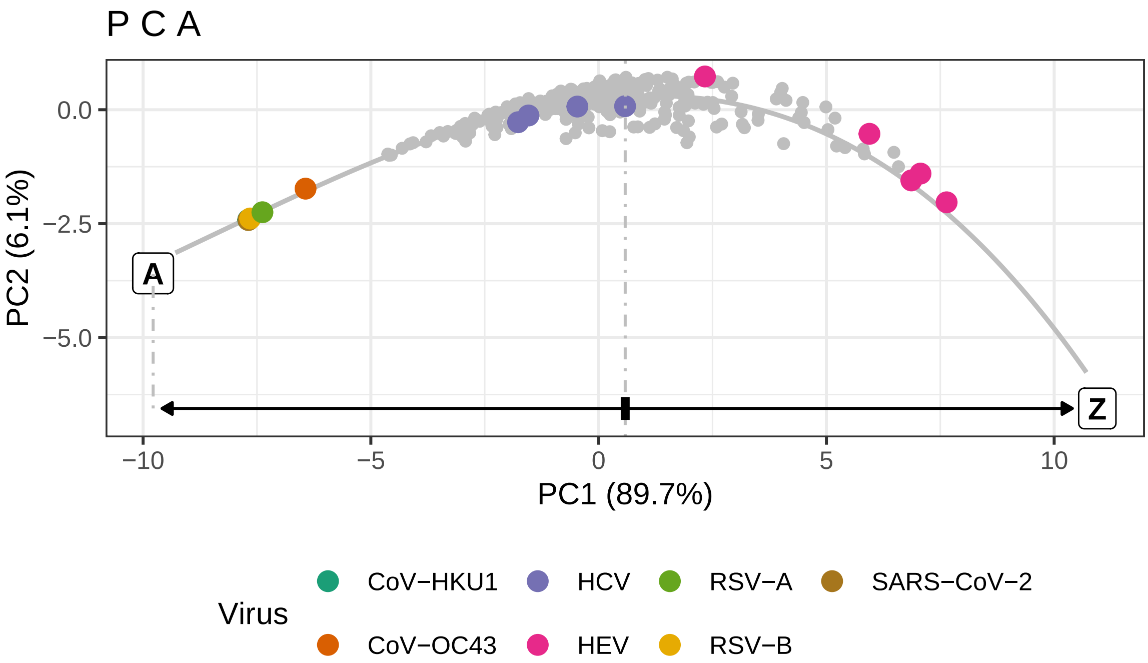

Figure A2: Projection of quasispecies on the PC1/PC2 plane explaining 98.6% of the total variance of the dataset. Bracketing quasispecies shown over the grayed HCV data, including means per fibrosis stage and limiting states A and Z, with fitted cubic polynomial on the HCV dataset. Dash-dot line on curve apex.

```
## ---
## Signif. codes:  0 '***' 0.001 '**' 0.01 '*' 0.05 '.' 0.1 ' ' 1
##
## Residual standard error: 0.306 on 262 degrees of freedom
## Multiple R-squared:  0.7449, Adjusted R-squared:  0.743
## F-statistic: 382.5 on 2 and 262 DF,  p-value: < 2.2e-16
```

The arc length along the quadratic polynomial curve, $y = Ax^2 + Bx + C$, between x_1 and x_2, is calculated analytically as:

$$L(x_1, x_2) = \int_{x_1}^{x_2} \sqrt{1 + f'(x)^2}\ dx = \int_{x_1}^{x_2} \sqrt{1 + (2A\,x + B)^2}\ dx =$$

$$= \frac{1}{4A}\left[(2Ax + B)\sqrt{1 + (2Ax + B)^2} + \ln\left(\left|(2Ax + B) + \sqrt{1 + (2Ax + B)^2}\right|\right)\right]_{x_1}^{x_2}$$

which may be implemented in R code with the following function, using the fitted coefficients given above, and where x_1 is the PC1 coordinate of State A, and x_2 the PC1 coordinate of the quasispecies being evaluated:

```r
parab.arc.lebgth <- function(x1,x2,A,B)
{ dv2 <- (2*A*x2+B)
  ix2 <- 1/(4*A)*(dv2*sqrt(1+dv2^2)+log(abs(dv2+sqrt(1+dv2^2))))
  dv1 <- (2*A*x1+B)
  ix11 <- 1/(4*A)*(dv1*sqrt(1+dv1^2)+log(abs(dv1+sqrt(1+dv1^2))))
  ix2-ix1
}
```

The cubic polynomial curve fitted to the HCV cohort data show the following summary of results:

```
## Summary of a cubic polynomial fit to HCV cohort data.

##
## Call:
## lm(formula = y ~ poly(x, 3, raw = TRUE), data = pca.df)
##
## Residuals:
##      Min       1Q   Median       3Q      Max
## -1.00034 -0.12215  0.05098  0.15925  0.61509
##
## Coefficients:
##                          Estimate Std. Error t value Pr(>|t|)
## (Intercept)             0.2468252  0.0186594  13.228  < 2e-16 ***
## poly(x, 3, raw = TRUE)1  0.1070362  0.0094739  11.298  < 2e-16 ***
## poly(x, 3, raw = TRUE)2 -0.0438247  0.0015646 -28.010  < 2e-16 ***
## poly(x, 3, raw = TRUE)3 -0.0017350  0.0002109  -8.227 9.16e-15 ***
## ---
## Signif. codes:  0 '***' 0.001 '**' 0.01 '*' 0.05 '.' 0.1 ' ' 1
##
## Residual standard error: 0.2732 on 261 degrees of freedom
## Multiple R-squared:  0.7974, Adjusted R-squared:  0.7951
## F-statistic: 342.5 on 3 and 261 DF,  p-value: < 2.2e-16
```

The arc length along the cubic polynomial curve, $y = Ax^3 + Bx^2 + Cx + D$, between x_1 and x_2, is obtained by numeric integration of the equation:

$$L(x_1, x_2) = \int_{x_1}^{x_2} \sqrt{1 + f'(x)^2}\ dx = \int_{x_1}^{x_2} \sqrt{1 + (3A\,x^2 + 2B\,x + C)^2}\ dx$$

which may be implemented with R code as:

```
cubic_arc_length <- function(x1, x2, A, B, C) {
  integrand <- function(x) sqrt(1 + (3*A*x^2 + 2*B*x + C)^2)
  integrate(integrand, x1, x2)$value
}
```

using the coefficients given above, obtained from the fitted cubic polynomial on the HCV cohort data, and where x_1 is the PC1 coordinate of State A, and x_2 the PC1 coordinate of the quasispecies being evaluated.

1.5 Projecting the new quasispecies samples on the HCV fitted polynomial

With the PC1 coordinates of the new samples, the corresponding PC2 coordinates on the fitted curve on the HCV cohort data may be directly derived using the fitted coefficients. See Tables A7 and A8 with the resulting coordinates on the cubic and quadratic polynomials respectively.

Table A7: Quasispecies coordinates on the fitted cubic polynomal. x original PC1 coordinates, y original PC2 coordinates, PC1 and PC2 coordinates of the quasispecies on the fitted cubic polynomial. Average values per HCV fibrosis stage included.

ID	x	y	PC1	PC2
A	-9.7799	-3.5906	-9.7799	-3.3687
RV.1	-7.6922	-2.4155	-7.6922	-2.3799
RV.5	-7.6873	-2.4192	-7.6873	-2.3776
RV.4	-7.6501	-2.3868	-7.6501	-2.3600
RV.3	-7.3798	-2.2494	-7.3798	-2.2325
RV.2	-6.4331	-1.7315	-6.4331	-1.7935
F1	-1.7678	-0.2742	-1.7678	-0.0698
F2	-1.5393	-0.1263	-1.5393	-0.0155
F3	-0.4667	0.0697	-0.4667	0.1875
F4	0.5808	0.0769	0.5808	0.2939
HEV.1	2.3343	0.7295	2.3343	0.2358
HEV.3	5.9446	-0.5286	5.9446	-1.0301
HEV.2	7.0659	-1.4009	7.0659	-1.7970
HEV.5	6.8647	-1.5509	6.8647	-1.6449
HEV.4	7.6392	-2.0311	7.6392	-2.2665
Z	10.9473	-6.5546	10.9473	-6.1098

Table A8: Quasispecies coordinates on the fitted quadratic polynomal. x original PC1 coordinates, y original PC2 coordinates, PC1 and PC2 coordinates of the quasispecies on the fitted quadratic polynomial. Average values per HCV fibrosis stage included.

ID	x	y	PC1	PC2
A	-9.7799	-3.5906	-9.7799	-4.8226
RV.1	-7.6922	-2.4155	-7.6922	-2.9848
RV.5	-7.6873	-2.4192	-7.6873	-2.9809
RV.4	-7.6501	-2.3868	-7.6501	-2.9519
RV.3	-7.3798	-2.2494	-7.3798	-2.7450
RV.2	-6.4331	-1.7315	-6.4331	-2.0744
F1	-1.7678	-0.2742	-1.7678	0.0003
F2	-1.5393	-0.1263	-1.5393	0.0493
F3	-0.4667	0.0697	-0.4667	0.2141
F4	0.5808	0.0769	0.5808	0.2707
HEV.1	2.3343	0.7295	2.3343	0.1346
HEV.3	5.9446	-0.5286	5.9446	-1.0556
HEV.2	7.0659	-1.4009	7.0659	-1.6744
HEV.5	6.8647	-1.5509	6.8647	-1.5547
HEV.4	7.6392	-2.0311	7.6392	-2.0365
Z	10.9473	-6.5546	10.9473	-4.7292

Table A9: Normalized distance from state A (NdA). Approaches: dN.Full: Euclidean distance in full indicator space. dN.PCA: Euclidean distance on reduced PC1/PC2 plane. dN.Parab: Arc length along fitted quadratic polynomial curve. dN.Cubic: Arc length along fitted cubic polynomial curve. Average values per HCV fibrosis stage included.

ID	Virus	dN.Full	dN.PCA	dN.Parab	dN.Cubic
RV.1	CoV-HKU1	0.12931	0.11558	0.11767	0.09683
RV.5	SARS-CoV-2	0.12962	0.11570	0.11794	0.09706
RV.4	RSV-B	0.13201	0.11803	0.11994	0.09878
RV.3	RSV-A	0.14821	0.13265	0.13433	0.11131
RV.2	CoV-OC43	0.20561	0.18471	0.18339	0.15505
F1	HCV	0.45410	0.41835	0.40051	0.36384
F2	HCV	0.46708	0.43128	0.41039	0.37368
F3	HCV	0.51994	0.48278	0.45630	0.41945
F4	HCV	0.56879	0.53025	0.50068	0.46361
HEV.1	HEV	0.66638	0.62051	0.57513	0.53725
HEV.3	HEV	0.81395	0.77289	0.73651	0.69879
HEV.2	HEV	0.85735	0.81957	0.79068	0.75577
HEV.5	HEV	0.84418	0.80904	0.78079	0.74519
HEV.4	HEV	0.87789	0.84376	0.81936	0.78683

1.6 Alternative metrics for the normalized distance from state A

Table A9 lists the NdA values obtained from different distance approximations, plotted in Figure A3. The approaches considered are:

- dN.Full: Euclidean distance from State A computed directly from the full set of indicator values.
- dN.PCA: Euclidean distance from State A on the PC1/PC2 plane.
- dN.Parab: Arc length along the fitted quadratic polynomial from State A.
- dN.Cubic: Arc length along the fitted cubic polynomial from State A.

Differences between approaches are small for low NdA scores (<0.25), but substantial for advanced maturity (>0.5). These observed discrepancies provide calibration factors to correct scores obtained via simpler methods, enhancing comparability across studies using different approximations.

1.7 R code to compute quasispecies indicators values

Function `diversity()` takes a single parameter `nr` as the vector of haplotype read counts, and returns a named vector with the following diversity indices and quasispecies indicators:

- `q0, q1, q2, q3`, and `qinf` as the Hill numbers of orders q=0,1,2,3, and infinity.
- `Master`: frequency of the most abundant haplotype
- `Top25`: Cumulated frequency of the 25 top ranked haplotypes.
- `Rare2`: Cumulated frequency of all haplotypes with abundances < 0.1%
- `R5` and `R10`: The ratio of the frequency of the 5 or 10 ranked haplotype, to the mean of the top 5 or 10 top ranked haplotypes.

```
### Names of the elements in the returned diversity vector
div.nms <- c('q0','q1','q2','q3','qinf','Master','Top25','Rare2','R5','R10')
```

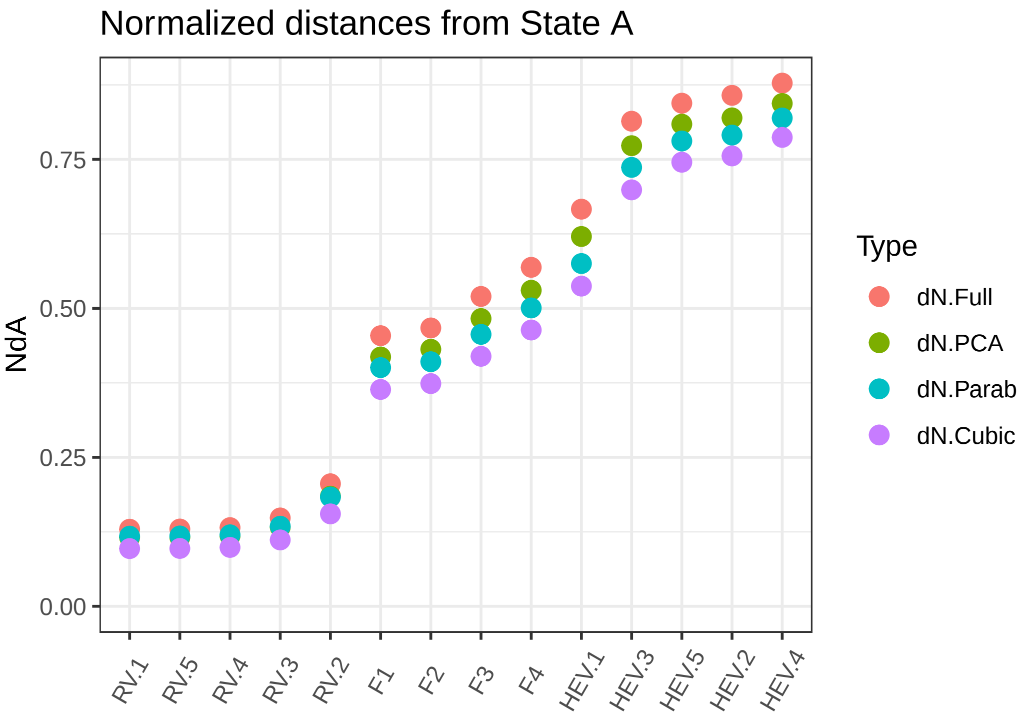

Figure A3: The different approximations to the NdA score. dN.Full: Euclidean distance in full indicator space. dN.PCA: Euclidean distance on reduced PC1/PC2 plane. dN.Parab: Arc length along fitted quadratic polynomial curve. dN.Cubic: Arc length along fitted cubic polynomial curve. Average values per HCV fibrosis stage included.

```
###  Function computing selected diversity indices
diversity <- function(nr)
{
   p <- nr/sum(nr)
   q_0 <- length(p)              # Hill number for q=0
   q_1 <- exp(-sum(p*log(p)))    # Hill number for q=1
   q_2 <- 1/sum(p^2)             # Hill number for q=2
   q_3 <- 1/sqrt(sum(p^3))       # Hill number for q=3
   q_inf <- 1/max(p)             # Hill number for q=Infinity

   p <- sort(p,decreasing=TRUE)  # Frequencies in decreasing order
   Master <- p[1]                # Master frequency
   Top25 <- sum(p[1:25])         # Top 25 haplotypes, reads fraction
   Rare2 <- sum(p[p<0.001])      # Fraction of reads for all Hpl <0.1%
   R5 <- p[5]/mean(p[1:5])       # Evenness in top 5 haplotypes
   R10 <- p[10]/mean(p[1:10])    # Evenness in top 10 haplotypes

   vdiv <- c(q_0,q_1,q_2,q_3,q_inf,Master,Top25,Rare2,R5,R10)
   names(vdiv) <-
       c('q0','q1','q2','q3','qinf','Master','Top25','Rare2','R5','R10')
   return(vdiv)
}
```

Given the matrix of diversity values of the quasispecies in the dataset, `div.vals`, RLE and I3

values are further computed as:

```
library(tidyverse)

mat.vals <- div.vals %>%
  mutate(RLE1=log(q1)/log(q0),
         RLE2=log(q2)/log(q0),
         RLE3=log(q3)/log(q0),
         RLEinf=log(qinf)/log(q0),
         I3=1/6*(1+2*log(q1)/log(q0)+2*log(q2)/log(q0)+
                   log(q3)/log(q0)))
```

where library `tidyverse` (Wickham et al. 2019) is used.

Additional R code and functions are provided in the supplementary document containing the *in silico* study, where the complete code used to analyse the simulated quasispecies structures is available in the appendix. Given the vector of haplotype frequencies for a quasispecies, these functions may be applied directly.

2 Pseudocode to process raw illumina 2x300 amplicon paired-end FASTQ files

The following subsections present pseudocode in Programming Language Style to process raw Illumina 2x300 paired-end FASTQ files from sequenced amplicons to fasta files containing amplicon haplotypes and frequencies. These FASTA files form the basis for downstream quasispecies diversity and structure analysis.

The aim of the sequencing data treatment is to discard error-bearing reads while preserving full-length read integrity, so that haplotypes that completely cover the amplicon with their respective frequencies are incorporated (Gregori et al. 2022, 2024).

Briefly, the process is composed of the following steps :

1 - Obtain fastq files with Illumina (R) 2 $\times$ 300 bp paired-end reads.

2 - Recover full amplicon reads with FLASH (Magoc and Salzberg 2011) (minimum 20 bp overlap, maximum of 10% mismatches). The paired-end reads, when overlapped, result in reads covering the complete amplicon.

3 - Remove full reads with 5% or more bases below a Phred score of Q30, representing a 99.9% accuracy. Remove reads with indeterminations.

4 - Demultiplex and trim primers (max three mismatches accepted), identify strand.

5 - Collapse reads (molecules) to haplotypes (amplicon-genomes) and their frequencies as read counts and percentages.

From the provided pseudocode, a bioinformatician can readily implement this data-processing framework in any interpreted language (e.g. R, Python, Perl), any compiled language (e.g. C, C++, C#, Fortran), or a combination of both. A particularly attractive option is to combine R with C++, for which mature tools exist to allow seamless interaction between functions and subroutines in both languages. Interpreted languages generally offer faster development and easier prototyping, whereas compiled languages typically provide substantially faster execution times.

Following the pseudocode subsections, recommendations for R libraries and functions for each step are provided to assist R users with implementation.

2.1 Overlap of paired-ends to amplicon reads with FLASH

FLASH (Magoc and Salzberg 2011) is a computational tool to extend the length of short reads by overlapping paired-end reads from fragment or amplicon libraries. It is a fast computational solution that enables short 300 bp fragments to accurately reconstruct amplicons longer than 450 bp. It is implemented in C and freely available as open-source code at http://www.cbcb.umd.edu/software/flash.

It is executed as a system command to overlap two paired-end FASTQ files `matesR1.fastq` and `matesR2.fastq` using the following syntax:

```
flash <matesR1.fastq> <matesR2.fastq> [-m minOverlap] [-M maxOverlap] [-x mismatchRatio]
[-p phredOffset] [-o prefixOfOutputFiles] [-d pathToDirectoryForOutputFiles]
[-f averageFragment Length] [-s standardDeviationOfFragments] [-r averageReadLength]
[-h displayHelp]
```

The input files <...> are mandatory; all options [...] are optional with default values described in the manual at https://github.com/genome-vendor/FLASH/blob/master/MANUAL

The following pseudocode illustrates its usage::

```
INPUT:
    R1_fastq            # matesR1.fastq (forward reads, 2x300)
    R2_fastq            # matesR2.fastq (reverse reads, 2x300)
    min_overlap  = 20          # Min. overlap required. Default: 10bp.
    max_overlap = 300          # Max. overlap. Default: 70bp.
    max_mismatch_ratio = 0.10  # Max. differences in overlap. Default: 0.25
    phredOffset = 33           # Base calling quality ASCII offset. Default: 33.
    out_prefix   = "ID.xxxx"   # Prefix of output files. Default: 'out'.

STEP 1: Build FLASH command
    cmd = "flash " +
          R1_fastq + " " + R2_fastq +
          " -m " + STRING(min_overlap) +
          " -M " + STRING(max_overlap) +
          " -x " + STRING(max_mismatch_ratio) +
          " -p " + STRING(phred_offset) +
          " -o " + out_prefix

STEP 2: Run FLASH
    EXECUTE_SYSTEM_COMMAND(cmd)

FLASH will:
- For each read pair, consider all ungapped overlaps of length >=
   min_overlap.
- For each possible overlap, compute mismatch_ratio = (number_of_mismatches)
   / (overlap_length).
- Choose the best overlap whose mismatch_ratio <= max_mismatch_ratio
  (here 0.10); if none, do not merge.
- Generate merged amplicons and report unmerged reads separately.

STEP 3: Collect output files (FLASH naming)
    merged_amplicons_fastq   = out_prefix + ".extendedFrags.fastq"   # merged reads
    unmerged_R1_fastq        = out_prefix + ".notCombined_1.fastq"   # unmerged R1
    unmerged_R2_fastq        = out_prefix + ".notCombined_2.fastq"   # unmerged R2
    # (additional log/stats: out_prefix + ".hist", ".histogram", etc.)
```

Use `merged_amplicons_fastq` as the amplicon read set. Optionally, keep `unmerged_R1_fastq` and `unmerged_R2_fastq` for separate analysis.

2.2 Quality filter on fastq files

In Illumina MiSeq sequencing in 2×300 mode, quality typically decreases past position 220-250 bp for R1 reads and slightly earlier (200-230 bp) for R2 reads. This results in the lowest qualities at the 3' ends of both R1 and R2 reads, which impairs overlap performance particularly for amplicons >450 bp. FLASH merging thus serves as an effective intrinsic quality filter by only retaining read pairs that overlap with sufficient quality. This effectively serves as a full read quality filter: reads are either successfully merged or discarded. A high-quality MiSeq run for amplicons around 400–450 bp typically yields ~90% FLASH merged reads.

Beyond this implicit quality filter, an additional step discards reads containing a number, or fraction, of bases with Phred scores below a quality threshold. For high quality MiSeq runs,

setting the threshold to Q30 and the maximum fraction to 5% typically results in yields above 80%. This is implemented with the following pseudocode:

```
INPUT:
    in_fastq      # input merged_amplicons_fastq file
    out_fastq     # output FASTQ .q30.fastq file
    q_threshold   = 30     # Q30: 99.9% acuracy
    max_frac_bad  = 0.05   # Max. 5% below Q30

OPEN in_fastq for reading
OPEN out_fastq for writing

WHILE not end of in_fastq:
    READ line1   # '@' header
    READ line2   # sequence
    READ line3   # '+'
    READ line4   # quality string

    IF any of line1..line4 is missing:
        BREAK or raise error  # malformed FASTQ

    read_len = length(line2)

    bad_bases = 0
    has_Ns = FALSE

    FOR i from 0 to read_len-1:
        nt_char = line2[i]
        IF nt_char == 'N':
            has_Ns = TRUE
            BREAK

        q_char = line4[i]
        phred  = ASCII_CODE(q_char) - 33   # PHRED+33 decoding

        IF phred < q_threshold:
            bad_bases = bad_bases + 1

    frac_bad = bad_bases / read_len

    IF frac_bad < max_frac_bad AND NOT has_Ns:
        WRITE line1 to out_fastq
        WRITE line2 to out_fastq
        WRITE line3 to out_fastq
        WRITE line4 to out_fastq
    ELSE:
        # discard read (do not write)

CLOSE in_fastq
CLOSE out_fastq
```

2.3 Identify and trim amplicon specific primers

In NGS, a pool may consist of different amplicons from the same biosample, each identifiable by a specific pair of primers. Each pool in a run results in a pair of FASTQ files, which are subsequently merged with FLASH to obtain a single FASTQ file per pool. Demultiplexing a pool then consists of identifying reads belonging to each amplicon and strand, and trimming the primers to yield the bare amplicon sequence.

The following pseudocode describes this demultiplexing process, which generates a pair of FASTA files for each primer pair in the pool from a single FASTQ file; one containing forward-oriented sequences, the other containing reverse-oriented sequences.

Up to three mismatches are permitted in primer alignment, and primers must be located within a narrow positional window close to the read ends.

```
INPUT:
    q30_fastq             # quality-filtered FASTQ file (merged amplicons)
    primer_pairs_list     # list of primer pairs; each element:
                          #   (primer_id, forward_primer_seq,
                          #               reverse_primer_seq)
    max_mismatches = 3    # mismatcjes permitted
    max_offset = 5        # do not search for primers located beyond this
                          #    offset position from start or end.

DATA STRUCTURES:
    For each primer_id:
        forward_fasta_file[primer_id]
        reverse_fasta_file[primer_id]

FUNCTION best_match_with_mismatches(read_seq, primer_seq, max_offset,
                                    max_mismatches, search_direction):
    # search_direction = "5prime" or "3prime"
    primer_len = LENGTH(primer_seq)
    best_start = NONE
    best_mismatches = primer_len + 1

    IF search_direction == "5prime":
        # allow primer to start at positions 0..max_offset
        FOR start_pos FROM 0 TO max_offset:
            IF start_pos + primer_len > LENGTH(read_seq):
                BREAK
            window = SUBSTRING(read_seq, start_pos, primer_len)
            mismatches = HAMMING_DISTANCE(window, primer_seq)
            IF mismatches < best_mismatches:
                best_mismatches = mismatches
                best_start = start_pos

    ELSE IF search_direction == "3prime":
        # allow primer to end within last max_offset+1 positions
        FOR end_pos FROM LENGTH(read_seq) DOWNTO 0:
            start_pos = end_pos - primer_len
            IF start_pos < 0:
                BREAK
```

```
            IF (LENGTH(read_seq) - end_pos) > max_offset:
                CONTINUE    # too far from 3' end
            window = SUBSTRING(read_seq, start_pos, primer_len)
            mismatches = HAMMING_DISTANCE(window, primer_seq)
            IF mismatches < best_mismatches:
                best_mismatches = mismatches
                best_start = start_pos

    IF best_mismatches <= max_mismatches:
        RETURN (best_start, best_mismatches)
    ELSE:
        RETURN (NONE, NONE)


# Main loop: assign read to primer pair and trim
SET max_5prime_offset = 5    # allowed shift from 5'-end
SET max_3prime_offset = 5    # allowed shift from 3'-end

OPEN q30_fastq for reading

# Prepare outputs
FOR each (primer_id, fwd_primer, rev_primer) IN primer_pairs_list:
    OPEN forward_fasta_file[primer_id] for writing
    OPEN reverse_fasta_file[primer_id] for writing

WHILE not end of q30_fastq:
    READ four lines: (hdr, seq, plus, qual)
    IF incomplete record: BREAK

    best_pair_id = NONE
    best_total_mismatches = large_number
    best_trimmed_seq = NONE
    best_orientation = NONE   # "forward" or "reverse"

    # Try all primer pairs
    FOR each (primer_id, fwd_primer, rev_primer) IN primer_pairs_list:

        # 1) Try orientation: read is forward amplicon
        (f_start, f_mm) = best_match_with_mismatches(
                              read_seq = seq,
                              primer_seq = fwd_primer,
                              max_offset = max_5prime_offset,
                              max_mismatches = max_mismatches,
                              search_direction = "5prime")

        (r_start, r_mm) = best_match_with_mismatches(
                              read_seq = seq,
                              primer_seq = REVERSE_COMPLEMENT(rev_primer),
                              max_offset = max_3prime_offset,
                              max_mismatches = max_mismatches,
                              search_direction = "3prime")
```

```
    IF f_start is not NONE AND r_start is not NONE:
        total_mm = f_mm + r_mm
        trimmed_f = SUBSTRING(seq,
                              f_start + LENGTH(fwd_primer),
                              r_start - (f_start + LENGTH(fwd_primer)))
        # 2) Try orientation: read is reverse amplicon (opposite strand)
        seq_rc = REVERSE_COMPLEMENT(seq)

        (f_start_rc, f_mm_rc) = best_match_with_mismatches(
                                    read_seq = seq_rc,
                                    primer_seq = fwd_primer,
                                    max_offset = max_5prime_offset,
                                    max_mismatches = max_mismatches,
                                    search_direction = "5prime")

        (r_start_rc, r_mm_rc) = best_match_with_mismatches(
                                    read_seq = seq_rc,
                                    primer_seq = REVERSE_COMPLEMENT(rev_primer),
                                    max_offset = max_3prime_offset,
                                    max_mismatches = max_mismatches,
                                    search_direction = "3prime")

        IF f_start_rc is not NONE AND r_start_rc is not NONE:
            total_mm_rc = f_mm_rc + r_mm_rc
            trimmed_r = SUBSTRING(seq_rc,
                                  f_start_rc + LENGTH(fwd_primer),
                                  r_start_rc - (f_start_rc + LENGTH(fwd_primer)))

        # 3) Keep the better orientation for this primer pair
        IF f_start is not NONE AND r_start is not NONE:
            IF total_mm < best_total_mismatches:
                best_total_mismatches = total_mm
                best_pair_id = primer_id
                best_trimmed_seq = trimmed_f
                best_orientation = "forward"

        IF f_start_rc is not NONE AND r_start_rc is not NONE:
            IF total_mm_rc < best_total_mismatches:
                best_total_mismatches = total_mm_rc
                best_pair_id = primer_id
                best_trimmed_seq = trimmed_r
                best_orientation = "reverse"

# After checking all primer pairs, write to the corresponding FASTA if matched
IF best_pair_id is not NONE AND LENGTH(best_trimmed_seq) > 0:
    IF best_orientation == "forward":
        out_file = forward_fasta_file[best_pair_id]
    ELSE:
        out_file = reverse_fasta_file[best_pair_id]
```

```
        WRITE ">" + CLEAN_HEADER(hdr, best_pair_id, best_orientation) TO out_file
        WRITE best_trimmed_seq TO out_file
    ELSE:
        # read does not confidently match any primer pair
        # optionally discard or send to separate file
        CONTINUE

CLOSE q30_fastq
FOR each primer_id:
    CLOSE forward_fasta_file[primer_id]
    CLOSE reverse_fasta_file[primer_id]
```

2.4 Collapse reads to haplotypes and counts/frequencies

The FASTA files from the previous step contain primer-trimmed reads where the most abundant sequences appear repeatedly. This step identifies unique sequences (amplicon haplotypes), computes their absolute counts and relative frequencies, and generates new FASTA files where each header contains `HplID|count|frequency`. Here, `HplID` represents the haplotype rank by abundance, `count` is the number of identical reads, and `frequency` is the relative abundance expressed as a percentage.

```
FOR each fasta_filename IN fasta_file_list:

    # Step 1: Read all sequences into hash table
    sequence_to_count = EMPTY_DICTIONARY  # key=sequence, value=count

    OPEN fasta_filename for reading

    WHILE not end of fasta_filename:
        READ line

        IF line starts with ">":
            current_header = TRIM(SUBSTRING(line, 1, END))
            current_seq = ""
        ELSE:
            current_seq = current_seq + UPPERCASE(TRIM(line))

        # Save when hitting next header or EOF
        IF current_seq != "" AND (next_line_starts_with_">" OR EOF):
            IF sequence_to_count[ current_seq ] exists:
                sequence_to_count[ current_seq ] = sequence_to_count[ current_seq ] + 1
            ELSE:
                sequence_to_count[ current_seq ] = 1

    CLOSE fasta_filename

    # Step 2: Compute total reads and prepare output
    total_reads = 0
    FOR each seq, count IN sequence_to_count:
        total_reads = total_reads + count

    # Step 3: Write collapsed FASTA
```

```
    collapsed_filename = output_dir + "/" +
                        BASENAME(fasta_filename) + "_collapsed.fasta"

    OPEN collapsed_filename for writing

    FOR each seq, count IN SORT_BY_COUNT(sequence_to_count, descending=True):
        rel_freq = (count / total_reads) * 100

        header = "hap" + FORMAT_ID(count_rank) +
                "|count=" + count +
                "|freq=" + FORMAT(rel_freq, 2) + "%"

        WRITE ">" + header TO collapsed_filename
        WRITE seq TO collapsed_filename

    CLOSE collapsed_filename

    # Step 4: Write CSV summary
    csv_filename = output_dir + "/" +
                  BASENAME(fasta_filename) + "_haplotype_counts.csv"

    OPEN csv_filename for writing
    WRITE "haplotype_id,count,relative_frequency_pct,sequence_length" TO csv_filename

    rank = 1
    FOR each seq, count IN SORT_BY_COUNT(sequence_to_count, descending=True):
        rel_freq = (count / total_reads) * 100
        seq_len = LENGTH(seq)

        WRITE "hap" + rank + "," + count + "," + rel_freq + "," + seq_len TO csv_filename
        rank = rank + 1

    CLOSE csv_filename

PRINT "Collapsed " + fasta_filename + ": " +
      total_reads + " reads → " +
      LENGTH(sequence_to_count) + " haplotypes"
```

2.5 Load a haplotype fasta file with counts and frequencies

```
INPUT:
    collapsed_fasta_file   # e.g. "amp1_forward_collapsed.fasta"
    output_dir             # optional: where to save parsed data

DATA STRUCTURES:
    haplotypes_list        # list of haplotype objects
    haplotype_stats        # summary statistics

STRUCT haplotype:
    id                   # "hap1", "hap2", etc.
    count                # integer read count
    frequency_pct        # float relative frequency
```

```
    sequence              # DNA sequence string
    sequence_length       # length of amplicon

OPEN collapsed_fasta_file for reading

current_header = ""
current_seq = ""
haplotypes_list = EMPTY_LIST

WHILE not end of collapsed_fasta_file:
    READ line

    IF line starts with ">":
        # Parse previous haplotype if exists
        IF current_header != "":
            new_haplotype = PARSE_HEADER(current_header)
            new_haplotype.sequence = current_seq
            new_haplotype.sequence_length = LENGTH(current_seq)
            haplotypes_list.APPEND(new_haplotype)

        # Start new record
        current_header = TRIM(SUBSTRING(line, 1, END))
        current_seq = ""

    ELSE:
        # Sequence line
        current_seq = current_seq + UPPERCASE(TRIM(line))

# Don't forget last record
IF current_header != "":
    new_haplotype = PARSE_HEADER(current_header)
    new_haplotype.sequence = current_seq
    new_haplotype.sequence_length = LENGTH(current_seq)
    haplotypes_list.APPEND(new_haplotype)

CLOSE collapsed_fasta_file

## Helper: Parse header metadata
FUNCTION PARSE_HEADER(header_string):
    haplotype = NEW(haplotype)

    # Expected format: ">hap1|count=1250|freq=12.50%"
    parts = SPLIT(header_string, "|")

    haplotype.id = SPLIT(parts[0], " ")[0]  # "hap1"

    FOR each part IN parts[1:]:
        IF part starts with "count=":
            haplotype.count = INTEGER(SUBSTRING(part, 6, END))
        ELSE IF part starts with "freq=":
            # Remove "%" and convert to float
```

```
            freq_str = SUBSTRING(part, 5, LENGTH(part)-1)
            haplotype.frequency_pct = FLOAT(freq_str)

    RETURN haplotype

## Optional: Compute summary statistics
total_haplotypes = LENGTH(haplotypes_list)
total_reads = SUM(haplotype.count for haplotype IN haplotypes_list)
dominant_haplotype_freq = MAX(haplotype.frequency_pct for haplotype IN haplotypes_list)

PRINT "Loaded " + total_haplotypes + " haplotypes from " +
      total_reads + " total reads"
PRINT "Shannon diversity: " + COMPUTE_SHANNON(haplotypes_list)
PRINT "Dominant haplotype: " + dominant_haplotype_freq + "%"

## Optional: Save parsed data as TSV
tsv_filename = output_dir + "/haplotypes_parsed.tsv"
OPEN tsv_filename for writing
WRITE "id\tcount\tfrequency_pct\tsequence_length\tsequence" TO tsv_filename

FOR each haplotype IN SORT_BY_FREQUENCY(haplotypes_list, descending=True):
    WRITE haplotype.id + "\t" +
          haplotype.count + "\t" +
          haplotype.frequency_pct + "\t" +
          haplotype.sequence_length + "\t" +
          haplotype.sequence TO tsv_filename

CLOSE tsv_filename
```

2.6 Recommended R libraries and functions

R libraries ShortRead (Morgan et al. 2009), Biostrings (Pagès et al. 2024) and ape (Paradis and Schliep 2019) are very useful.

ShortRead offers the function FastqStreamer() to set a steamer on a fastq file, which may be loaded by chuncks with function yield(). From each fastq chunk base calling quality scores are obtained with the method quality(), whereas the reads are obtained with the method sread(). The following snippet shows how a FASTQ file may be processed in chuncks of chunkSize reads.

```
library(ShortRead)

strm <- FastqStreamer(FileName,n=chunckSize)
while(length(sqq <- yield(strm))) {
    ###  Base calling quality scores. Codi ASCII-33
    phrsc <- quality(sqq)
    ###  Reads
    seqs <- sread(sqq)
    ### Process reads in chunck
    . . . . . . . . . . . . . . .
}
close(strm)
```

Biostrings offers multiple functions to manipulate fasta and fastq files. In particular aligning

primers may be easily done with the help of function `vmatchPattern()`, and trimming the primers with the function `subseq()`. `vmatchPattern()` acts on a `DNAStringSet` so that a single call results in the alignment of a given primer to all sequences in the object.

```
up.matches <-
 vmatchPattern(pattern=forward_primer_seq,      # Primer sequence to align
              subject=subseq(seqs,              # DNAStringSet
                             start=target.io,   # First window position
                             end=target.in),    # Last window position
              max.mismatch=max.missmatches,     # Allowed mismatches
              fixed=FALSE)

# Indices of sequences in the DNAStringSet where the primer aligned
#  within the expected window of positions
idx <- which(elementLengths(up.matches)>=1)
```

DNA fasta files may be read and written with functions `readDNAStringSet()` and `writeDNAStringSet()`, and amino acid sequences with `readAAStringSet()` and `writeAAStringSet()`.

The reads in a fasta file may be collapsed to haplotypes and read counts using the following function:

```
###  Collapse reads to haplotypes and counts
###  Returns a list with haplotypes and corresponding read counts
Reads_to_Haplotypes <- function(reads)
{
  sqtbl <- sort(table(as.character(reads)),decreasing=TRUE)
  bseqs <- names(sqtbl)
  names(bseqs) <- 1:length(bseqs)
  nr <- as.integer(sqtbl)
  return(list(hpls=bseqs,nr=nr))
}
```

A function to read fasta files with read counts and frequencies in sequence headers. With headers formatted as `>HplID|counts|freq`

```
### Read fasta file with counts and frequencies in sequence headers
### Returns a list with haplotype IDs, read counts, relative frequencies,
###  and the haplotypes as a DNAStringSet object
read.ampl.hpls <- function(flnm)
{ seqs <- readDNAStringSet(flnm)
  parts <- t(sapply(names(seqs),function(str) strsplit(str,split='\\|')[[1]]))
  nr <- as.integer(parts[,2])
  frq <- as.numeric(parts[,3])
  return( list(ID=parts[,1],nr=nr,frq=frq,hpls=seqs) )
}
```

References

Gregori, J., et al. (2022) 'Quasispecies Fitness Partition to Characterize the Molecular Status of a Viral Population. Negative Effect of Early Ribavirin Discontinuation in a Chronically Infected HEV Patient.', *Int J Mol Sci*, 23/23: 14654:1–17, https://doi.org/10.3390/ijms232314654.

Gregori, J., et al. (2024) 'In-Host Flat-Like Quasispecies: Characterization Methods and Clinical Implications', *Microorganisms*, 12/5: 1011:1–14, https://doi.org/10.3390/microorganisms12051011.

Magoc, T., and S. L. Salzberg (2011) 'FLASH: Fast Length Adjustment of Short Reads to Improve Genome Assemblies', *Bioinformatics*, 27: 2957–63, https://doi.org/10.1093/bioinformatics/btr507.

Morgan, Martin, et al. (2009) 'ShortRead: A Bioconductor Package for Input, Quality Assessment and Exploration of High-Throughput Sequence Data', *Bioinformatics*, 25: 2607–8, https://doi.org/10.1093/bioinformatics/btp450.

Pagès, Hervé, et al. (2024) *Biostrings: Efficient Manipulation of Biological Strings*, https://bioconductor.org/packages/Biostrings.

Paradis, Emmanuel, and Klaus Schliep (2019) 'Ape 5.0: An Environment for Modern Phylogenetics and Evolutionary Analyses in R', *Bioinformatics*, 35: 526–8, https://doi.org/10.1093/bioinformatics/bty633.

Wickham, Hadley, et al. (2019) 'Welcome to the tidyverse', *Journal of Open Source Software*, 4/43: 1686, https://doi.org/10.21105/joss.01686.
